# *CLAVATA* Was a Genetic Novelty for the Morphological Innovation of 3D Growth in Land Plants

**DOI:** 10.1016/j.cub.2018.05.068

**Published:** 2018-08-06

**Authors:** Chris D. Whitewoods, Joseph Cammarata, Zoe Nemec Venza, Stephanie Sang, Ashley D. Crook, Tsuyoshi Aoyama, Xiao Y. Wang, Manuel Waller, Yasuko Kamisugi, Andrew C. Cuming, Péter Szövényi, Zachary L. Nimchuk, Adrienne H.K. Roeder, Michael J. Scanlon, C. Jill Harrison

**Affiliations:** 1 Plant Sciences Department, Cambridge University, Downing Street, Cambridge CB2 3EA, UK; 2 Plant Biology Section, School of Integrative Plant Science, Cornell University, Tower Road, Ithaca, NY 14853, USA; 3 Weill Institute for Cell and Molecular Biology, Cornell University, Ithaca, NY 14853, USA; 4 School of Biological Sciences, University of Bristol, 24 Tyndall Avenue, Bristol BS8 1TQ, UK; 5 Department of Biology, University of North Carolina at Chapel Hill, Chapel Hill, NC 27599, USA; 6 Department of Systematic and Evolutionary Botany, University of Zurich, Zollikerstrasse 107, 8008 Zurich, Switzerland; 7 Centre for Plant Sciences, Faculty of Biological Sciences, University of Leeds, Leeds LS2 9JT, UK; 8 Curriculum in Genetics and Molecular Biology, University of North Carolina at Chapel Hill, Chapel Hill, NC 27599, USA

**Keywords:** land plant evolution, plant evo-devo, plant cell division plane, 3D growth, CLAVATA

## Abstract

How genes shape diverse plant and animal body forms is a key question in biology. Unlike animal cells, plant cells are confined by rigid cell walls, and cell division plane orientation and growth rather than cell movement determine overall body form. The emergence of plants on land coincided with a new capacity to rotate stem cell divisions through multiple planes, and this enabled three-dimensional (3D) forms to arise from ancestral forms constrained to 2D growth. The genes involved in this evolutionary innovation are largely unknown. The evolution of 3D growth is recapitulated during the development of modern mosses when leafy shoots arise from a filamentous (2D) precursor tissue. Here, we show that a conserved, CLAVATA peptide and receptor-like kinase pathway originated with land plants and orients stem cell division planes during the transition from 2D to 3D growth in a moss, *Physcomitrella*. We find that this newly identified role for CLAVATA in regulating cell division plane orientation is shared between *Physcomitrella* and *Arabidopsis*. We report that roles for CLAVATA in regulating cell proliferation and cell fate are also shared and that CLAVATA-like peptides act via conserved receptor components in *Physcomitrella*. Our results suggest that *CLAVATA* was a genetic novelty enabling the morphological innovation of 3D growth in land plants.

## Highlights

• CLAVATA originated in the last common ancestor of land plants
• CLAVATA regulates cell proliferation, fate, and growth in *Physcomitrella*
• CLAVATA orients cell division planes in *Physcomitrella* and *Arabidopsis*
• CLEs act via receptors that are conserved between *Physcomitrella* and *Arabidopsis*

## Teaser

Whitewoods, Cammarata, et al. show that a conserved CLAVATA (CLV) pathway arose in the last common ancestor of land plants. CLV regulates cell division plane orientation during the 2D to 3D growth transition in a moss, and roles for CLV are shared between mosses and flowering plants, suggesting that CLV enabled 3D growth to arise in land plants.

## Introduction

**Figure 1:**
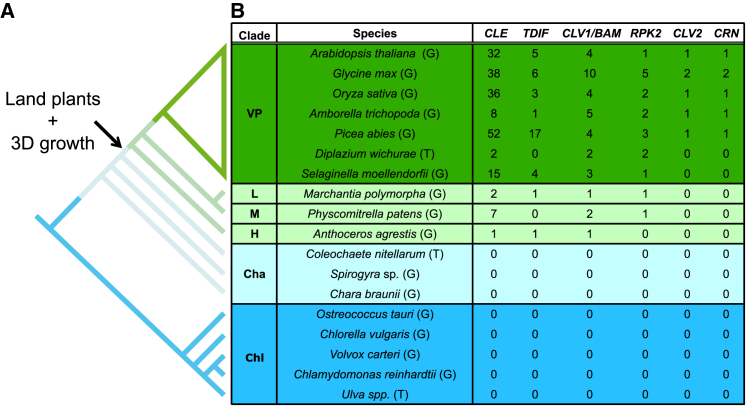
The CLV Pathway Originated in the Last Common Ancestor of Land Plants, Concomitantly with 3D Growth (A) Phylogenetic relationships among land plants and their freshwater algal sister lineages redrawn from [4] and [5], respectively. Although chlorophytes and charophytes undergo stem cell divisions in a single orientation (2D growth), land plants undergo stem cell divisions in multiple orientations to generate elaborate three-dimensional forms (3D growth). (B) The number of CLV pathway homologs was determined by BLAST against genome or draft genome (G) and transcriptome (T) databases as described in STAR Methods. Cha, charophytes; Chl, chlorophytes; H, hornworts; L, liverworts; M, mosses; VP, vascular plants. See also Figures S1, S2, and S3 and Table S1.

The conquest of land was enabled by a series of innovations that allowed plant forms to radiate and occupy new volumes of space in the sub-aerial environment [1]. Among these, the innovation of shooting systems with organs positioned radially around an upright stem stands out as a primer for massively increased plant productivity and diversity [1]. Such three-dimensional (3D) growth forms first arose as a consequence of a novel stem cell function gained by land plants, namely the capacity to rotate stem cell divisions through multiple plane orientations [1, 2, 3]. The algal sister lineages of land plants are unable to rotate stem cell divisions through multiple planes and are therefore generally constrained to smaller filamentous or mat-like (two-dimensional [2D]) growth forms (Figure 1A) [1, 3]. The evolutionary transition from 2D to 3D growth is recapitulated during the development of modern mosses when a branching, filamentous (protonemal) precursor tissue (2D) gives rise to 3D gamete-producing leafy shoots (gametophores) [6]. Previous studies have shown that gametophores and filament branches initiate similarly as hemispherical outgrowths from parent filaments and that their divergent 2D or 3D fates are specified stochastically by APETALA2-type (APB) transcription factor activity [7]. During a single-celled stage of outgrowth development, persistent APB activity and cell swelling mark a switch to gametophore fate (3D), whereas loss of APB activity marks filament fate (2D) [6, 7]. A strongly oblique cell division is the first reliable morphological marker of gametophore development [6, 7]. This is followed by a second oblique apical cell division, which is approximately perpendicular to the first, after which division planes rotate during two successive rounds of division to establish a tetrahedral apical stem cell [6]. The tetrahedral apical cell divides in spiraling planes to replace itself and produce daughter cells that generate the 3D gametophore axis and leaves [6]. The mechanisms regulating such novel and rotating stem cell division plane orientations during evolutionary and developmental transitions to 3D growth are unknown.

In *Arabidopsis*, the *CLAVATA* (*CLV*) and *WUSCHEL* (*WUS*) pathways act in a feedback loop to regulate many aspects of stem cell function, including cell fate [8, 9], proliferation [9, 10, 11], and growth [12]. *CLV3* encodes a small, secreted peptide that is expressed in the upper cell layers of the central zone and can move throughout the meristem [13, 14, 15]. *CLV1* is expressed in the underlying cell layers of the central zone and encodes a receptor-like kinase that acts as a receptor for CLV3 [11, 16] in conjunction with CLV2, CORYNE (CRN), RECEPTOR-LIKE PROTEIN KINASE 2 (RPK2), and BARELY ANY MERISTEM (BAM) [17, 18]. *WUS* activity promotes meristem cell proliferation [19], and CLV signaling restricts the size of the *WUS* expression domain [13]. WUS acts non-cell autonomously, moving from the organizing center to the uppermost meristem cell layers, where it promotes *CLV3* expression [20], thereby closing the feedback loop that maintains meristem size.

## Results

### The CLAVATA Pathway Originated in the Last Common Ancestor of Land Plants

To determine how the CLV pathway evolved and identify potential roles for CLV in *Physcomitrella* stem cell function, we first queried publicly accessible genome and transcriptome databases from a wide range of green algae and land plants for *CLV3*-like (*CLE*), *CLV1/BAM*, *RPK2*, *CLV2*, and *CRN* homologs (Figure 1B; Table S1). We found no CLV pathway homologs in the chlorophyte or charophyte algae sampled but found at least one *CLE* homolog and one *CLV1*/*BAM* homolog in each early-diverging bryophyte lineage and all other land plants, suggesting that the core CLV signaling module comprises at least one CLE peptide and a CLV/BAM receptor-like kinase. *RPK2* homologs were present in all land plants sampled except the hornwort, *Anthoceros agrestis*. In *Physcomitrella*, we identified seven genes with a conserved *CLE* domain encoding a 12-amino-acid peptide motif similar to CLV3, but sequences outside the conserved CLE domain were divergent (Figure 1; Table S1). The genome encodes four CLV3-like peptides: *PpCLE*s *1*, *2*, and *3* encode the peptide motif RMVPTGPNPLHN; *PpCLE4* encodes the motif RMVPSGPNPLHN; *PpCLE*s *5* and *6* encode the motif RLVPTGPNPLHN; and *PpCLE7* encodes the motif RVVPTGPNPLHN. Neighbor-joining phylogenetic reconstructions showed that, although hornworts and liverworts have *CLE*s resembling the tracheary element differentiation inhibitory factor (TDIF)-like *CLE*s that regulate vascular development in *Arabidopsis*, *Physcomitrella* does not, consistent with an evolutionary loss in mosses (Figure S1; Data S1). Receptor-like kinase phylogenies were reconstructed by maximum likelihood analysis using amino acids from the conserved kinase domain (Figures S2 and S3; Data S2 and S3). Clades encompassing *CLV1*/*BAM*-like sequences from each land plant lineage or containing *RPK2*-like sequences from each lineage except hornworts were resolved. Both *CLV1*/*BAM* and *RPK2* phylogenies were broadly congruent with current hypotheses of land plant evolution [4, 21], thereby indicating orthology. Two *Physcomitrella* genes were incorporated in the *CLV1*/*BAM* clade, and these were named *Physcomitrella CLAVATA1a* and *1b* (*PpCLV1a* and *PpCLV1b*). One *RPK2* homolog was found and named *PpRPK2*, but no *CLV2* or *CRN* homologs were found. These sequence data indicate that the core components of the CLV pathway first arose in the last common ancestor of land plants, alongside the evolutionary innovation of 3D growth [22].

### *Physcomitrella* CLAVATA Pathway Components Are Expressed during the 3D Growth Phase

**Figure 2:**
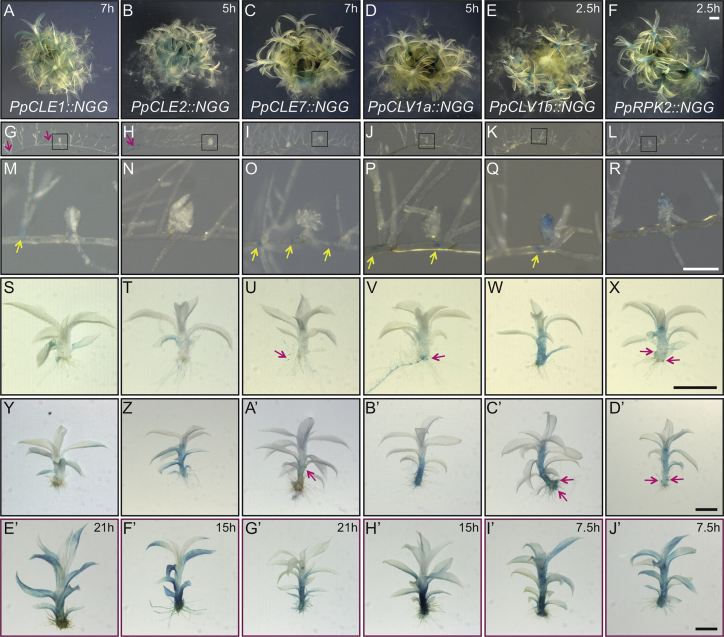
CLV Pathway Components Are Expressed in *Physcomitrella* Protonemata and Gametophores (A– J’) GUS staining of *PpCLE1::NGG* (A, G, M, S, Y, and E’), *PpCLE2::NGG* (B, H, N, T, Z, and F’), *PpCLE7::NGG* (C, I, O, U, A’, and G’), *PpCLV1a::NGG* (D, J, P, V, B’, and H’), *PpCLV1b::NGG* (E, K, Q, W, C’, and I’), and *PpRPK2::NGG* (F, L, R, X, D’, and J’) lines revealed complex expression dynamics. Although *PpCLE::NGG* and *PpCLV1a::NGG* signal accumulated in protonemal tissues close to buds (G–J and M–P; arrows indicate signal in protonemata), *PpCLV1b::NGG* and *PpRPK2::NGG* signal accumulated mainly in the apical region of buds (Q and R). At two later stages of gametophore development (S–X and Y–J’), all promoters were active in gametophores, although the patterns and intensity of activity varied between reporters and by developmental stage. *PpCLE1::NGG* lines stained most strongly in leaves (S, Y, and E’), *PpCLE2::NGG* lines stained most strongly in leaves and gametophore bases (T, Z, and F’), and *PpCLE7::NGG* lines accumulated stain in rhizoid tips (arrow in U), leaf bases (arrow in A’), and hairs around the apex and the gametophore axis (G’). *PpCLV1a::NGG* lines did not stain intensely at early stages of gametophore development (P and V) but accumulated signal in gametophore axes and leaves at later stages (B' and H'). In contrast, *PpCLV1b::NGG* and *PpRPK2::NGG* lines accumulated signal in gametophore axes and leaves from early stages of development (W and X), and strong signal was detected in branches initiating at gametophore bases (arrows in X, C’, and D’). All tissues in (A)–(D’) were stained in a solution containing 0.5 mM FeCN for times specified in (A)–(F), and gametophores in (E’)–(J’) were stained three times longer in a solution containing 2 mM FeCN. The scale bars in (A)–(F) represent 1 mm, the scale bars in (M)–(R) represent 100 μm, and insets in (G)–(L) indicate position of buds in (M)–(R). The scale bars in (S)–(J’) represent 1 mm. See also Methods S1 and Table S4.

To investigate *Physcomitrella* CLV activity, we first analyzed gene expression patterns in relation to the transition between 2D filamentous and 3D gametophore growth (Figures 2, S4, and S5). By RT-PCR, we detected *PpCLE1*, *2*, and *7* peptide-encoding gene expression in gametophores (Figure S4). We were unable to detect expression of *PpCLE*s *3*, *4*, and *5*, but we found *PpCLE6* expression in protonemal filaments. Receptor-encoding genes *PpRPK2*, *PpCLV1a*, and *PpCLV1b* were co-expressed in gametophores, although *PpRPK2* expression was evident earlier than *PpCLV1a* and *PpCLV1b* in day 10 filamentous tissues (Figure S4). These results were broadly consistent with reports from transcriptome data (Figure S5) [23, 24]. We also constructed *promoter::NLSGFPGUS* (*promoter::NGG*) fusion lines for *PpCLE1*, *PpCLE2*, *PpCLE7*, *PpCLV1a*, *PpCLV1b*, and *PpRPK2* as RT-PCR showed that these 6 genes were upregulated at around the time of gametophore initiation (see Strategy for generation of *promoter::NLSGUSGFP* reporter lines in Methods S1; Figure 2). In 3-week-old spot cultures (Figures 2A–2F), *PpCLE1::NGG*, *PpCLE2::NGG*, *PpCLE7::NGG*, and *PpCLV1a::NGG* lines accumulated local signal in various protonemal cell types around the buds (Figures 2G–2J and 2M–2P). *PpCLV1b::NGG* and *PpRPK2::NGG* lines accumulated signal in buds, and the signal was strongest toward the apex (Figures 2K, 2L, 2Q, and 2R). Whereas all lines accumulated signal in gametophore axes and leaves (Figures 2S–2J’), there was variation in the pattern, timing, and intensity of signal accumulation between lines. Notably, *PpCLE1::NGG*, *PpCLE2::NGG*, *PpCLE7::NGG*, and *PpCLV1a::NGG* signal accumulation in gametophores was delayed with respect to *PpCLV1b::NGG* and *PpRPK2::NGG* lines (Figures 2M–2X). These beta-glucuronidase (GUS) accumulation patterns suggested highly dynamic foci of expression for *PpCLE*s *1*, *2*, and *7* and *PpCLV1a*, *PpCLV1b*, and *PpRPK2* in *Physcomitrella*, prompting us to investigate roles for CLV pathway components in gametophore initiation and development, i.e., during the transition to 3D growth.

### *Physcomitrella* Mutants Lacking CLAVATA Function Have a Defective 2D to 3D Growth Transition

**Figure 3:**
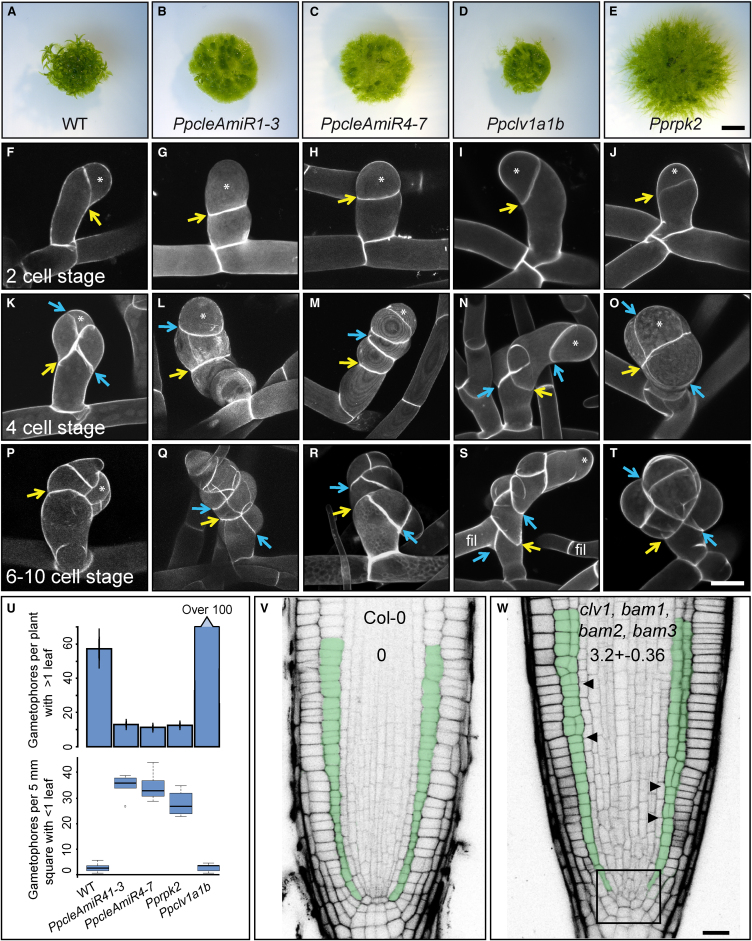
The CLV Pathway Regulates Cell Division Plane Orientations during 3D Growth in *Physcomitrella* and *Arabidopsis* (A–E) Although WT plants (A) developed many normal gametophores, *PpcleAmiR1-3* (B), *PpcleAmiR4-7* (C), *Ppclv1a1b* (D), and *Pprpk2* (E) mutants had no obvious gametophores. The scale bar represents 0.35 cm. (F–T) *PpcleAmiR1-3*, *PpcleAmiR4-7*, *Ppclv1a1b*, and *Pprpk2* mutants have cell division plane defects at the onset of 3D morphogenesis. (F–J) The first division of each bud is indicated by a yellow arrow and is set at a strongly oblique angle in WT (F), *Ppclv1a1b* (I), and *Pprpk2* (J) plants, but is weakly oblique in *PpcleAmiR1-3* (G) and *PpcleAmiR4-7* (H) mutants. (K–O) Whereas (K) the second division (blue arrow) from the apical cell (asterisk) is normally oblique and roughly perpendicular to the first, in *PpcleAmiR1-3* (L), *PpcleAmiR4-7* (M) and *Ppclv1a1b* (N) mutants, it is roughly parallel to the first. *Pprpk2* (O) mutants look normal at this stage. (P–T) The stereotypical divisions that normally generate the tetrahedral shape of the gametophore apical cell at the 6- to 10-celled stage of development (P) are misset in *PpcleAmiR1-3* (Q), *PpcleAmiR4-7* (R), and *Ppclv1a1b* (S), and *Pprpk2* (T) mutants. The scale bar represents 30 μm. (U) Bar chart and boxplot showing that gametophore initiation was disrupted in *PpcleAmiR1-3*, *PpcleAmiR4-7*, *PpCLV1a1b*, and *Pprpk2* mutants. The number of gametophores with >1 leaf was counted in 5 WT and mutant plants from a single line representing each mutant class. Gametophore buds with <1 leaf were counted from a 5-mm^2^ area in 3 WT and mutant plants from a single line representing each mutant class. ANOVA, Tukey’s Honest Significant Difference (HSD) test; p < 0.005. (V and W) Confocal micrographs of WT (Col-0) (V) and *clv1*/*bam1*/*bam2*/*bam3* mutant (W) root tips showing disordered cell division plane orientations in the meristem and ground tissue layers. The box in (W) indicates the meristem, and arrowheads indicate the developmental onset of abnormal periclinal division plane orientations in the cortex layer (shaded green). The scale bar represents 20 μm. See also Figure S6, Methods S1, and Table S4.

To identify the functions of CLV pathway components, we used artificial microRNAs (AmiRNAs) to silence expression of *PpCLEs 1*, *2*, and *3* and *PpCLEs 4*, 5, 6, and *7* (see Strategy for generating *PpcleAmiR* lines in Methods S1). We used a CRISPR-Cas9 approach to disrupt the function of *PpCLV1* paralogs (see CRISPR/Cas9 strategy for generating *Ppclv1* mutants in Methods S1), and gene targeting was used to abrogate *PpRPK2* function (see Strategy for generating *Pprpk2* KO lines in Methods S1). *PpcleAmiR1-3*, *PpcleAmiR4-7*, *Ppclv1a1b*, and *Pprpk2* lines were able to form dense protonemal tissues and thus had a relatively normal 2D growth phase (Figures 3A–3E). However, all four mutant classes had defective development during the 3D growth phase, with a reduction in the overall number of mature gametophores and defects in gametophore development (Figures 3A–3E and 3U). Further examination revealed many more gametophore buds with 1 or fewer leaves in *PpcleAmiR1-3*, *PpcleAmiR4-7*, and *Pprpk2* mutants than in wild-type (WT) plants (Figure 3U), and *Ppclv1a1b* mutants had many small gametophores arrested at a later stage of development (Figure 3U). These data suggested early defects in gametophore development with potential feedback onto the gametophore initiation process. To determine how WT and mutant phenotypes diverged during development, we imaged gametophore buds at 2-cell, 4-cell, and a later stage of bud development [6] (Figures 3F–3T). Although WT gametophores initiated normally and showed characteristic oblique cell division plane orientations, the plane of the first division was strongly disrupted in *PpcleAmiR1-3* and *PpcleAmiR4-7* mutants, and it was set at a shallow angle relative to the main growth axis (compare Figure 3F to Figures 3G and 3H). A second round of cell division from the apical cell also had misset division planes that were frequently parallel rather than perpendicular to the first division plane, and a subset of gametophores therefore formed finger-like projections in place of gametophores (compare Figure 3K to Figures 3L and 3M). At developmental stages where the tetrahedral shape of the apical cell is normally established [6], mutants also had defects indicating problems with growth and cell fate specification, appearing to reiterate divisions normally characteristic of the first gametophore initial (compare Figure 3P to Figures 3Q and 3R). *Ppclv1a1b* mutant phenotypes diverged from WT after the 2-cell stage, subsequently showing a similar pattern of division to *PpcleAmiR1-3* and *PpcleAmiR4-7* mutants (Figures 3K–3N and 3P–3S), and some cells reverted to filament identity (Figure 3S). *Pprpk2* mutant defects were less severe than *Ppcle* and *Ppclv1a1b* defects at the earliest developmental stages, and at later stages, swollen cell shapes suggested growth defects as well as division plane defects (Figure 3T). The mutant phenotypes above suggest key roles for the *Physcomitrella* CLV pathway in modulating cell division planes, cell fate, growth, and proliferation during the 2D-3D developmental transition. The formation of long projections of swollen cells in *Ppcle* mutants (e.g., Figures 3L and 3M) suggests that gametophore identity is attained normally, as cell swelling is a characteristic of gametophore rather than filament initials. The manifestation of plane orientation defects in the first division suggests that WT and mutant gametophore development diverge at the single-celled stage, after cell fate is specified.

### Roles for CLAVATA in Regulating Cell Division Plane Orientation Are Conserved between *Physcomitrella* and *Arabidopsis*

As roles for CLV in cell division plane orientation were previously unreported, we sought to identify conservation of function with *Arabidopsis*. To this end, we examined *Arabidopsis clv1*/*bam1*/*bam2*/*bam3* quadruple mutant meristems, in which the function of the entire *CLV*/*BAM* gene clade is lost [25]. Whereas division plane orientations are normally stereotypic in root meristems, we detected strongly disordered planes in the stem cell niche and ground tissue layers of *clv1/bam1/bam2/bam3* mutant roots (Figures 3V, 3W, and S6). Thus, a newly identified role for CLV in cell division plane orientation is conserved between *Physcomitrella* and *Arabidopsis*.

### *Physcomitrella* Mutants with Disrupted CLV Function Have Defective Gametophore Development

**Figure 4:**
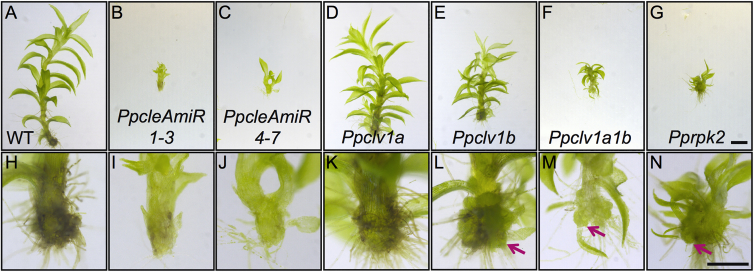
Gametophore Phenotypes in *PpAmiRcle*, *Ppclv1*, and *Pprpk2* Mutants (A–G) Light micrographs showing height differences between WT (A), *PpcleAmiR1-3* (B), *PpcleAmiR4-7* (C), *Ppclv1a* (D), *Ppclv1b* (E), *Ppclv1a1b* (F), and *Pprpk2* (G) gametophores dissected from 1-month-old plants. The scale bar represents 1 mm. (H–N) Light micrographs of gametophore bases with arrows showing overproliferation in *Ppclv1b* (L), *Ppclv1a1b* (M), and *Pprpk2* (N) mutants. WT (H), *PpcleAmiR1-3* (I), *PpcleAmiR4-7* (J) and *Ppclv1a* (K) gametophores show no such overproliferation. The scale bar represents 0.5 mm.

**Figure 5:**
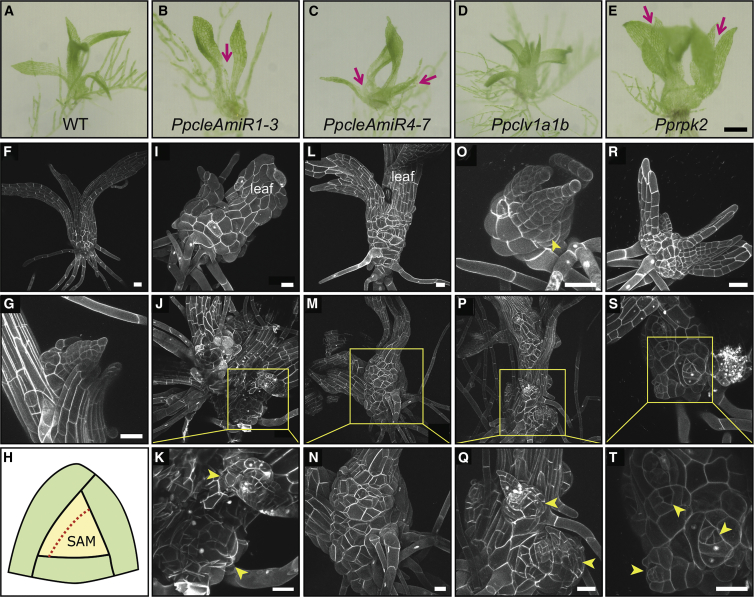
Overproliferation Phenotypes in *PpAmiRcle*, *Ppclv1*, and *Pprpk2* Mutants (A–E) Light micrographs of mutant gametophore morphology showing that gametophores (B) arrest, (C and E) develop multiple axes (pink arrows), and (C–E) develop swollen bases relative to (A) WT plants. The scale bar represents 200 μm. (F and G) Confocal micrographs showing (F) overall gametophore morphology and (G) a branch initiating in a leaf axil in WT plants. (H) Schematic showing *Physcomitrella* gametophore apex organization with an apical cell (pale yellow) and rotating division plane orientations. (I–T) Confocal micrographs showing (I–K) *PpcleAmiR1-3* mutant gametophore morphologies, with (I) overproliferation at the gametophore base and (J and K) disorganized growth with ectopic meristems. (L–N) *PpcleAmiR4-7* mutant gametophore morphologies with (L) split leaf phenotypes and (M and N) meristem overproliferation and termination. (O–Q) *Ppclv1a1b* mutant gametophore morphology (O), with multiple growth axes and multiple meristems at the gametophore base (P and Q). (R–T) *Pprpk2* mutant gametophore morphology with multiple growth axes (R) and multiple meristems at the gametophore base (S and T). Yellow arrowheads indicate regions of overproliferation or ectopic meristems. Yellow boxes show regions magnified from (J), (M), (P), and (S) to (K), (N), (Q), and (T). The scale bars represent 50 μm.

In *Arabidopsis* and other flowering plants, the CLV pathway is known for its role in maintaining the size of the meristematic stem cell pool [26], and increases in the number of stem cells lead to highly enlarged meristems in both *clv1* and *clv3* (*cle*) mutants. However, *Physcomitrella* does not fit the *Arabidopsis* paradigm of meristem function because the shoot apex comprises a single apical stem cell. The apical cell cleaves merophyte daughter cells in a spiral pattern, and merophytes subsequently divide to generate leaf initials and stem tissues [6]. To investigate whether roles for CLV in regulating stem cell function are conserved between *Physcomitrella* and *Arabidopsis*, we imaged one of the largest gametophores from 1-month-old WT and mutant plants using light and confocal microscopy and found that mutant gametophores were reduced in height and had developmental defects (Figure 4). Although *PpcleAmiR1-3*, *PpcleAmiR4-7*, and *Pprpk2* mutants were most severely reduced in height (Figures 4B, 4C, and 4G), *Ppclv1a* and *Ppclv1b* mutants had milder defects (Figures 4D and 4E). *PpcleAmiR1-3*, *PpcleAmiR4-7*, *Ppclv1a1b*, and *Pprpk2* mutants had defective leaf development, and *Ppclv1b*, *Ppclv1a1b*, and *Pprpk2* mutants also had strong cell fate and/or proliferation defects, developing a callus-like mass at the gametophore base (Figures 4L–4N). Closer inspection revealed that these masses arose by the activity of many ectopic apical cells at the gametophore base (Figure 5). These loss-of-function data suggest that CLV has roles in regulating stem cell function that are conserved between *Physcomitrella* and *Arabidopsis*.

### CLE Peptides Can Suppress Cell Proliferation in *Physcomitrella* Gametophores

**Figure 6:**
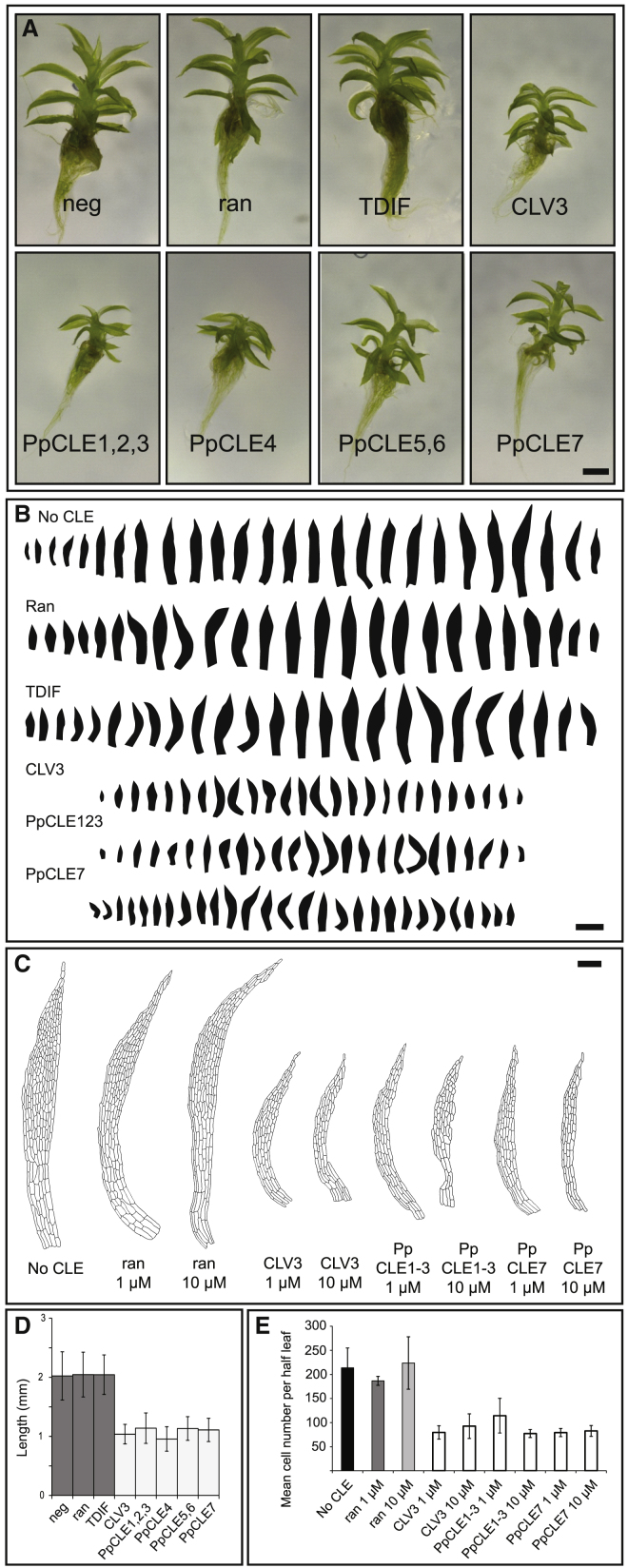
*Physcomitrella* CLEs Suppress Cell Proliferation (A) Treatment of *Physcomitrella* plants with 1 μM CLV3-like CLEs from *Arabidopsis* and *Physcomitrella*, but not TDIF-like CLEs, causes gametophore and leaf stunting. The scale bar represents 100 μm. (B) Leaf series from gametophores treated with CLEs expressed during gametophore development. The scale bar represents 1 mm. (C) Cell outlines of half-leaves in CLE-treated gametophores (leaf 9 was used). The scale bar represents 100 μm. (D) Height measured from ≥25 gametophores treated with CLEs (n ≥ 25; ANOVA; Tukey’s HSD; p < 0.005). (E) Leaf 9 cell numbers in CLE-treated half-leaves (n = 3; ANOVA; Tukey’s HSD; p < 0.05). See also Figure S7.

To further assay conservation in CLV function, we undertook a gain-of-function approach by applying synthetic CLE peptides to growing plants (Figures 6 and S7). After 4 weeks of growth, we found that treatment with a 1-μM concentration of CLE had no appreciable effect on plant spread or the number of gametophores initiating, indicating that protonemal development is normal (Figure S7). However, although solute controls, a randomized peptide and *Arabidopsis* CLE41 (a TDIF CLE) have no appreciable effect on gametophore development, *Arabidopsis* CLV3 and all of the *Physcomitrella* CLEs cause gametophore dwarfing and a strong reduction in leaf size correlating with a reduction in leaf cell number (Figure 6). Although this phenotype superficially resembles the stunted gametophore phenotypes of *PpcleAmiR1-3* and *PpcleAmiR4-7* mutants (Figures 4B and 4C), we found no evidence of developmental arrest or meristematic overproliferation following CLE application and no difference in the number of gametophores initiating was detected following CLE treatment (data not shown). These data show that CLEs act through a conserved signaling module to regulate cell proliferation specifically during the 3D growth phase in *Physcomitrella*.

### CLE Peptides Can Act through Receptor Components that Are Conserved between *Physcomitrella* and *Arabidopsis*

**Figure 7:**
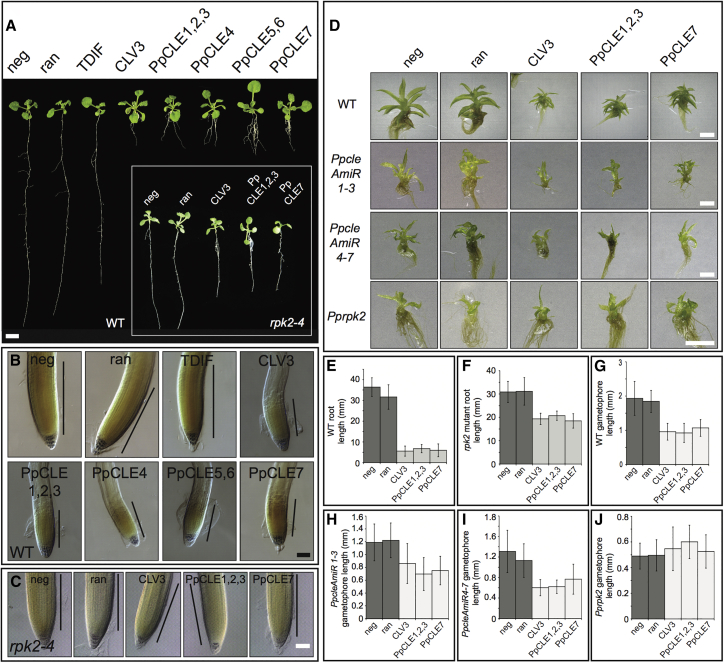
*Physcomitrella* CLE Peptides Act via a Conserved Receptor Machinery (A and B) Treatment of *Arabidopsis* seedlings with 1 μM CLV3-like CLEs from *Arabidopsis* and *Physcomitrella*, but not TDIF-like CLEs, suppresses root meristem proliferation in WT *Arabidopsis* (n = 30; ANOVA; Tukey’s HSD; p < 0.0005). The scale bar in (A) represents 1 cm and scale bar in (B) represents 100 μm; black bars in (B) represent approximate position and extent of root meristem. (C and inset in A) *Arabidopsis rpk2-4* mutants are resistant to treatment with 1 μM CLE peptides; n ≥ 15; ANOVA; Tukey’s HSD; p < 0.0005. The scale bar represents 100 μm in (C). (D) Gametophores in plants treated with 10 μM CLEs are stunted in WT, *PpcleAmiR1-3*, and *PpcleAmiR4-7* mutants, but not *Pprpk2* mutants (n ≥ 20; ANOVA; Tukey’s HSD; p < 0.0005). The scale bar represents 100 μm. (E and F) Quantitative data on root length in WT (E) and *rpk2* mutant plants (F), supporting inferences from images shown in (A). (G–J) Quantitative data on gametophore length in WT (G), *PpcleAmiR1-3* (H), *PpcleAmiR4-7* (I), and *Pprpk2* (J), mutant plants supporting inferences from images shown in (D).

Previous studies in *Arabidopsis* have shown that application of CLV3-like, but not TDIF-like, CLEs to roots can arrest meristem function [27]. To assay conservation in peptide function, we germinated *Arabidopsis* seeds on Murashige and Skoog (MS) medium plates containing solute or peptides at a 1 μM concentration. Although solute controls, a randomized peptide, and CLE41 caused no arrest of root development, CLV3 and all of the *Physcomitrella* CLEs caused a significant reduction in root length in *Arabidopsis* resulting from collapse of the root meristem (Figures 7A–7C, 7E, and 7F). *Physcomitrella* CLEs therefore regulate growth and proliferation in a similar manner to CLV3 in *Arabidopsis*. To confirm that PpCLEs can act through a conserved receptor machinery, we used peptide treatment assays on *Arabidopsis* and *Physcomitrella rpk2* mutants (Figure 7). Whereas treatment of WT *Arabidopsis* plants with CLV3-like peptides strongly inhibited root growth, *rpk2* mutants showed less growth inhibition when treated with *Arabidopsis* and *Physcomitrella* peptides (Figures 7A–7C, 7E, and 7F). These data are in line with previously published results showing that RPK2 acts among other receptors to contribute to CLV signaling in *Arabidopsis* [17] and show that *Physcomitrella* CLEs can also act via RPK2 in *Arabidopsis*. To determine whether *Physcomitrella* CLEs act via PpRPK2, we performed similar experiments in WT, *Ppcle*, and *Pprpk2* mutant backgrounds. *Ppcle* mutant gametophores are roughly the same size as *Pprpk2* mutant gametophores, and we reasoned that, if PpCLEs act via PpRPK2, we should detect a response in *Ppcle* mutants, but not *Pprpk2* mutants. As in previous experiments, we found strong inhibition of gametophore development in WT plants (Figure 7D). Potentially due to lack of positional information, treatment of *Ppcle* mutants with CLE peptides did not rescue developmental defects but nevertheless induced a gametophore dwarfing response, consistent with an intact receptor machinery (Figures 7D and 7G–7I). In contrast, *Pprpk2* mutants showed no morphological response to CLE application, suggesting that PpCLEs act via PpRPK2 in regulating 3D growth (Figures 7D and 7G–7J).

## Discussion

### How Might CLV Pattern Cell Division Plane Orientation?

We propose that the CLV pathway regulates the 2D to 3D developmental transition in *Physcomitrella* by orienting gametophore cell division planes and regulating growth and fate. How ligands and receptors act together to do this is not yet clear. One possibility is that CLE ligands diffuse to create a concentration gradient that division planes are patterned against. A similar mechanism involving CLEs patterns cambial meristems in *Arabidopsis* [28], where CLE41 is synthesized in the phloem and diffuses to bind PXY receptors in neighboring procambial cells, thereby imparting spatial information for periclinal division [28]. Constitutive or ectopic expression of *CLE41* disrupts this positional information, resulting in disordered cambial division planes [28]. In *Physcomitrella*, similar patterning could be achieved by sub-cellular localization of receptors to create a graded CLV response in bud initials, or at later stages of development, patterning could be provided by receptor expression in different portions of buds.

It is also possible that CLV signaling does not directly modulate cell division planes but that CLV influences cell division planes via hormone signaling, cell geometry, and/or cell mechanics. Auxin signaling and the activity of microtubule-interacting proteins, such as CLIP-associated proteins (CLASPs), are known to specify cell division planes in *Arabidopsis* embryos [29], and auxin signaling modulates the activity of previously identified factors necessary for correct division plane orientation in *Physcomitrella* buds, including *DEK1* and *NOG1* [30, 31]. There appears to be a complex interplay between auxin and cytokinin in *Physcomitrella* [32, 33, 34], and several phenotypes suggest that this interplay is disrupted in *Ppcle*, *Ppclv*, and *Pprpk2* mutants. For instance, cell fate and proliferation at the gametophore base are perturbed (Figures 4 and 5) and leaf cell proliferation is perturbed in plants treated with CLEs (Figure 6), and these aspects of development are auxin and cytokinin regulated [33, 34]. Linking CLV signaling to the hormone pathways regulating growth and fate will be important in unravelling mechanisms of cell division plane specification during 3D growth.

### CLAVATA-Regulated Stem Cell Function Is an Ancestral Feature of Land Plants

The data we present are important in two evolutionary contexts. First, they show that the CLV pathway originated with land plants and that CLV-regulated stem cell proliferation and function is likely to be an ancestral feature of land plants. The acquired capacity of land plants to orient stem cell divisions in multiple planes enabled diversification by permitting plants to develop upright axes with organs arranged in multiple orientations, a crucial step in shoot evolution [1]. Stem cell division plane defects in *Ppcle* mutants specifically affect the transition to 3D growth and the 3D growth phase, and morphological responses to peptide application are also specific to the 3D growth phase. Thus, in an ancient land plant group, CLV regulates a developmental transition that mirrors an evolutionary transition. The data suggest that CLV was a genetic novelty for a key morphological innovation of land plants.

### CLAVATA-Regulated Meristem Functions Originated prior to WOX- and KNOX-Regulated Meristem Functions

Second, the data are important in the context of evolving gene regulatory networks for land plant meristem function. Whereas the first land plant meristems comprised a single gametophytic stem cell, the multicellular sporophyte meristems of vascular plants combine stem cell and more generally proliferative capacities [1]. Class I *KNOX* genes regulate meristematic proliferation in vascular plants [35, 36], but these roles are not shared between bryophytes and vascular plants. Moss *KNOX* (*MKN*) genes are primarily expressed in sporophyte tissues [24, 37], and although loss-of-function *mkn2* mutants have elongation defects in sporophytes, they have normal gametophytes [37]. *WOX* genes are key regulators of stem cell proliferation in *Arabidopsis* [19]. However, this function was acquired by the recently derived *WUS* gene clade [38, 39], and the downstream pathways regulated by CLV in *Physcomitrella* are likely to be distinct from those in *Arabidopsis* as *Ppwox13L* mutant gametophores develop normally [40]. Thus, class I *KNOX*- and *WOX*-regulated meristem functions were both acquired after the bryophyte-vascular plant divergence. CLV was important in the origin of land plant meristem functions in the gametophyte stage of the life cycle, and we speculate that CLV was recruited to regulate stem cell function in the sporophyte stage of the life cycle prior to the origin of *KNOX*- and *WOX-* regulated meristem functions.

## STAR★Methods

### Key Resources Table

**Table:** 

REAGENT or RESOURCE	SOURCE	IDENTIFIER
**Bacterial and Virus Strains**		

*E. coli* strain DH5α	Widely distributed	N/A
*E. coli* strain DB3.1	Widely distributed	N/A
*E. coli* strain DH10B	Widely distributed	N/A

**Chemicals, Peptides, and Recombinant Proteins**		

Taq polymerase	Widely available	N/A
Phusion High-Fidelity DNA polymerase	ThermoFisher	Cat#F530S
Novagen KOD Hot Start polymerase	Sigma-Aldrich	Cat#71086
Superscript II reverse transcriptase	ThermoFisher	Cat#18064022
Restriction enzymes for cloning	New England Biolabs	N/A
DNase	Fermentas	Cat#EN0525
MS medium	Melford	Cat#M0221
Plant agar	Duchefa	Cat#P1001
Driselase (basidiomycetes sp.)	Sigma-Aldrich	Cat#8037
Polyethylene glycol (PEG) 6000	Sigma-Aldrich	Cat#81255
G418 disulphate	Melford	Cat#G0175
Hygromycin B	Melford	Cat#H7502
Blasticidin S	Melford	Cat#B1220
α-32P dCTP	GE Healthcare	Cat#PB10205
X-GlcA	Melford	Cat#MB1021
Propidium iodide	Sigma-Aldrich	Cat#P4864
Synthetic CLE peptides (95% purity)	Genecust	N/A
Lugol’s stain	Fisher Scientific	Cat#12801823

**Critical Commercial Assays**		

RNeasy RNA extraction kit	QIAGEN	Cat#74104
Plasmid Plus Midi kit	QIAGEN	Cat#12943
Amersham Rediprime II DNA labeling kit	GE Healthcare	Cat#RPN1633
Dig-High Prime DNA labeling and detection starter kit II	Sigma-Aldrich	Cat#11585614910
Dig Easy Hyb	Sigma-Aldrich	Cat#11585762001

**Experimental Models: Organisms/Strains**		

*Physcomitrella patens* Gransden	Widely available	N/A
*PpCLE1::NGG* line	This study	N/A
*PpCLE2::NGG* line	This study	N/A
*PpCLE7::NGG* line	This study	N/A
*PpCLV1a::NGG* line	This study	N/A
*PpCLV1b::NGG* line	This study	N/A
*PpRPK2::NGG* line	This study	N/A
*PpcleamiR1-3* mutant	This study	N/A
*PpcleamiR4-7* mutant	This study	N/A
*Ppclv1a* mutant	This study	N/A
*Ppclv1b* mutant	This study	N/A
*Ppclv1ab* double mutant	This study	N/A
*Pprpk2* mutant	This study	N/A
*Arabidopsis thaliana* Col-0	Widely available	N/A
*Arabidopsis thaliana rpk2-4* mutant	[17]	N/A
*Arabidopsis thaliana clv1,bam1,bam2,bam3* mutant	[25]	N/A

**Oligonucleotides**		

A list of oligonucleotides is given in Table S4	N/A	N/A

**Recombinant DNA**		

*PIG1NGGII* construct	[41]	N/A
*PpCLE1::NGG* construct	This study	N/A
*PpCLE2::NGG* construct (NptII)	This study	GenBank: MH310732, MH310732
*PpCLE7::NGG* construct	This study	N/A
*PpCLV1a::NGG* construct	This study	N/A
*PpCLV1b::NGG* construct	This study	N/A
*PpRPK2::NGG* construct (AphIV)	This study	GenBank: MH310733
*pRS300*	[42]	N/A
pGREEN (Hyg)	[43]	N/A
pGREEN (Kan)	[43]	N/A
pBJ36	[44]	N/A
pBRACT211	[45]	N/A
pJH125	This study	N/A
pJH131	This study	N/A
*PpcleAmiR1-3* construct	This study	GenBank: MH310734
*PpcleamiR4-7* construct	This study	GenBank: MH310735
*U3::Ppclv1a* sgRNA5 construct	This study	GenBank: MH310736
*U3::Ppclv1a* sgRNA7 construct	This study	GenBank: MH310737
*U6::Ppclv1b* sgRNA construct	This study	GenBank: MH310738
*pACT::Cas9* construct	[46]	N/A
pNRF	[47]	N/A
pBHRF108	[48]	N/A
pDONR2.1	Invitrogen	N/A
pGEMT-EASY	Promega	Cat#A1360

**Software and Algorithms**		

tBLASTn	[49]	N/A
SignalP	[50]	v4.0
MEGA	[51]	v7.0.26
Figtree	http://tree.bio.ed.ac.uk/software/figtree/	v1.4.3
AmiR design software	http://wmd3.weigelworld.org/cgi-bin/webapp.cgi	N/A
CRISPR design software	[52]	http://crispor.tefor.net/
ImageJ	http://imagej.net/Welcome	V1.4.8
Adobe Photoshop	Adobe	N/A
Adobe Illustrator	Adobe	N/A

### Contact for Reagent and Resource Sharing

Further information and requests for resources and reagents should be directed to and will be fulfilled by the Lead Contact, Jill Harrison (jill.harrison@bristol.ac.uk). Please note that the transfer of transgenic materials will be subject to MTA and any relevant import permits.

### Experimental Models and Subject Details

#### *Arabidopsis* plant growth

Columbia (Col-0), *rpk2-4* (*cli*) or *clv1*/*bam1*/*bam2*/*bam3* mutants [17, 25] were used for *Arabidopsis* experiments. Homozygous *rpk2-4* mutants were confirmed using a BamHI dCAPs screen with a PCR fragment amplified using primers AtRPK2-BamHIF and AtRPK2-BamHIR (see primer list). Seeds were surface sterilized in 5% (v/v) sodium hypochlorite for 10 min and washed three times with sterile de-ionised water. They were then stratified at 4°C in darkness for 48 hr and sown on 0.5 X MS plates containing 0.8% agar [53]. Plants were grown vertically for 7 days at 25°C in a 16 hr light/ 8 hr dark cycle prior to observation (*rpk2* experiments) or at 22°C under continuous light (*clv1*/*bam1*/*bam2*/*bam3* experiments).

#### *Physcomitrella* plant growth

The Gransden strain of *Physcomitrella patens* [54] was used for all experiments. Plants were grown in sterile culture on BCDAT plates at 23°C in continuous light at 30-50 μmols^-1^ in Sanyo MLR-351 growth cabinets. BCDAT medium comprises 250mg/L MgSO_4_.7H_2_O, 250mg/L KH_2_PO_4_ (pH6.5), 1010mg/L KNO_3_, 12.5mg/L, FeSO_4_.7H_2_O, 0.001% Trace Element Solution (0.614mg/L H_3_BO_3_, 0.055mg/L AlK(SO_4_)2.12H_2_O, 0.055mg/L CuSO_4_.5H_2_O, 0.028mg/L KBr, 0.028mg/L LiCl, 0.389mg/L MnCl_2_.4H_2_O, 0.055mg/L CoCl_2_.6H_2_O, 0.055mg/L ZnSO_4_.7H_2_O, 0.028mg/L KI and 0.028mg/L SnCl_2_.2H_2_O), 0.92 g/L C_4_H_12_N_2_O_6_ and 8g/L agar with CaCl_2_ added to a 1mM concentration after autoclaving. Protonemal cultures for transformation were grown on BCDAT plates overlaid with autoclaved cellophane disks and molecular and phenotypic analyses were undertaken using 1 mm spot cultures unless otherwise stated.

### Method Details

#### Sequence retrieval

##### *CLE* genes

Previously described *Arabidopsis thaliana* and *Oryza sativa CLE* sequences were respectively retrieved from TAIR and RAP-DB [55]. *Selaginella moelendorffii* [56], *Glycine max* [57, 58] and *Picea abies* [59] *CLE*s were retrieved from NCBI. To extend taxon sampling within land plants and identify previously unknown *CLE*s, the CLE domains of *Arabidopsis thaliana* CLV3 and CLE41 were used as tBLASTn queries with an e-value cutoff of e^-100^ to screen transcriptome or draft genome assemblies of a basal angiosperm (*Amborella trichopoda*), a fern (*Diplazium wichurae*), a hornwort (*Anthoceros agrestis*), a moss (*Physcomitrella patens* v1.6 [60]) and a liverwort (*Marchantia polymorpha*). Positive hits were used in reciprocal BLASTs until no new sequences were retrieved. All sequences retrieved were checked for the presence of a signal peptide [61] using SignalP [50, 62]. Newly identified CLE sequences were named with a two-letter prefix denoting the genus and species and numbered (Table S1). A recent cluster analysis [63] succeeded our analyses with slight variation in CLE numbers between species for reasons explained in [63]. An updated version of the *Physcomitrella* genome (v 3.1 [64]) also succeeded our analyses, and this includes two further *PpCLE*s that encode the same CLE motif as PpCLEs 1, 2 and 3. While these were not included in this study, V3 gene IDs including *PpCLE8* and *PpCLE9* are listed in Table S3. Transcriptomes and draft or complete genomes of charophyte (*Coleochaete nitellarum, Spirogyra* sp., *Chara braunii*) and chlorophyte algae (*Ulva linza, Chlamydomonas reinhardtii*, *Volvox carteri*, *Ostreoscoccus tauri* and *Chlorella vulgaris*) were also searched but no *CLE*s were found. A previously annotated *Chlamydomonas reinhardtii CLE* [65] was re-analyzed and discarded due to lack of similarity to other *CLE*s and a premature in-frame stop codon. A full list of taxa and databases searched is given in Table S2.

##### *CLV1/RPK2* genes

*Arabidopsis* CLV1 and RPK2 sequences were used to query the databases listed above using tBLASTn searches with an e-value cut-off of e^-1000^. As the LRR-Receptor kinase family is large, only sequences that retrieved *CLV1* or *RPK2* as a top hit in reciprocal BLASTs to *Arabidopsis* were used in further analyses. Newly identified *CLV1*-like and *RPK2*-like genes were named with a two-letter prefix denoting the genus and species and given an alphabetical epithet (Table S1). A list of taxa searched is given in Table S2.

#### Phylogenetic reconstruction

To infer CLE relationships, the conserved 12 amino acid CLE motif from 193 *CLE*s was used in neighbor joining reconstructions compiled with the JTT model in MEGA7.0.26 [51] (Figure S1; Data S1). This approach was taken because there is little conservation in CLE structure outside the CLE motif and so few characters can only yield limited phylogenetic signal (see [63]). To infer CLV/BAM relationships, 525 conserved amino acid residues from 36 genes were used in maximum likelihood reconstructions with the JTT model in MEGA7.0.26 [51] (Figure S2; Data S2). To infer RPK2 relationships, 782 conserved amino acid residues from 18 genes were used in maximum likelihood reconstructions with the JTT model in MEGA7.0.26 [51] (Figure S3; Data S3). For all analyses 100 bootstrap replicates were performed and support values over 50% (CLE tree) or 70% (CLV1/BAM and RPK2 trees) are represented above the branches.

#### Molecular biology

##### RT-PCR

Total RNA was isolated from 4 day-old protonemal cultures and 10, 21 or 28 day old spot cultures using the QIAGEN RNeasy method. RNA was DNase treated prior to reverse transcription with SuperScript II following manufacturer’s guidelines. Semiquantitative RT-PCR was undertaken using *UBIQUITIN* (Pp1s56_52V6.1) as a loading control. Where possible, primers were designed to span introns to detect genomic contamination, and sequences are listed in Table S4.

##### Genomic DNA extraction

Genomic DNA was extracted from protonemal cultures using a CTAB (Hexadecyltrimethylammonium bromide) protocol. Snap-frozen tissue was ground in liquid nitrogen and transferred to tubes containing prewarmed extraction buffer (2% CTAB, 1.4 M NaCl, 100 mM Tris pH8.0, 20 mM EDTA pH8.0, 2% PVP and 1 mg/mL RNaseA), with no more than 100 mg of tissue per mL of buffer. Samples were incubated for 10 min at 65°C and an equal volume of 24:1 chloroform:isoamyl alcohol was added and mixed with each sample to form an emulsion. The tubes were centrifuged at high speed (> 10,000 rpm) for 10 min, and the aqueous phase was transferred to a fresh tube prior to DNA precipitation with an equal volume of isopropanol and repeated centrifugation. DNA was washed with 70% ethanol and dissolved in water, 10 mM Tris pH 8.0 or 10 mM Tris pH 8.0 with 1 mM Na_2_EDTA.

##### Generation of promoter::NGG constructs

Promoter sequences from *PpCLE1* (2.1 kbp), *PpCLE2* (2.1 kbp), *PpCLE7* (2 kbp), *PpCLV1a* (2 kbp), *PpCLV1b* (2.8 kbp) and *PpRPK2* (1.4 kbp) were PCR amplified using a proof-reading Taq polymerase and primers listed in Table S4 and cloned directly or via pGEMT Easy into the SmaI site of PIG1NGGII [41] or derivatives with alternative selection cassettes and sequenced prior to linearization and transformation as illustrated in Methods S1.

##### Generation of AmiR constructs

To generate *PplcleAmiR1-3* and *PplcleAmiR4-7* constructs, resistance cassettes from pGREEN [45] were first inserted into a blunt-ended HindII site of pBJ36 [44]. A soybean *UBIQUITIN* promoter from pBRACT211 [45] was inserted into the SmaI site to drive AmiRNA expression and the resultant plasmids were named pJH125 (KanR) and pJH131 (HygR). AmiRNAs were designed according to [42], generated by degenerate PCR using a proof-reading Taq polymerase and the pRS300 plasmid as a template, cloned into pGEMT-EASY and transferred as XmaI/BamHI fragments into pJH125 or pJH131. Silencing constructs were checked by sequencing and digested with SacI for transformation as illustrated in Methods S1.

##### Generation of CRISPR constructs

Small cassettes containing two BsaI restriction sites and sgRNAs [66] driven by the *Physcomitrella* U3 or U6 promoter and flanked by *attB* sites were synthesized and cloned into pDONR201. sgRNA sequences were selected and screened for off target hits in the *Physcomitrella* V3 genome using http://crispor.tefor.net/. To clone guide RNAs into expression cassettes, two primers consisting of guide sequences with overhangs for U3 and U6 promoters were annealed and ligated into U3 or U6 expression vectors pre-digested with BsaI. Constructs were checked by sequencing and co-transformed with *pACT::Cas9* [46] to engineer mutants as illustrated in Methods S1.

##### Generation of RPK2 KO construct

5′ and 3′ flanking regions were PCR amplified with a proof-reading Taq polymerase and cloned sequentially into pGEMT-EASY using primers listed in Table S4. The resultant plasmid was digested with PmeI and AscI, and the AphIV cassette from pBHRF-108 [48] was ligated between *PpRPK2* flanking regions. This plasmid was checked by sequencing and linearized for transformation as illustrated in Methods S1.

#### Transgenic line generation and phenotype analyses

##### Moss transformation and line authentication

For gene targeting and AmiR approaches, 10-20 μg of plasmid DNA was isolated using the QIAGEN Plasmid Plus Midi system and linearized as illustrated in Methods S1. For CRISPR approaches, 5-7 μg of Cas9 and pNRF, and 2-3 μg of each gRNA-expressing construct were purified and pooled for transformation [46] at a concentration of at least 1 μg per μL. All solutions for the transformation procedure were prepared prior to commencing transformation [67]. First, a polyethylene glycol (PEG) solution was prepared by adding 10 mL of mannitol/CaNO_3_ solution (8% mannitol, 0.1 M Ca(NO_3_), 10 mM Tris pH7.2) to 2 g of molten PEG 6000, and the tube containing the solution was left in a water bath at 45°C. To isolate protoplasts, homogoenous protonemal cultures were grown for 5 days to a week post passage. A 1% driselase solution was prepared in 25 mL 8% mannitol, and the supernatant was removed and filter sterilized into to a clean 50 mL falcon tube following centrifugation. Tissue from 4-6 plates was transferred into the driselase solution and the tissue suspension was left for 30-40 min with intermittent mixing to allow cell wall digestion. The mixture was then transferred into a fresh tube through a 50 μm filter to remove cell and cell wall debris. Protoplasts were sedimented by centrifugation for 3 min at 120 g, resuspended and washed three times in 10 mL of 8.0% mannitol prior to counting with a hematocytometer. Protoplasts were then sedimented and resuspended to a density of 1.2 × 10^6^ per mL in MMM solution (0.5 M mannitol, 0.15 M MgCl_2_ and 0.1% MES pH5.6). 300 μL aliquots of protoplasts were dispensed into falcon tubes prior to addition of DNA and 300 μL PEG solution, and cells were then heat shocked for 5 min at 45°C. Transformation mixtures were progressively diluted with 1 mL of 8% mannitol solution and washed. Protoplasts were then sedimented by centrifugation as above and washed four more times. After the final wash and spin, protoplasts were resuspended in 5 mL liquid BCD medium (constituents as specified above but without ammonium tartrate or agar) with 8% mannitol, 10 mM CaCl_2_ and 0.5% glucose, wrapped in aluminum foil and left at 23°C overnight. The next day, the protoplast suspension was plated onto BCDAT plates overlain with cellophane and containing 8% mannitol and 5 g/L glucose, using c.1 mL per plate. Plants were grown under standard conditions until regenerants comprised 10-20 cells. Cellophane discs were then transferred onto BCDAT plates containing antibiotics for selection (25 μg/mL Hyg, 50 μg/mL G418, 100 μg/mL BSD). Plants were grown for 2 weeks on selection plates prior to transfer onto BCDAT plates lacking antibiotic for 2 weeks and then back on to selection plates for a further 2 weeks. All lines were screened by PCR, RT-PCR, Southern analysis or sequencing as illustrated in Methods S1. PCR conditions were standard and primer sequences are listed in Table S4.

##### Southern hybridization

For *PpcleAmiR* Southerns, 10-15 μg genomic DNA was digested with EcoRV and fractionated in 0.8% agarose by gel electrophoresis. DNA in each gel was depurinated with 0.2 M HCl for 20 min and denatured with 0.4 M NaOH for 20 min prior to neutralization for 20 min in a solution containing 3 M NaCl and 1 M Tris pH 7.5. Gels were inverted onto a Whatman paper wick inserted into a bath of 20 X SSC solution, and DNA was transferred onto a nitrocellulose membrane by overnight Southern blotting. DNA was UV crosslinked to the membrane and the membrane was rinsed in water prior to immersion in pre-hybridization solution (3 X SSC, 1% SDS, 0.1% sodium pyrophosphate, 5 X Denhardt’s and 200 μg per mL sheared salmon sperm DNA). The probe template was excised with EcoRV and BamHI from the *PpcleAmiR1-3* construct and the probe was synthesized using an Amersham Rediprime II DNA labeling kit as per manufacturer’s instructions. Hybridization was undertaken in a 3 X SSC buffer at 58°C and this was followed by two 20 min washes at 58°C in 3 X SSC and 2 X SSC buffers respectively. Membranes were wrapped in Saran Wrap and used to expose X-ray film, and film was then developed using a film processor. For *promoter::NGG* and *Pprpk2* Southerns, 2.5-3 μg genomic DNA was digested as illustrated in Methods S1. Probe templates comprising *PIG1* flanking sequence, *PpRPK2* coding sequence or a hygromycin resistance cassette were PCR amplified and labeled using the Roche DIG High Prime system. Hybridization was undertaken overnight at 42°C using the Roche DIG Easy Hyb system. Washing and detection were performed using the manufacturer’s protocol from the Roche DIG High Prime DNA labeling and Detection Starter kit II.

##### *Physcomitrella* plant imaging

To assess whole plant and gametophore phenotypes, 4 to 5 week-old spot cultures were imaged using a Keyence VHX-1000E digital microscope with a 20-50 X or 50-200 X objective. To analyze bud phenotypes, confocal imaging was undertaken on tissue stained with 0.5 mg/ml propidium iodide using a Leica TCS SP5 microscope with excitation from the 488 or 514 laser line and emission collected at 600-650 nm or using a Zeiss 710 LSM with excitation from a 514 laser line and emission collected at 566-650 nm. To analyze leaf phenotypes, leaves were removed form gametophores, arranged in heteroblastic series, cleared in 1% chloral hydrate overnight, washed in deionised water and treated with 2M NaOH for 2 hr. They were then washed with water and stained with 0.05% toluidine blue for 2 min before destaining for 10 min in water. The stained leaves were then mounted on a slide under a coverslip and imaged to visualize cell outlines. Adobe Illustrator was used to trace leaf outlines to produce silhouettes for illustration purposes (Figure 6). Quantitative analyses of leaf size were performed using ImageJ, and cell numbers were evaluated using the ‘analyze particles’ option [68]. Leaf size comparisons were undertaken using leaves from the same point in the heteroblastic leaf series [33] as stipulated in figure legends.

##### *Arabidopsis* plant imaging

Root length was scored from scanned images of plants grown on ½ X MS plates using ImageJ [68]. To visualize *rpk2* meristems, roots were stained with Lugol’s stain, cleared, and imaged using a 20 X objective on a Leica DMRXA microscope with DIC [69]. *clv1*/*bam1*/*bam2*/*bam3* roots were stained with 15 mM propidium iodide and imaged using a C-Apochromat 40 X/1.20 W Korr objective on a Zeiss LSM710 microscope. Excitation and emission windows for propidium iodide were 560 nm and 566-719 nm respectively. Confocal images were analyzed and processed using ImageJ and Adobe Photoshop.

##### GUS staining and imaging

*Physcomitrella* plants grown on BCDAT were cut out of plates with agar and incubated at 37°C in a 100 mM phosphate buffer with 10 mM Tris pH8.0, 1 mM EDTA pH8.0, 0.05% Triton X-100, 1 mg/mL X-GlcA (5-Bromo-4-chloro-3-indolyl-β-D-glucuronic acid) and potassium ferri/ferrocyanide using concentrations and times indicated in Figure 2 and legend. Plants were bleached in 70% ethanol and dissected and mounted in 0.3% low melting point agarose prior to imaging with a Keyence VHX-1000 digital microscope with a 0-50 X or a 50-200 X objective.

##### CLE peptide application

Synthetic CLE peptides (Genecust, >95% purity) were dissolved in phosphate buffer (50 μm, pH6.8) to stock concentrations of 1 mM and 10 mM. Plants were grown on BCDAT plates containing peptides diluted to concentrations specified in the main text.

### Quantification and Statistical Analysis

Quantification and statistical analyses were undertaken as stipulated in main text and SI figures and figure legends.

### Data and Software Availability

Genome and transcriptome data were searched as described in Method Details and details of data repositories are listed in Table S2.

## Supplemental Information

Document S1. Figures S1–S7 and Tables S1–S4

Data S1. CLE Sequence Alignment, Related to Figure S1

Data S2. CLV Sequence Alignment, Related to Figure S2

Data S3. RPK2 Sequence Alignment, Related to Figure S3

Methods S1. Additional Data Outlining Strategies for Generating promoter::NLSGUSGFP Reporter Lines, PpcleAmiR Mutants, Ppclv1 CRISPR/Cas9 Mutants, and Pprpk2 KO Mutants, Related to STAR Methods

Document S2. Article plus Supplemental Information
