# Supplementary material for: CLAVATA Was a Genetic Novelty for the Morphological Innovation of 3D Growth in Land Plants
Source: Curr Biol. 2018 Aug 6;28(15):2365–2376.e5. doi: 10.1016/j.cub.2018.05.068 (PMC6089843; doi:10.1016/j.cub.2018.05.068)
Supplement: Document S1. Figures S1–S7 and Tables S1–S4 [file mmc1.pdf]

Current Biology, Volume *28*

Supplemental Information

CLAVATA Was a Genetic Novelty for the Morphological Innovation of 3D Growth in Land Plants

Chris D. Whitewoods, Joseph Cammarata, Zoe Nemec Venza, Stephanie Sang, Ashley D. Crook, Tsuyoshi Aoyama, Xiao Y. Wang, Manuel Waller, Yasuko Kamisugi, Andrew C. Cuming, Péter Szövényi, Zachary L. Nimchuk, Adrienne H.K. Roeder, Michael J. Scanlon, and C. Jill Harrison

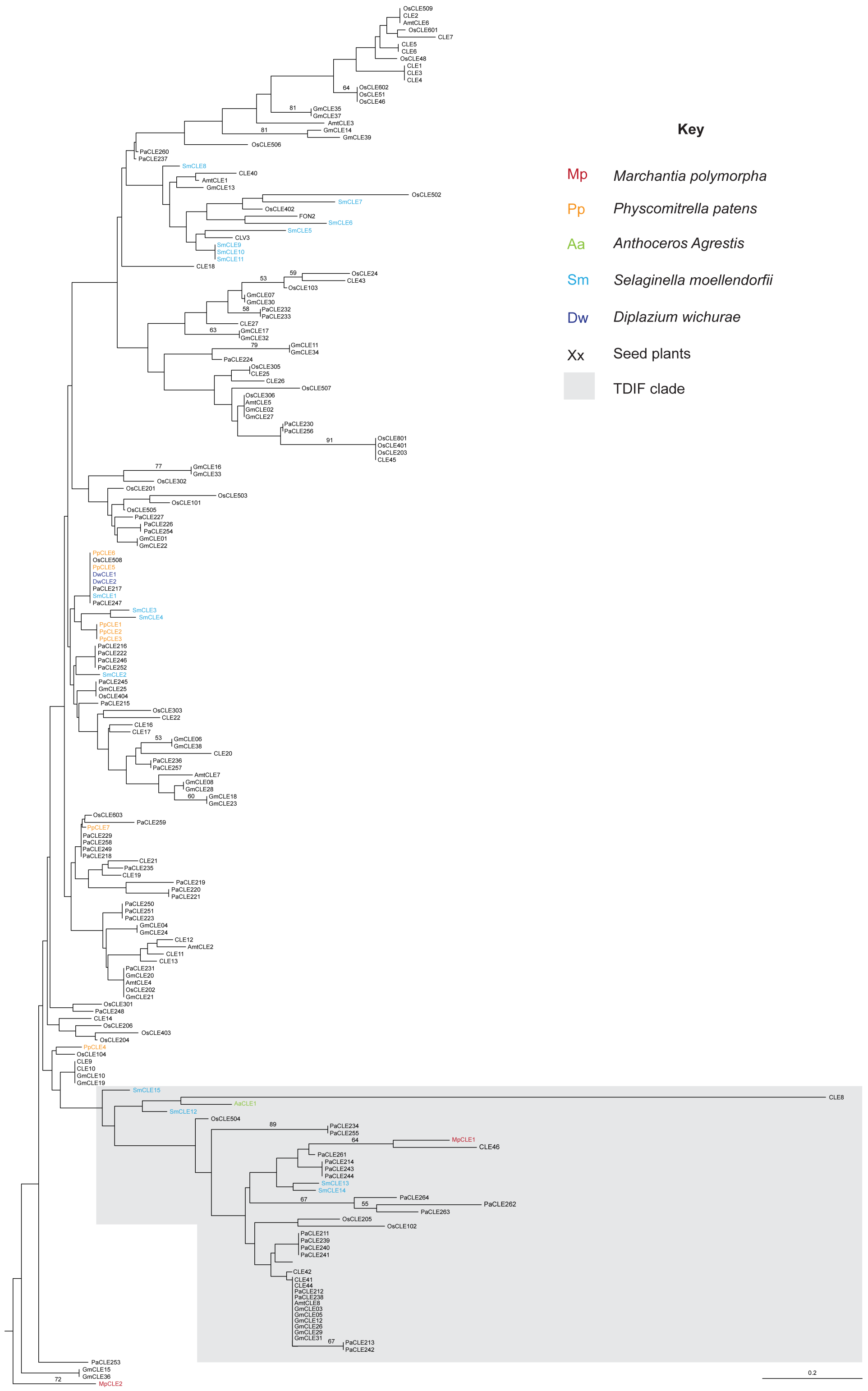

Figure S1: NJ tree showing relationships between land plant CLEs (related to Figure 1). 193 CLE motifs were aligned and a neighbour-joining analysis was undertaken as described in SI Methods. A *Marchantia polymorpha* CLE was used to root the tree, *Physcomitrella patens* was selected to represent mosses, *Anthoceros agrestis* was selected to represent hornworts, *Selaginella moellendorfii* was selected to represent lycophytes, *Diplazium wichurae* was selected to represent monilophytes, and seed plant sequences were retreived from *Picea abies*, *Amborella trichopoda*, *Glycine max*, *Oryza sativa* and *Arabidopsis thaliana*. 100 bootstrap replicates were performed, but bootstrap support was very low as there are few characters, and support values of > 50 are shown.

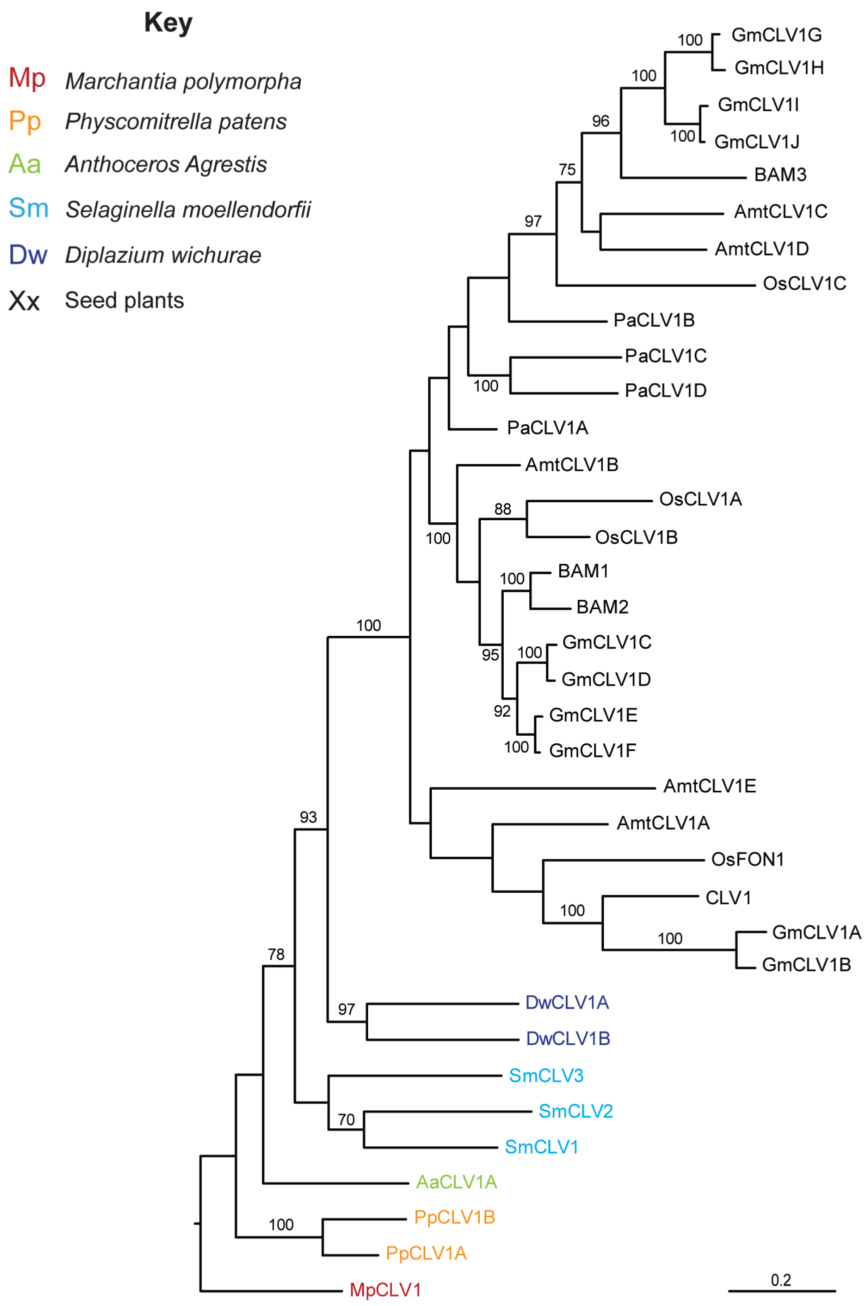

Figure S2: ML tree showing relationships between land plant BAM/CLV1-like proteins (related to Figure 1). 36 BAM/CLV1-like receptor-like kinase sequences were aligned, and data were analysed using the maximum likelihood method with the JTT matrix-based model as described in SI Methods. All positions containing gaps and missing data were removed prior to analysis, leaving a total of 525 in the final dataset. The tree with the highest log likelihood is shown. *Marchantia polymorpha* was sampled to represent liverworts, *Physcomitrella patens* was sampled to represent mosses, *Anthoceros agrestis* was selected to represent hornworts, *Selaginella moellendorfii* was selected to represent lycophytes, *Diplazium wichurae* was selected to represent monilophytes, and seed plant sequences were retreived from *Picea abies*, *Amborella trichopoda*, *Glycine max*, *Oryza sativa* and *Arabidopsis thaliana*. The tree was rooted on MpCLV1 in line with current estimates of land plant phylogeny, and bootstrap values of > 70 from 100 replicates are shown next to branches. Branch lengths represent the number of substitutions per site.

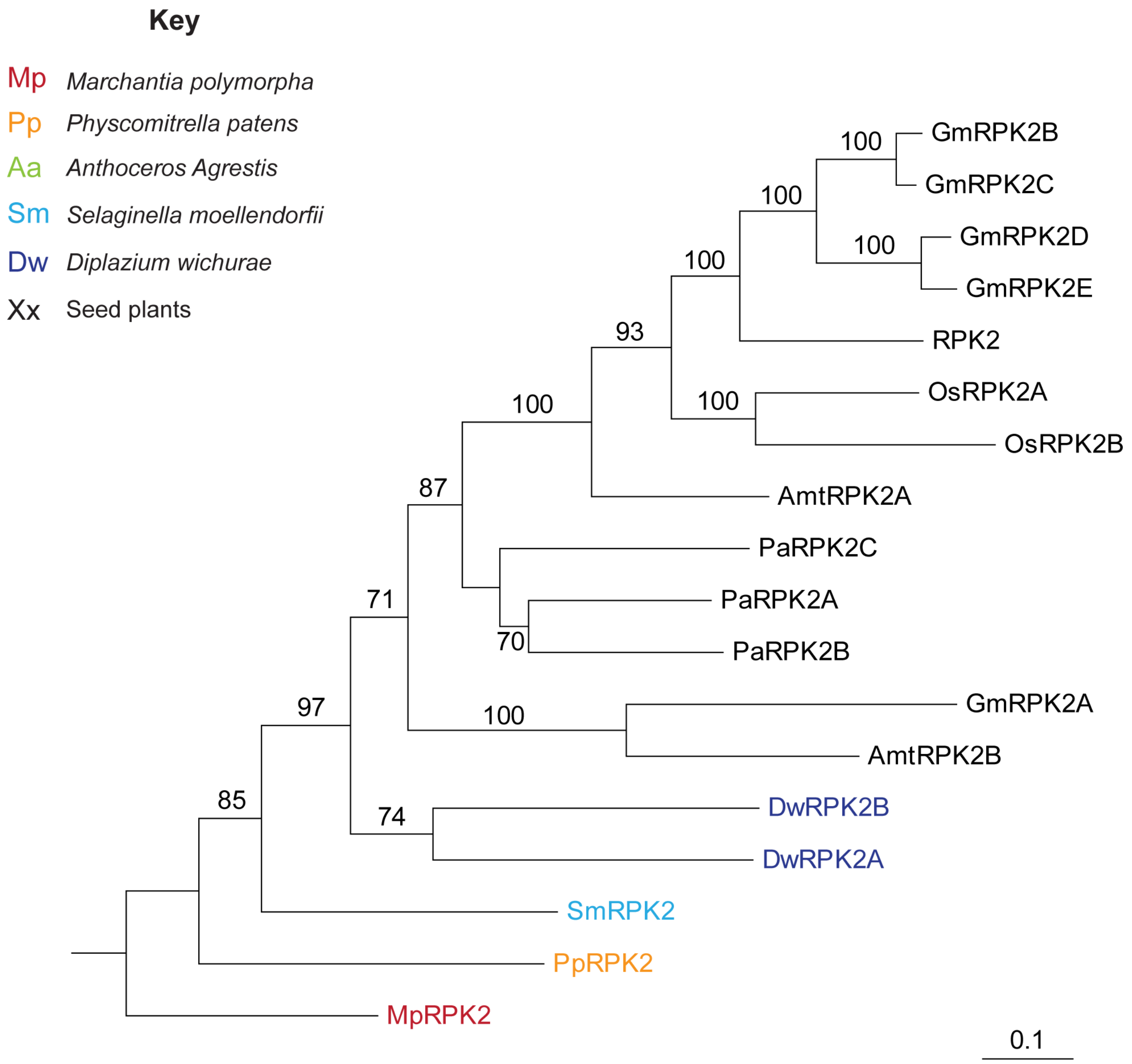

Figure S3: ML tree showing relationships between land plant RPK2-like proteins (related to Figure 1). 18 RPK2-like receptor-like kinase sequences were aligned and the phylogenetic tree was reconstructed using the maximum likelihood method with the JTT matrix-based model as described in SI Methods. All positions containing gaps and missing data were removed prior to analysis, leaving a total of 782 in the final dataset. The tree with the highest log likelihood is shown. *Marchantia polymorpha* was sampled to represent liverworts, *Physcomitrella patens* was sampled to represent mosses, *Anthoceros agrestis* was selected to represent hornworts, *Selaginella moellendorfii* was selected to represent lycophytes, *Diplazium wichurae* was selected to represent monilophytes, and seed plant sequences were retreived from *Picea abies*, *Amborella trichopoda*, *Glycine max*, *Oryza sativa* and *Arabidopsis thaliana*. The tree was rooted on MpRPK2 in line with current estimates of land plant phylogeny, and bootstrap values of > 70 from 100 replicates are shown next to branches. Branch lengths represent the number of substitutions per site.

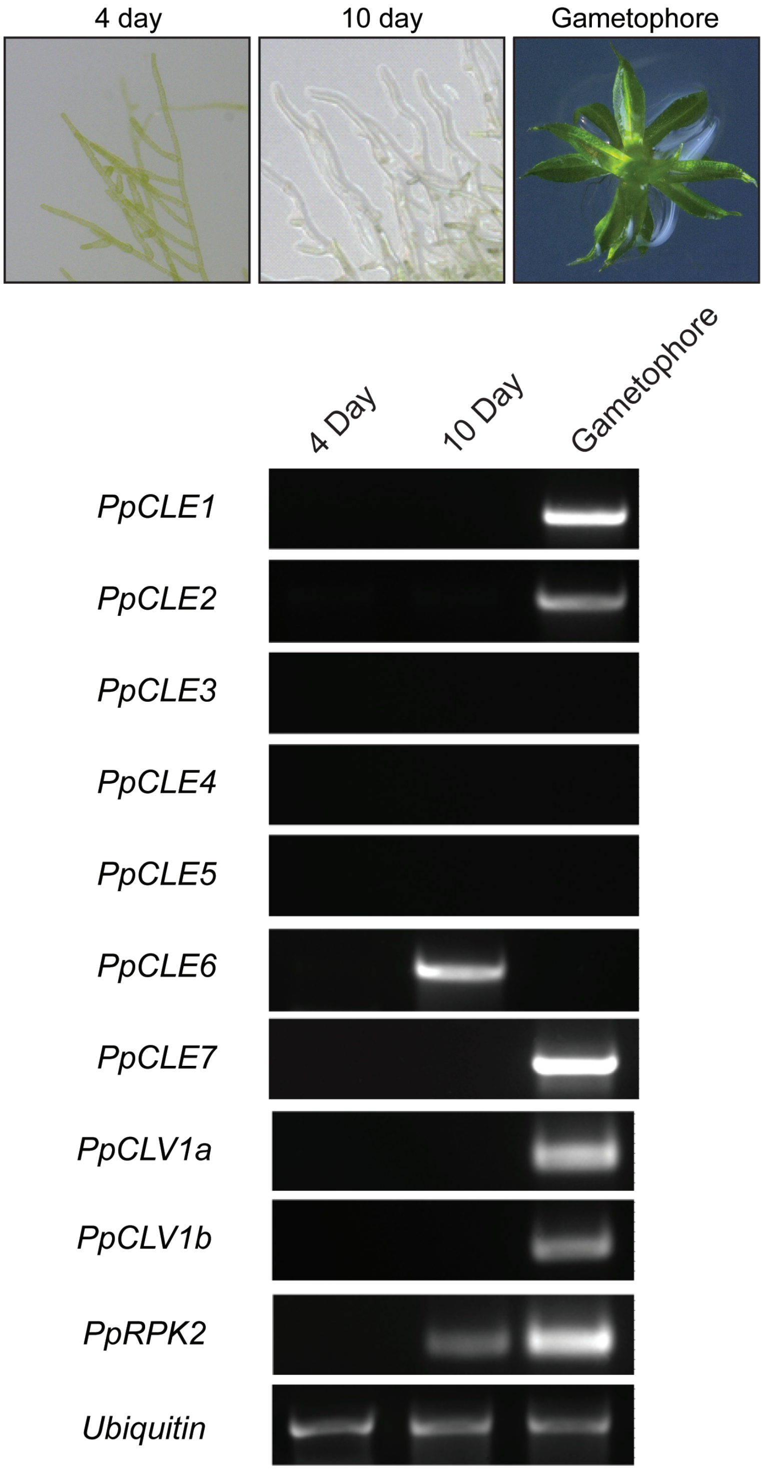

Figure S4: Expression of CLV signalling components in *Physcomitrella* gametophytic tissues as evaluated by RT-PCR (related to Figure 2). *PpCLE*s *1*, *2* and *7* were expressed in gametophores. *PpCLV1a*, *PpCLV1b* and *PpRPK2* were also expressed in gametophores and *PpRPK2* expression was also detected in 10-day old protonemal tissues, which is when gametophores first start to initiate under our growth conditions.

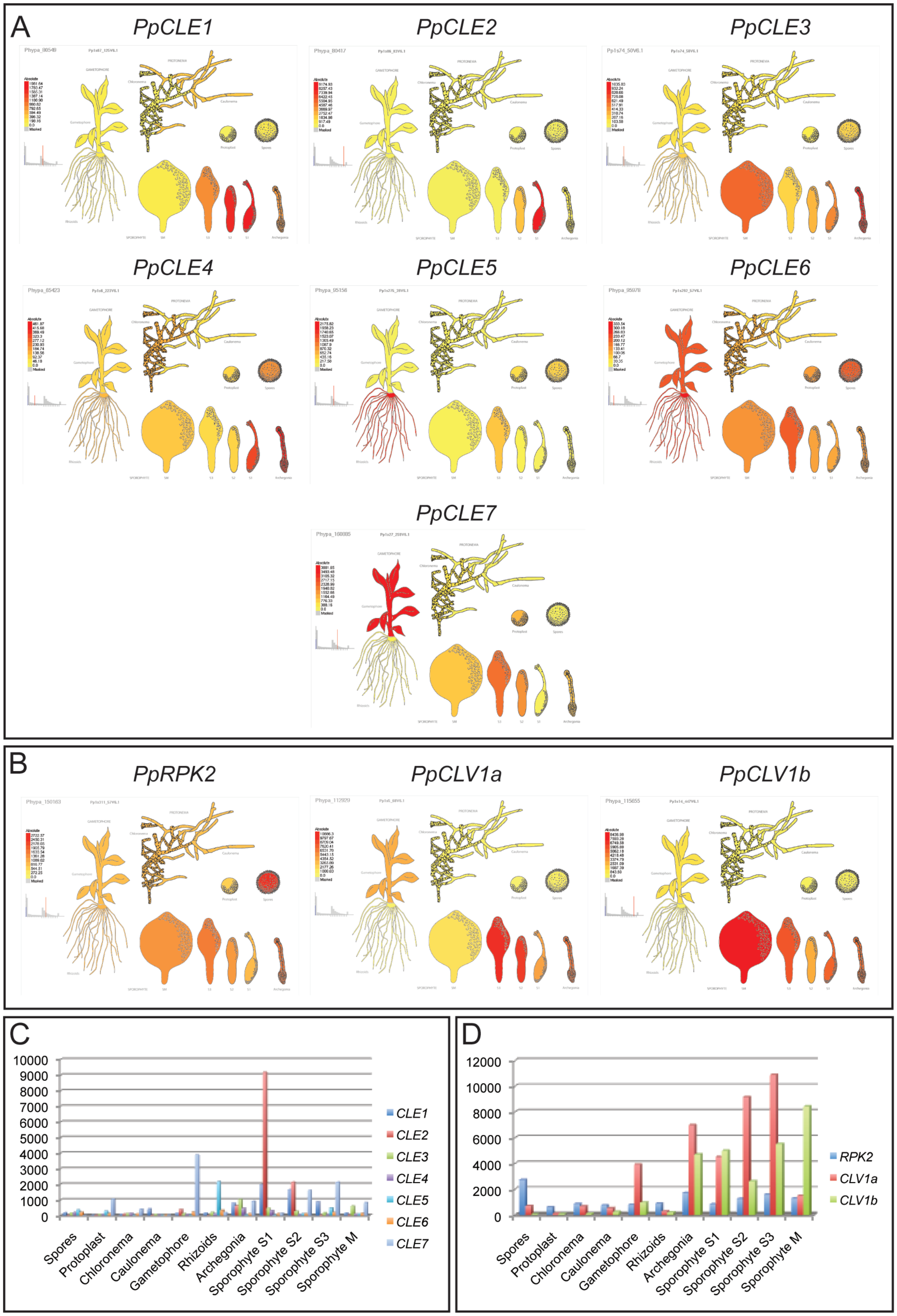

Figure S5: Expression of CLV signalling components in *Physcomitrella* tissues evaluated by eFP Browser data [S1] (related to Figure 2). (A) Expression patterns of *PpCLE*s *1-7*. (B) Expression patterns of receptor components. (C) Quantitative comparison of *PpCLE1-7* expression levels. (D) Quantitative comparison of *PpCLV1a*, *PpCLV1b* and *PpRPK2* expression levels.

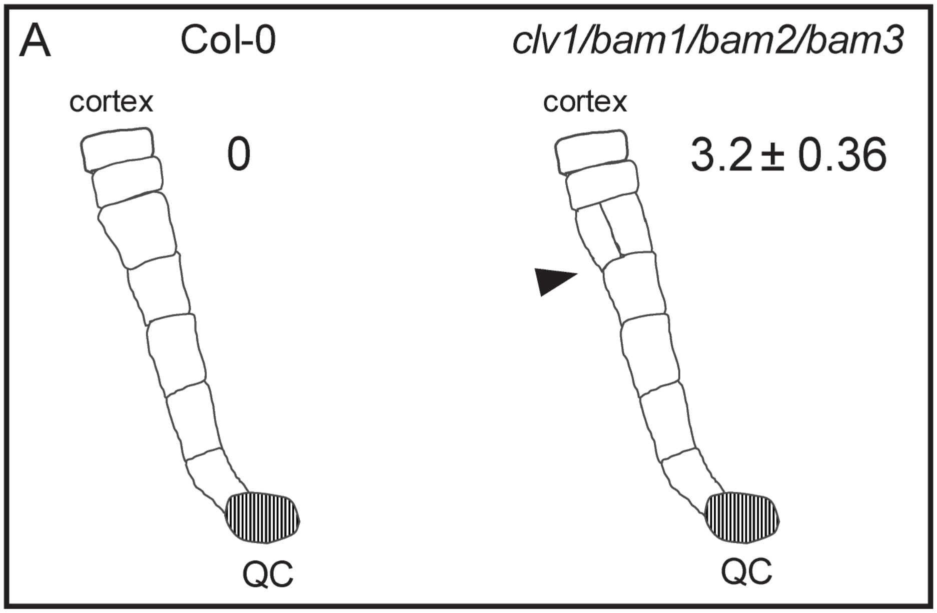

B

Root Length (cm) at time of imaging (stage-matched)		
**Col-0**		***clv1/bam1/bam2/bam3***
1.375	*Mean*	1.499
0.344	*SD*	0.33
0.661	*Min*	1.006
1.999	*Max*	2.165
19	*N*	13
0.078919012	*SE*	0.091525532

C

**Col-0**			***clv1/bam1/bam2/bam3***		
Label	*Meristematic zone Length (pixels)*	*Number of PCDs*	*Label*	*Meristematic zone Length (pixels)*	*Number of PCDs*
C3	650.308	0	Q1	636.572	3
C4	601.415	0	Q3	595.238	4
C5	584.411	0	Q6	586.276	1
C6	586.464	0	Q7	600.536	4
C7	609.908	0	Q8	635.437	3
C8	639.465	0	Q9	630.149	2
C10	558.516	0	Q10	586.464	4
C11	631.341	0	Q11	600.087	5
			Q12	619.681	3
			Q13	648.278	3
**Mean**	**607.729**	**0**	**Mean**	**613.872**	**3.2**
SD	31.26	**0**	SD	22.829	**1.135**

Figure S6: Quantification of cell division plane orientation defects in ground tissue layers in *Arabidopsis clv1/bam1/bam2/bam3* roots (related to Figure 3). (A) Diagram showing the nature of cell division plane orientation defects in wild-type versus *clv1/bam1/bam2/bam3* plants. (B) Equivalence of developmental stages used in comparisons between wild-type and mutant plants. (C) Quantitative data showing differences in the number of periclinal cell divisions (PCDs) in wild-type versus mutant plants.

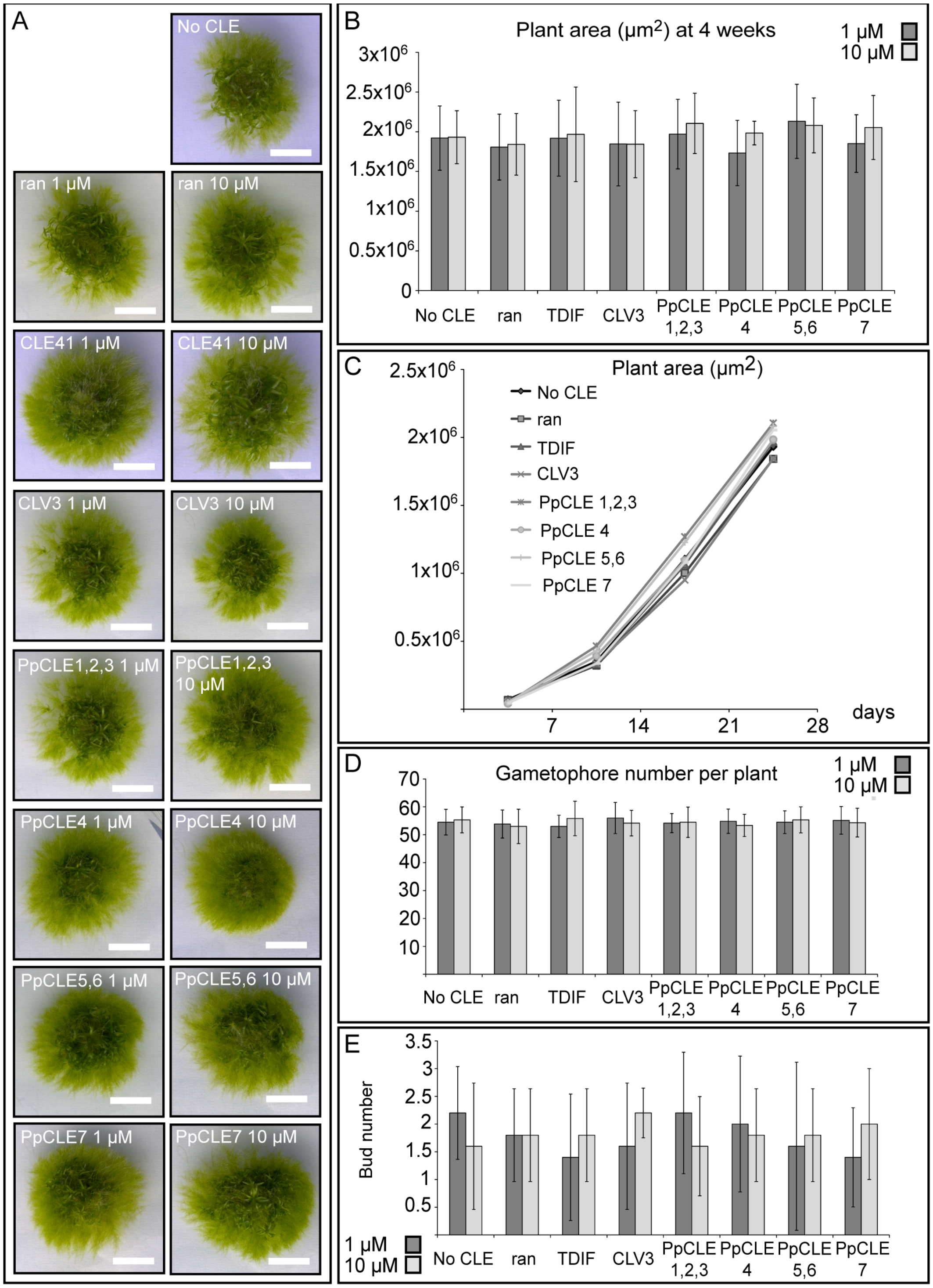

Figure S7: Treatment with CLE peptides had no appreciable effect on protonemal morphology (related to Figure 6). (A) Light micrographs of plants treated with a synthetic random peptide, CLE41, CLV3 or *Physcomitrella* CLEs showing morphology. Scale Bar = 1 cm. (B and C) The area of plants treated with 1 μM or 10 μM CLEs was no different from controls (n ≥ 20). (D and E) CLE treatment did not effect gametophore initiation. For D, gametophores from 5 plants were counted for each treatment, and for E, a 5 mm square from the edge of 5 plants was counted for each treatment.

Gene name	Gene ID	Source
CLE		
PpCLE1	Pp1s87_125V6.1	*Physcomitrella patens*
PpCLE2	Pp1s86_83V6.1	*Physcomitrella patens*
PpCLE3	Pp1s74_50V6.1	*Physcomitrella patens*
PpCLE4	Pp1s6_223V6.1	*Physcomitrella patens*
PpCLE5	Pp1s275_39V6.1	*Physcomitrella patens*
PpCLE6	Pp1s292_57V6.1	*Physcomitrella patens*
PpCLE7	Pp1s27_259V6.1	*Physcomitrella patens*
AaCLE1	MG571535	*Anthoceros agrestis*
DwCLE1	scaffold-UFJN-2003654-Diplazium_wichurae	*Diplazium wichurae*
DwCLE2	scaffold-UFJN-2087917-Diplazium_wichurae	*Diplazium wichurae*
AmtCLE1	>lcllevm_27.model.AmTr_v1.0_scaffold00022.262	*Amborella trichopoda*
AmtCLE2	>lcllevm_27.model.AmTr_v1.0_scaffold00030.123	*Amborella trichopoda*
AmtCLE3	>lcllevm_27.model.AmTr_v1.0_scaffold00021.96	*Amborella trichopoda*
AmtCLE4	>lcllevm_27.model.AmTr_v1.0_scaffold00007.246	*Amborella trichopoda*
AmtCLE5	>lcllevm_27.model.AmTr_v1.0_scaffold00002.301	*Amborella trichopoda*
AmtCLE6	>lcllevm_27.model.AmTr_v1.0_scaffold00021.106	*Amborella trichopoda*
AmtCLE7	>lcllevm_27.model.AmTr_v1.0_scaffold00010.83	*Amborella trichopoda*
AmtCLE8	>lcllevm_27.model.AmTr_v1.0_scaffold00067.109	*Amborella trichopoda*
CLV		
DwCLV1A	scaffold-UFJN-2012643	*Diplazium wichurae*
DwCLV1B	scaffold-UFJN-2014575	*Diplazium wichurae*
GmCLV1C	NP_001235065.1	*Glycine max*
GmCLV1D	NP_001237688.1	*Glycine max*
GmCLV1E	NP_001235080.1	*Glycine max*
GmCLV1F	NP_001237715.1	*Glycine max*
GmCLV1G	XP_003530709.1	*Glycine max*
GmCLV1H	XP_006602289.1	*Glycine max*
GmCLV1I	XP_003518489.2	*Glycine max*
GmCLV1J	XP_003545159.1	*Glycine max*
FON1	Os06g50340.1	*Oryza sativa*
OsCLV1A	Os03g0228800	*Oryza sativa*
OsCLV1B	Os07g0134200	*Oryza sativa*
OsCLV1C	Os05g0595950	*Oryza sativa*
PpCLV1A	Pp1s5_68V6.1	*Physcomitrella patens*
PpCLV1B	Pp1s14_447V6.1	*Physcomitrella patens*
PaCLV1A	MA_64117p0010	*Picea abies*
PaCLV1B	MA_943683p0010	*Picea abies*
PaCLV1C	MA_52165p0010	*Picea abies*
PaCLV1D	MA_120550p0010	*Picea abies*
SmCLV1	XP_002965214.1	*Selaginella moellendorffii*
SmCLV2	XP_002971751.1	*Selaginella moellendorffii*
SmCLV3	XP_002970036.1	*Selaginella moellendorffii*
AaCLV1A	MG571536	*Anthoceros agrestis*
AmtCLV1A	evm_27.model.AmTr_v1.0_scaffold00055.1	*Amborella trichopoda*
AmtCLV1B	evm_27.model.AmTr_v1.0_scaffold00033.36	*Amborella trichopoda*
AmtCLV1C	evm_27.model.AmTr_v1.0_scaffold00071.179	*Amborella trichopoda*
AmtCLV1D	evm_27.model.AmTr_v1.0_scaffold00068.165	*Amborella trichopoda*
AmtCLV1E	evm_27.model.AmTr_v1.0_scaffold00056.126	*Amborella trichopoda*
RPK2		
DwRPK2A	scaffold-UFJN_2014694	*Diplazium wichurae*
DwRPK2B	scaffold-UFJN_2002858	*Diplazium wichurae*
GmRPK2A	XP_003548492.2	*Glycine max*
GmRPK2B	XP_003530440.2	*Glycine max*
GmRPK2C	XP_003551760.1	*Glycine max*
GmRPK2D	XP_003543956.1	*Glycine max*
GmRPK2E	XP_003554916.1	*Glycine max*
OsRPK2A	Os07g0602700	*Oryza sativa*
OsRPK2B	Os03g0756200	*Oryza sativa*
PaRPK2A	MA_13025p0010	*Picea abies*
PaRPK2B	MA_10427820p0020	*Picea abies*
PaRPK2C	MA_129592p0010	*Picea abies*
SmRPK2	XP_002982473	*Selaginella moellendorffii*
AmtRPK2A	evm_27.model.AmTr_v1.0_scaffold00154.29	*Amborella trichopoda*
AmtRPK2B	evm_27.model.AmTr_v1.0_scaffold00016.228	*Amborella trichopoda*

Table S1: List of newly identified *CLE, CLV* and *RPK2* genes, gene IDs and species of origin (related to Figure 1).

Class	Species	Publication	Database searched
Seed plant	*Arabidopsis thaliana*	[S2] Strabala *et al.* (2006)	NCBI
Seed plant	*Oryza sativa*	[S3] Yu *et al.* (2002)	NCBI
Seed plant	*Glycine max*	[S4] Mortier *et al.* (2011)	NCBI
Seed plant	*Amborella trichopoda*	[S5] Amborella Genome Project (2013)	Phytozome
Seed plant	*Picea abies*	[S6] Strabala *et al.* (2014)	NCBI
Monilophyte	*Diplazium wichurae*	1kp project	1kp project
Lycophyte	*Selaginella moellendorffii*	[S7] Miwa *et al.* (2009)	NCBI
Hornwort	*Anthoceros agrestis*	Draft genome assembly (Szövényi)	Draft genome assembly (Szövényi)
Moss	*Physcomitrella patens*	[S7] Miwa *et al.* (2009)	COSMOSS
Liverwort	*Marchantia polymorpha*	[S8] Bowman *et al.* (2017)	Phytozome
Charophyte alga	*Coleochaete nitellarum*	1kp project	1kp project
Charophyte alga	*Spirogyra* sp.	[S9] Delaux *et al.* (2015)	Dunand lab
Charophyte alga	*Chara braunii*	Draft genome assembly (Rensing)	Draft genome assembly (Rensing)
Chlorophyte alga	*Ulva spp.*	1kp project	1kp project
Chlorophyte alga	*Chlamydomonas reinhardtii*	[S10] Merchant *et al.* (2007)	Phytozome
Chlorophyte alga	*Volvox carteri*	[S11] Prochnik *et al.* (2010)	Phytozome
Chlorophyte alga	*Ostreococcus tauri*	[S12] Palenik *et al.* (2007)	Phytozome
Chlorophyte alga	*Chlorella vulgaris*	[S13] Blanc *et al.* (2010)	Phytozome

Table S2: List of taxa, publications and databases searched for sequence data (related to Figure 1).

Gene name	V1.6 genome	V3 genome	Peptide encoded
PpCLE1	Pp1s87_125V6.1	Pp3c7_11040V1.1	PpCLE 1/2/3
PpCLE2	Pp1s86_83V6.1	Pp3c1_13720V1.1	PpCLE 1/2/3
PpCLE3	Pp1s74_50V6.1	Pp3c3_10020V1.1	PpCLE 1/2/3
PpCLE4	Pp1s6_223V6.1	Pp3c26_11430V1.1	PpCLE 4
PpCLE5	Pp1s275_39V6.1	Pp3c22_4590V1.1	PpCLE 5/6
PpCLE6	Pp1s292_57V6.1	Pp3c19_6950V1.1	PpCLE 5/6
PpCLE7	Pp1s27_259V6.1	Pp3c21_5600V1.1	PpCLE 7
PpCLE8	not found	Pp3c11_15310V1.1	PpCLE 1/2/3
PpCLE9	not found	Pp3c4_31330V1.1	PpCLE 1/2/3
PpCLV1a	Pp1s5_68V6.1	Pp3c13_13360V1.1	PpCLV1a
PpCLV1b	Pp1s14_447V6.1	Pp3c6_21940V1.1	PpCLV1b
PpRPK2	Pp1s311_57V6.1	Pp3c7_5570V1.1	PpRPK2

Table S3: List of *Physcomitrella* CLAVATA pathway V3 genome gene IDs (related to Figure 1).

Primer name	Primer sequence
A. Primers for RT-PCR	
cDNA synthesis primer [S14]	
Q_T	CCAGTGAGCAGAGTGACGAGGACTCGAGCT
PpCLE1	
PpCLE1F	GTAGCATTGAGGTTCACGACA
PpCLE1R	CACGGGAATATGACTTGAGA
PpCLE2	
PpCLE2F	CAGATGCGGTTGAGAAAGAGA
PpCLE2R	GACTTGAGACCGATTGCTGTT
PpCLE3	
PpCLE3F	GTAATCCTCGCCATTTTCCA
PpCLE3R	GGGTTTCGTGGATTCGTGAT
PpCLE4	
PpCLE4F	CGAAGGCAGACGACAGGTGA
PpCLE4R	GACCTGCGACCTGTTGCTATT
PpCLE5	
PpCLE5F	ACGTTGGTGCTGGATTGTGAT
PpCLE5R	TCTGCCTCCACATCCCAAAT
PpCLE6	
PpCLE6F	GTAGGAATGGTCGTCGTCGT
PpCLE6R	GAACCAAGCGCTTCGACAT
PpCLE7	
PpCLE7F	TGCTTGTCCATGGTGATTGT
PpCLE7R	CCCGACTGTGATCCAACTTT
PpCLV1a	
PpCLV1aF	CAACATCGCAATCCAGGCT
PpCLV1aR	CCACTCTCAGGACCAATACAA
PpCLV1b	
PpCLV1bF	GGCAATCTCCCCACCCT
PpCLV1bR	CTCCTCGTCCAAGCAGTCTA
PpRPK2	
PpRPK2F	GTGGACCCGTTTCGTGTGTT
PpRPK2R	GGCTGGTGGACCCTGATAA
PpUBI	
PpUbi-intF	GCCATGCAGATCTTCGTGAA
PpUbi-intR	CTACGCAGCCAAGAACCGA
B. Promoter::NGG construction	
PpCLE1	
CLE15'PF	gtttaaacGGCACCATCTCCATCACTATCT
CLE15'PR	gcgatcgccacgtgGGTAAGGCTCCATGCACCGT
PpCLE2	
CLE25'PF	CGCTGCTGATTCACCACCTCAA
CLE25'PR	GGCATAATGTGGGGAGAAGGA
PpCLE7	
CLE75'PF	CTTGTGACATTCTAATAAGTGCTTATCC
CLE75'PR	CCCTTCCGAAAAACTGATACCA
PpCLV1a	
CLV1aPF	tatggatccTCTGTCAAATTTATTACCACTT
CLV1aPR	tatggatccGAGGAAAGCATGAGCACTGA
PpCLV1b	
CLV1bPF	TTTTGGATCAGCCATCCCTATAAGGCTCAG
CLV1bPR	GGTTATTCATGTTTTCTAGACACTGTTGCT
PpRPK2	
RPK25'PF	cttaagATTATTTTTTGTTACCTTGTATTTT
RPK25'PR	gtttaaacTCTCCCCTAACTCCTCCTCA

C. Promoter::NGG screening primers

PlGF2	AGGACACCCTTTCCAAACACATT
PlGR1	AAAAACCAATCTGGGAATAGCTTG
G6TERM4F	TAGGGTTCTATAGGGTTTCGCTCA
CLE1SCREENR	ACAGATTGCAGTTCCGTATGCTC
CLE2SREENR	TAAGCATGCAGCTCTAGGAAACG
CLE7SCREENR	CCATTGGCTATTAAAATGGCTTGA
RPK2SCREENR	TCTCATTTGCAAGTATAATCCAAGC
CLV1ASCREENR	CGAGTGCAACGAGATTCAAA
CLV1BSCREENR	GCAATCGGACAGACCTTTGAGTA

D. PpcleAmiR *construction*

PpcleAmiR1-3	
123-I	gaTTGGGAACCATGCGGTCGGAGtctctcttttgtattcc
123-II	gaCTCCGACCGCATGGTTCCCAAtcaaagagaatcaatga
123-III	gaCTACGACCGCATGCTTCCCATtcacaggtcgtgatatg
123-IV	gaATGGGAAGCATGCGGTCGTAGtctacatatatattcct
amiR-A	CTGCAAGGCGATTAAGTTGGGTAAC
amiR-B	GCGGATAACAATTTCACACAGGAAACAG
PpcleAmiR4-7	
7-I	gaTTGAAGCGGATTAGGACCTGGtctctcttttgtattcc
7-II	gaCCAGGTCCTAATCCGCTTCAAtcaaagagaatcaatga
7-III	gaCCCGGTCCTAATCGGCTTCATtcacaggtcgtgatatg
7-IV	gaATGAAGCCGATTAGGACCGGGtctacatatatattcct
amiR-A	CTGCAAGGCGATTAAGTTGGGTAAC
amiR-B	GCGGATAACAATTTCACACAGGAAACAG

E. PpcleAmiR *screening*

Kanamycin resistance cassette	
Kan-F	GGCATGATTGAACAAGATGAT
Kan-R	TATCGGGAAACTACTCACACAT
Hygromycin resistance cassette	
Hyg-F	AGGGCGAAGAATCTCGTGCT
Hyg-R	GCTTAGCGAACTGTGGACGA
PpcleAmiR amplification	
AmiRscrF	CGGTCGGAGTCTCTCTTTTG
AmiRscrR	CGCTCGGTGTGTCGTAGATA
PpcleAmiR expression cassette	
UbiOCSF	GCCGAACCAGCTTTCTTGTA
UbiOCSR	GTTGAATGGTGCCCGTAACT
PpUBI CDS	
Pp-Ubi-intF	GCCATGCAGATCTTCGTGAA
Pp-Ubi-intR	CTACGCAGCCAAGAACCGA

F. Ppclv1a1b construction and sequencing

PpCLV1a sgRNA-1	GGCAGACAGTGCCCGAGGCTCTCT
PpCLV1a sgRNA-1*	AAACAGAGAGCCTCGGGCACTGTC
PpCLV1a sgRNA-2	GGCACCACGGGCATGTCCTGATAC
PpCLV1a sgRNA-2*	AAACGTATCAGGACATGCCCGTGG
PpCLV1b sgRNA	GGCAGAAGTGCGAGACCCTCTTC
PpCLV1b sgRNA*	AAACGAAGAGGGTCTCGCACTTC

G. Ppclv1a1b *screening*

PpCLV1a sgRNA targets fwd (exon 4)	AACGGCTCAATTCCTCCAGA
PpCLV1a sgRNA targets rev (exon 5)	TTAGACACTCCACCCTTGCG
PpCLV1b sgRNA target fwd	TGGAGAGACGCAACTTCCAT
PpCLV1b sgRNA target rev	TTAAGACGCCCCAAATCAGC

H. Pprpk2 *construction*	
5' flanking region	
PpRPK2-5'F	ATCGATGGCTCTGGAGGTGAGTGACA
PpRPK2-5'R	GTTTAAACAGTTCGAGACAACACAAGAATGC
3' flanking region	
PpRPK2-3'F	GTTTAAACGGCGCGCCGATGGTCGGCATAGTAAACG
PpRPK2-3'R	ATCGATCAGGACGACAAGGCGGA
I. Screening Pprpk2 *lines*	
Hygromycin resistance casette	
Hyg-F	AGGGCGAAGAATCTCGTGCT
Hyg-R	GCTTAGCGAACTGTGGACGA
3' integration site analysis	
Hygromycin-F	CGCACAATCCCACTATCCTT
PpRPK2downstream-R	CAAGAGTCAGCCAATGATGCA
PpRPK2 CDS	
PpRPK2F	GTGGACCCGTTTCGTGTGTT
PpRPK2R	GGCTGGTGGACCCTGATAA
PpUBI CDS	
PpUbi-intF	GCCATGCAGATCTTCGTGAA
PpUbi-intR	CTACGCAGCCAAGAACCGA
J. *Southern blot probes*	
PpRPK2 probe	
PpRPK2probe-F	GTGGACCCGTTTCGTGTGTT
PpRPK2probe-R	GGCTGGTGGACCCTGATAA
Hygromycin probe	
Hygprobe-F	CGCACAATCCCACTATCCTT
Hygprobe-R	GATGTTGGCGACCTCGTATT
K. Identification of Arabidopsis rpk-2 *homozygotes*	
dCAPS primers	
AtRPK2-BamHI-F	CACATCTTGAGAGATTTCTGCTTTGTAGGTGGATC
AtRPK2-BamHI-R	GAGAAGTCACTATGTTCATGGATAT

Table S4: List of primers used in this study (related to Figures 2-4 and STAR methods).

Supplemental references

S1. Ortiz-Ramírez, C., Hernandez-Coronado, M., Thamm, A., Catarino, B., Wang, M., Dolan, L., Feijó, J.A., and Becker, J.D. (2016). A transcriptome atlas of *Physcomitrella patens* provides insights into the evolution and development of land plants. Molecular Plant *9*, 205-220.

S2. Strabala, T.J., O'Donnell, P.J., Smit, A.-M., Ampomah-Dwamena, C., Martin, E.J., Netzler, N., Nieuwenhuizen, N.J., Quinn, B.D., Foote, H.C., and Hudson, K.R. (2006). Gain-of-function phenotypes of many CLAVATA3/ESR genes, including four new family members, correlate with tandem variations in the conserved CLAVATA3/ESR domain. Plant Physiology *140*, 1331-1344.

S3. Yu, J., Hu, S., Wang, J., Wong, G.K.-S., Li, S., Liu, B., Deng, Y., Dai, L., Zhou, Y., and Zhang, X. (2002). A draft sequence of the rice genome (*Oryza sativa* L. ssp. *indica*). Science *296*, 79-92.

S4. Mortier, V., Fenta, B.A., Martens, C., Rombauts, S., Holsters, M., Kunert, K., and Goormachtig, S. (2011). Search for nodulation-related CLE genes in the genome of *Glycine max*. Journal of Experimental Botany *62*, 2571-2583.

S5. Chamala, S., Chanderbali, A.S., Der, J.P., Lan, T., Walts, B., Albert, V.A., Leebens-Mack, J., Rounsley, S., Schuster, S.C., and Wing, R.A. (2013). Assembly and validation of the genome of the nonmodel basal angiosperm *Amborella*. Science *342*, 1516-1517.

S6. Strabala, T.J., Phillips, L., West, M., and Stanbra, L. (2014). Bioinformatic and phylogenetic analysis of the CLAVATA3/EMBRYO-SURROUNDING REGION (CLE) and the CLE-LIKE signal peptide genes in the Pinophyta. BMC plant biology *14*, 47.

S7. Miwa, H., Tamaki, T., Fukuda, H., and Sawa, S. (2009). Evolution of CLE signaling: origins of the CLV1 and SOL2/CRN receptor diversity. Plant signaling & behavior *4*, 477-481.

S8. Bowman, J.L., Kohchi, T., Yamato, K.T., Jenkins, J., Shu, S., Ishizaki, K., Yamaoka, S., Nishihama, R., Nakamura, Y., and Berger, F. et al. (2017). Insights into land plant evolution garnered from the *Marchantia polymorpha* genome. Cell *171*, 287-304. e215.

S9. Delaux, P-M., Radhakrishnan, G.V., Jayaraman, D., Cheema, J., Malbreil, M., Volkening, J.D., Sekimoto, H., Nishiyama, T., Melkonian, M., and Pokorny, L. (2015). Algal ancestor of land plants was preadapted for symbiosis. *PNAS 112*, 13390-13395.

S10. Merchant, S.S., Prochnik, S.E., Vallon, O., Harris, E.H., Karpowicz, S.J., Witman, G.B., Terry, A., Salamov, A., Fritz-Laylin, L.K., and Maréchal-Drouard, L. (2007). The *Chlamydomonas* genome reveals the evolution of key animal and plant functions. Science *318*, 245-250.

S11. Prochnik, S.E., Umen, J., Nedelcu, A.M., Hallmann, A., Miller, S.M., Nishii, I., Ferris, P., Kuo, A., Mitros, T., and Fritz-Laylin, L.K. (2010). Genomic analysis of organismal complexity in the multicellular green alga *Volvox carteri*. Science *329*, 223-226.

S12. Palenik, B., Grimwood, J., Aerts, A., Rouzé, P., Salamov, A., Putnam, N., Dupont, C., Jorgensen, R., Derelle, E., and Rombauts, S. (2007). The tiny eukaryote *Ostreococcus* provides genomic insights into the paradox of plankton speciation. PNAS *104*, 7705-7710.

S13. Blanc, G., Duncan, G., Agarkova, I., Borodovsky, M., Gurnon, J., Kuo, A., Lindquist, E., Lucas, S., Pangilinan, J., and Polle, J. (2010). The *Chlorella variabilis* NC64A genome reveals adaptation to photosymbiosis, coevolution with viruses, and cryptic sex. The Plant Cell *22*, 2943-2955.

S14. Frohman, M.H. (1995). Rapid amplification of cDNA ends. In PCR Primer, C.W. Diffenbach and G.S. Dveksler, eds. (Cold Spring Harbor, NY.: Cold Spring Harbor Laboratory Press), pp. 381–409.

S15. Ishikawa, M., Murata, T., Sato, Y., Nishiyama, T., Hiwatashi, Y., Imai, A., Kimura, M., Sugimoto, N., Akita, A., Oguri, Y., et al. (2011). *Physcomitrella* Cyclin-Dependent Kinase A links cell cycle reactivation to other cellular changes during reprogramming of leaf cells. The Plant Cell *23*, 2924-2938.

S16. Moody, L., Kelly, S., Coudert, Y., Nimchuk, Z., Harrison, C.J. and Langdale, J. (2018). Somatic hybridization provides segregating populations for the identification of causative mutations in sterile mutants of the moss *Physcomitrella patens*. New Phytologist *218*, 1270-1277.
