# Supplementary material for: CLAVATA Was a Genetic Novelty for the Morphological Innovation of 3D Growth in Land Plants
Source: Curr Biol. 2018 Aug 6;28(15):2365–2376.e5. doi: 10.1016/j.cub.2018.05.068 (PMC6089843; doi:10.1016/j.cub.2018.05.068)
Supplement: Methods S1. Additional Data Outlining Strategies for Generating promoter::NLSGUSGFP Reporter Lines, PpcleAmiR Mutants, Ppclv1 CRISPR/Cas9 Mutants, and Pprpk2 KO Mutants, Related to STAR Methods [file mmc5.pdf]

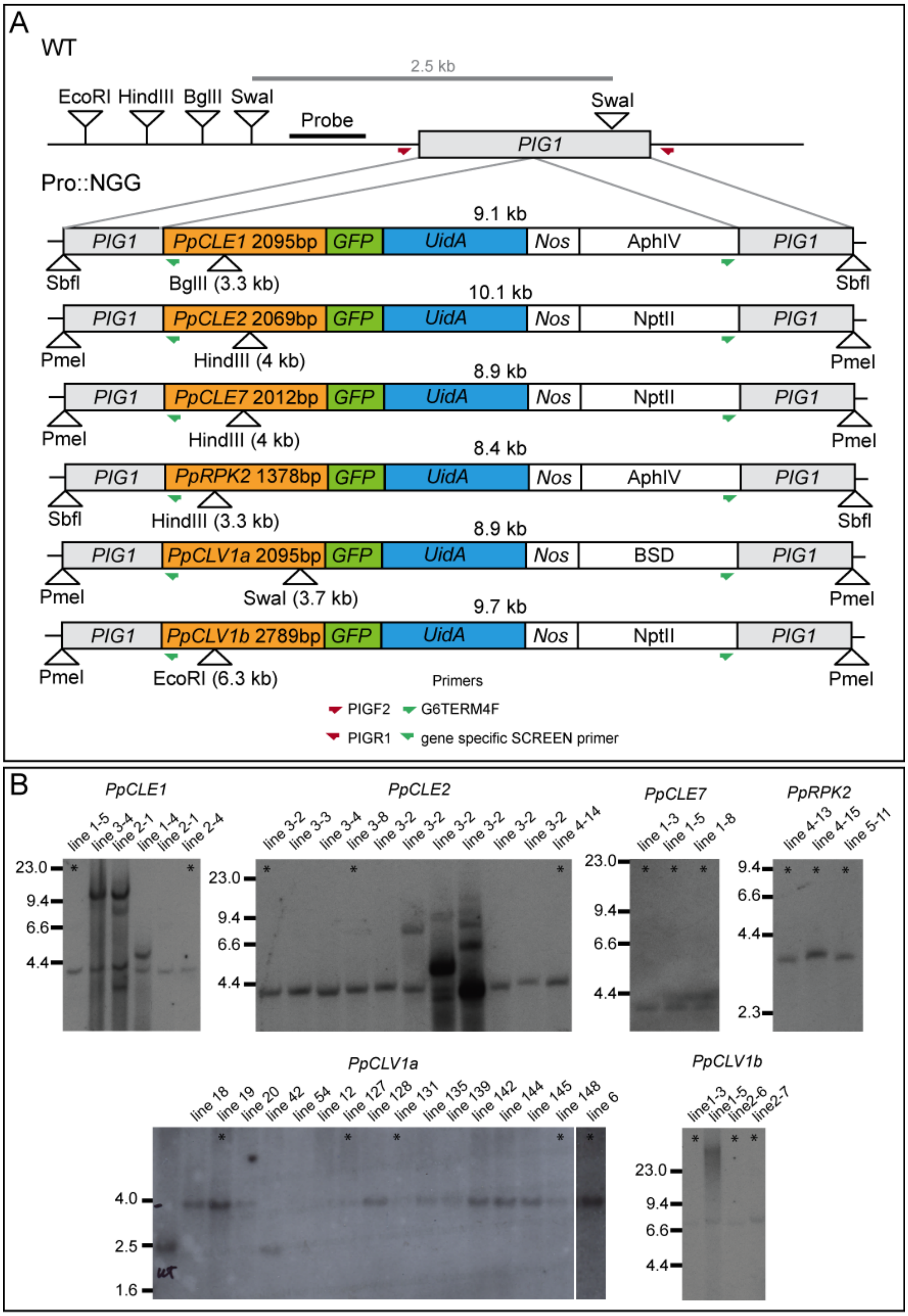

Strategy for generation of *promoter::NLSGUSGFP* reporter lines. (A) Promoter fragments of *PpCLE1*, *PpCLE2*, *PpCLE7*, *PpRPK2*, *PpCLV1a* and *PpCLV1b* of varying lengths were PCR amplified and cloned into the SmaI site of the PIG1NGGII [S15] vector with or without replacement of the BSD casette with an NptII casette or an AphIV cassette, and transgenes were delivered into plants as PmeI or SbfI fragments. Lines were screened first by PCR using PIGF2 and gene specific SCREEN primers, and PIGR2 and G6TERMF primers. (B) Subsequent screening was undertaken by Southern analysis with a 5' PIG locus-specific probe following BglII, HindIII, SwaI or EcoRI digestion. Targeted single insertant lines were used in expression analyses.

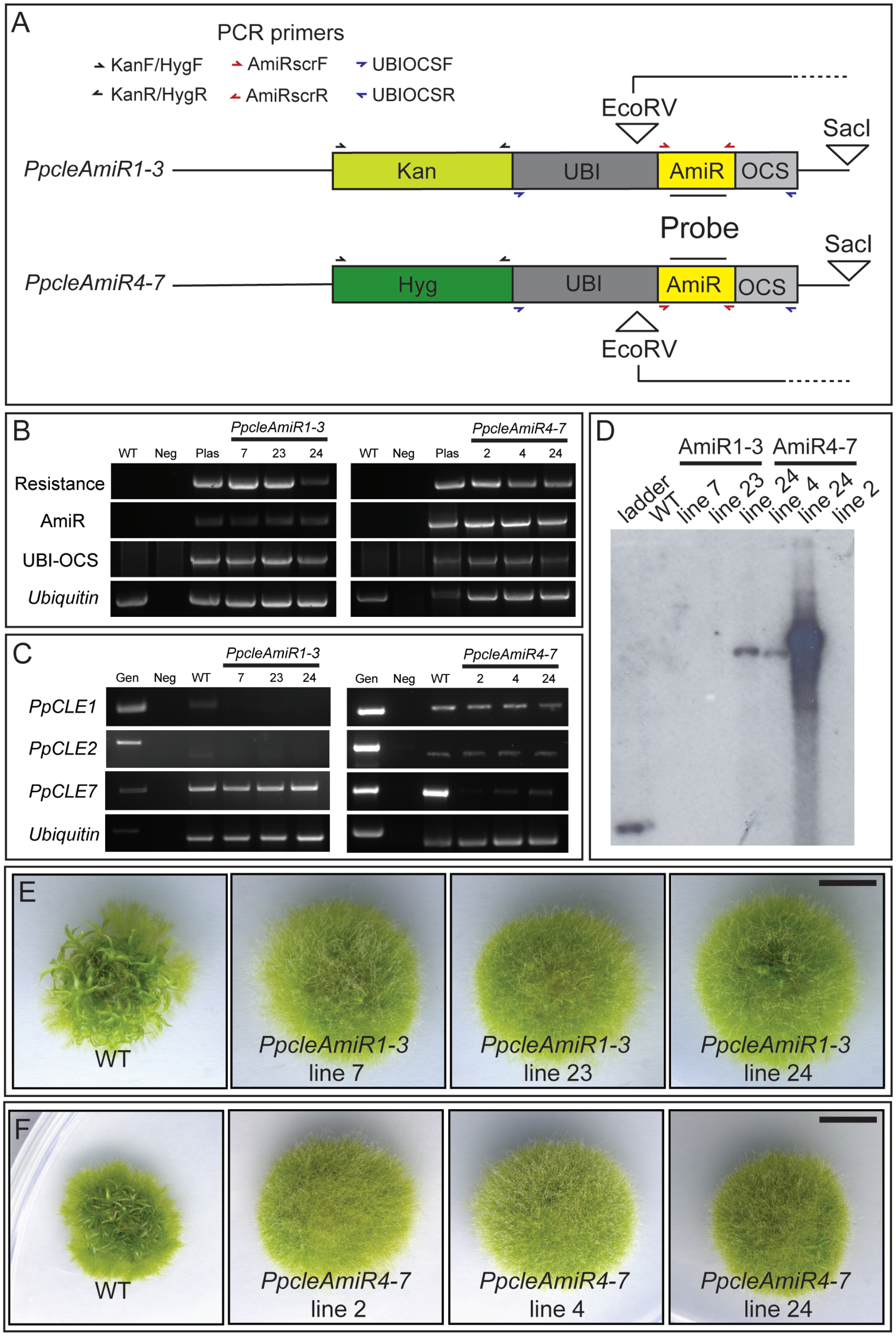

Strategy for generating *PpcleAmiR* lines. (A) Plasmids were constructed as described in STAR Methods and linearized with SacI prior to transformation. (B) PCR analysis identified three positive lines for each AmiR construct. (C) RT-PCR showed that *PpCLE* expression was strongly reduced in transgenic lines. (D) Southern analysis showed that one *PpcleAmiR1-3* and two *PpcleAmiR4-7* lines were stable insertants. (E and F) Multiple *PpcleAmiR1-3* (E) and *PpcleAmiR4-7* (F) lines had similar mutant phenotypes. Scale bars = 1 cm.

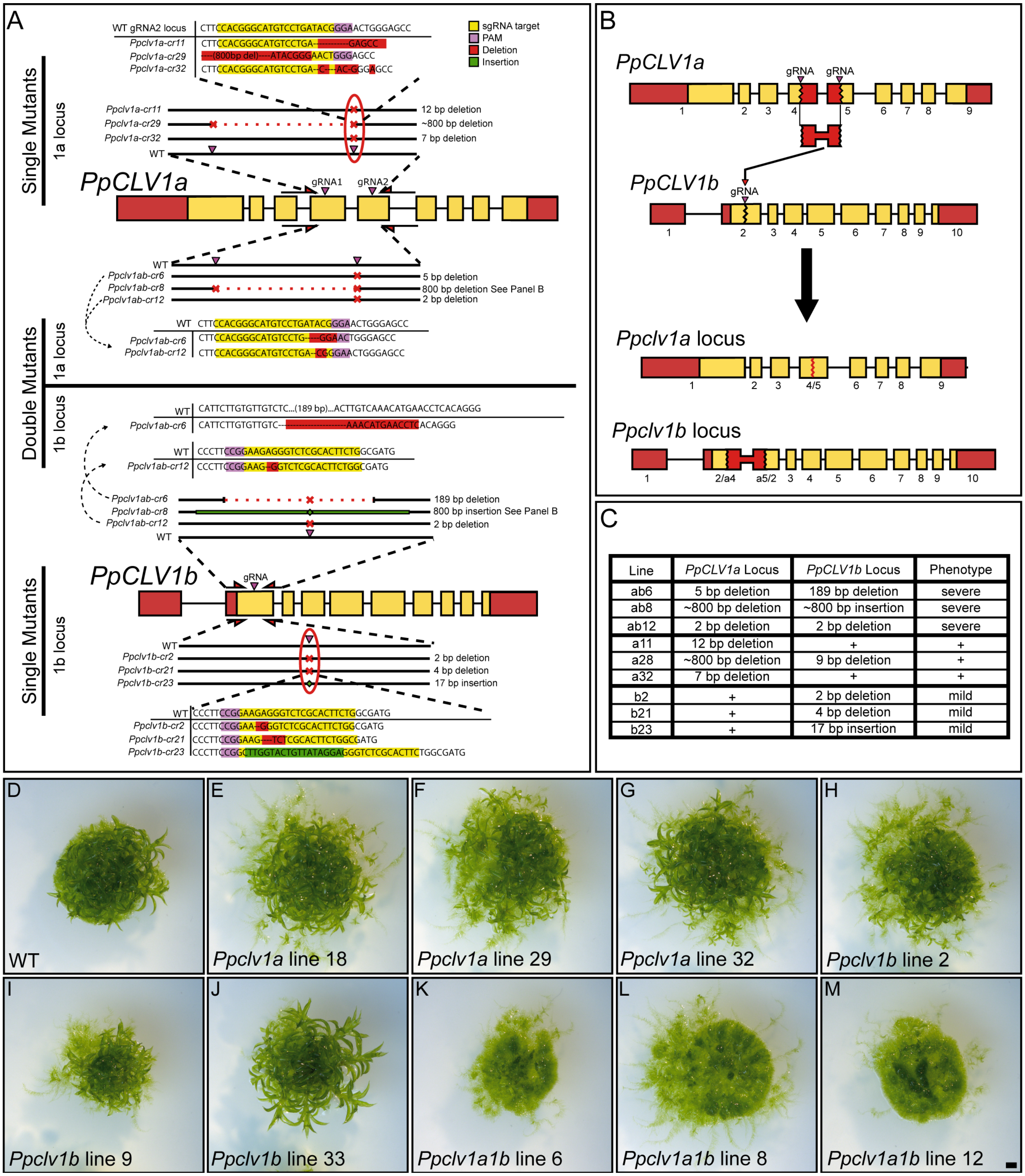

Line	*PpCLV1a* Locus	*PpCLV1b* Locus	Phenotype
ab6	5 bp deletion	189 bp deletion	severe
ab8	~800 bp deletion	~800 bp insertion	severe
ab12	2 bp deletion	2 bp deletion	severe
a11	12 bp deletion	+	+
a28	~800 bp deletion	9 bp deletion	+
a32	7 bp deletion	+	+
b2	+	2 bp deletion	mild
b21	+	4 bp deletion	mild
b23	+	17 bp insertion	mild

CRISPR/Cas9 strategy for generating *Ppclv1* mutants. (A) Generation of *Ppclv1a* single mutants, *Ppclv1ab* double mutants, and *Ppclv1b* mutants. Guide RNAs were designed to target *PpCLV1a* or *PpCLV1b* as indicated by purple triangles, and plants were co-transformed as described in STAR Methods. Locus-specific PCR amplification used primers illustrated by red arrows with sequences listed in STAR Methods, and Sanger sequencing was used to identify the nature of mutation in three independent disruptant lines. Sequences are colour coded to illustrate gRNA targets (yellow), protospacer adjacent motifs (PAM: purple), a deletion (green) and insertions (red). In gene models, UTRs are indicated in red and coding sequence is indicated in yellow.

Exons are represented as boxes, and introns are represented as lines. (B) In *Ppclv1a1b-8* mutants, the region between two gRNAs targeting *PpCLV1a* translocated into the site cut by a gRNA targeting *PpCLV1b*. (C) Summary of mutations at each locus in nine *PpCLV1* mutant lines described in (A). + denotes wild type sequence or phenotype. (D-M) Multiple *Ppclv1a*, *Ppclv1b* and *Ppclv1a1b* mutant lines had similar mutant phenotypes. Whereas WT and single mutant plants had well-developed gametophores, double mutant plants comprising mainly protonemata, and few gametophores were visible. Scale bar = 1 mm.

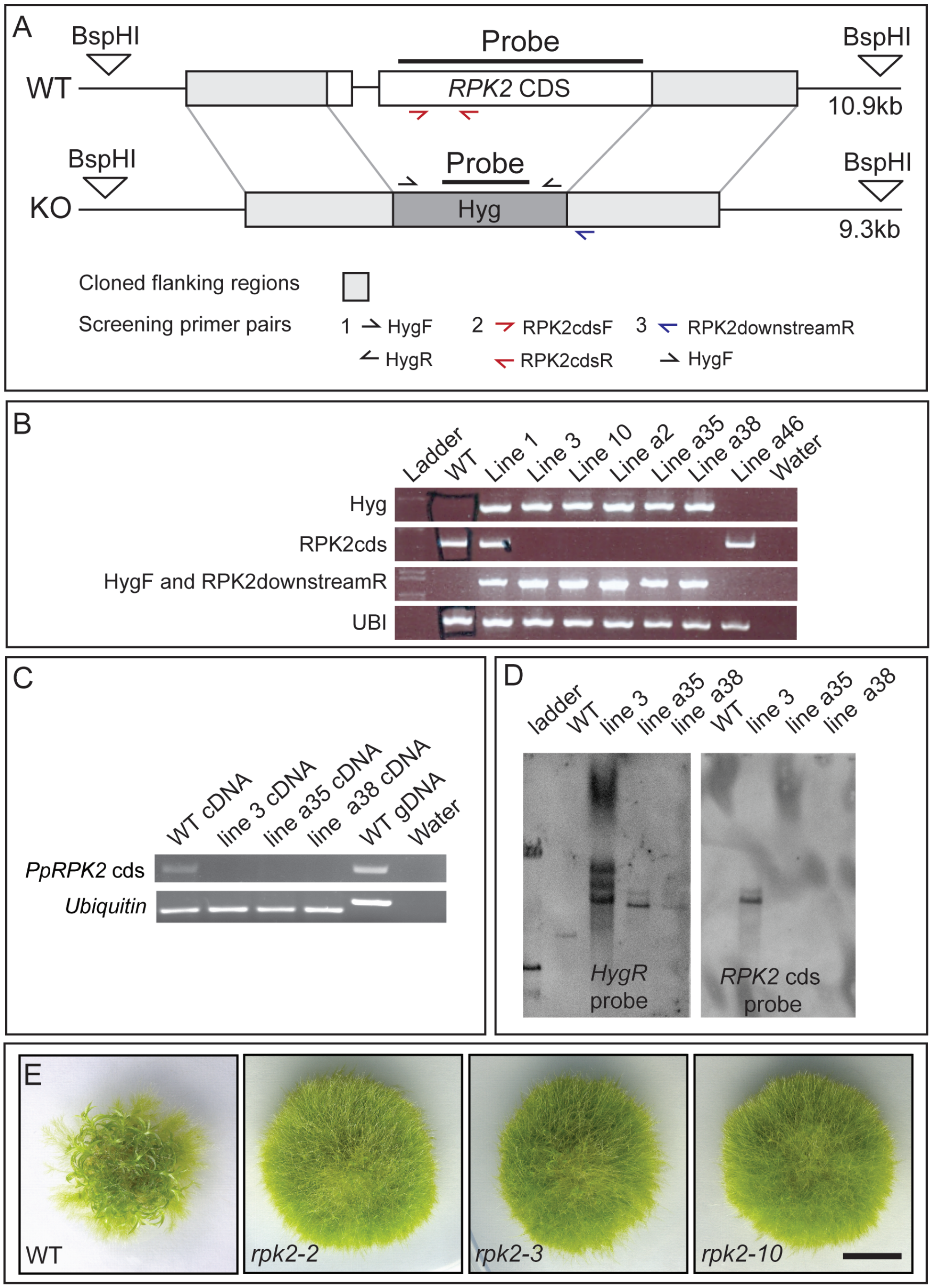

Strategy for generating *Pprpk2* KO lines. (A) To generate *Pprpk2* targeted deletion vectors, 5' and 3' *PpRPK2* flanking regions were PCR amplified and ligated into pGEM-TEASY™ . The 5' flanking sequence was excised with SacI and PmeI and inserted in tandem with the 3' flanking sequence prior to insertion of a Hygromycin resistance casette from pBHRF108 [S16] to generate the targeting vector. This was linearised with AflII for plant transformation. Stable insertants were screened by PCR with three primer pairs 1, 2 and 3. The *RPK2* locus is in a repeat rich genomic region, and we were unable to amplify fragments to screen for integration at the 5' end as Southern hybridization with several probes outside the insertion cassette gave smears. (B) PCR screening identified three candidate targeted deletion lines. (C) RT-PCR confirmed loss of *PpRPK2* expression in mutants. (D) Targeted deletion was confirmed by Southern analysis using sequential hybridization with *PpRPK2* and HygR probes at 42 ˚C. (E) Multiple knockout lines had similar mutant phenotypes. Scale bar = 1 cm.
