# Supplementary material for: CLAVATA Was a Genetic Novelty for the Morphological Innovation of 3D Growth in Land Plants
Source: Curr Biol. 2018 Aug 6;28(15):2365–2376.e5. doi: 10.1016/j.cub.2018.05.068 (PMC6089843; doi:10.1016/j.cub.2018.05.068)
Supplement: Document S2. Article plus Supplemental Information [file mmc6.pdf]

Article

Current Biology

CLAVATA Was a Genetic Novelty for the Morphological Innovation of 3D Growth in Land Plants

Highlights

- CLAVATA originated in the last common ancestor of land plants
- CLAVATA regulates cell proliferation, fate, and growth in *Physcomitrella*
- CLAVATA orients cell division planes in *Physcomitrella* and *Arabidopsis*
- CLEs act via receptors that are conserved between *Physcomitrella* and *Arabidopsis*

Authors

Chris D. Whitewoods,
Joseph Cammarata,
Zoe Nemec Venza, ...,
Adrienne H.K. Roeder,
Michael J. Scanlon, C. Jill Harrison

Correspondence

jill.harrison@bristol.ac.uk

In Brief

Whitewoods, Cammarata, et al. show that a conserved CLAVATA (CLV) pathway arose in the last common ancestor of land plants. CLV regulates cell division plane orientation during the 2D to 3D growth transition in a moss, and roles for CLV are shared between mosses and flowering plants, suggesting that CLV enabled 3D growth to arise in land plants.

Whitewoods et al., 2018, Current Biology *28*, 2365–2376
August 6, 2018 © 2018 The Authors. Published by Elsevier Ltd.
https://doi.org/10.1016/j.cub.2018.05.068

Current Biology
Article

CellPress

CLAVATA Was a Genetic Novelty for the Morphological Innovation of 3D Growth in Land Plants

Chris D. Whitewoods,[1,9,12] Joseph Cammarata,[1,2,3,12] Zoe Nemec Venza,[4] Stephanie Sang,[4,10] Ashley D. Crook,[5] Tsuyoshi Aoyama,[1,4,11] Xiao Y. Wang,[1] Manuel Waller,[6] Yasuko Kamisugi,[7] Andrew C. Cuming,[7] Péter Szövényi,[6] Zachary L. Nimchuk,[5,8] Adrienne H.K. Roeder,[2,3] Michael J. Scanlon,[2] and C. Jill Harrison[1,4,13,*]
[1]Plant Sciences Department, Cambridge University, Downing Street, Cambridge CB2 3EA, UK
[2]Plant Biology Section, School of Integrative Plant Science, Cornell University, Tower Road, Ithaca, NY 14853, USA
[3]Weill Institute for Cell and Molecular Biology, Cornell University, Ithaca, NY 14853, USA
[4]School of Biological Sciences, University of Bristol, 24 Tyndall Avenue, Bristol BS8 1TQ, UK
[5]Department of Biology, University of North Carolina at Chapel Hill, Chapel Hill, NC 27599, USA
[6]Department of Systematic and Evolutionary Botany, University of Zurich, Zollikerstrasse 107, 8008 Zurich, Switzerland
[7]Centre for Plant Sciences, Faculty of Biological Sciences, University of Leeds, Leeds LS2 9JT, UK
[8]Curriculum in Genetics and Molecular Biology, University of North Carolina at Chapel Hill, Chapel Hill, NC 27599, USA
[9]Present address: John Innes Centre, Norwich Research Park, Colney Lane, Norwich NR4 7UH, UK
[10]Present address: Department of Organismal Biology and Anatomy, The University of Chicago, 1027 E. 57th Street, Chicago, IL 60637, USA
[11]Present address: National Institute for Basic Biology, Nishigonaka 38, Myodaiji, Okazaki, 444-8585 Aichi, Japan
[12]These authors contributed equally
[13]Lead Contact
*Correspondence: jill.harrison@bristol.ac.uk
https://doi.org/10.1016/j.cub.2018.05.068

SUMMARY

How genes shape diverse plant and animal body forms is a key question in biology. Unlike animal cells, plant cells are confined by rigid cell walls, and cell division plane orientation and growth rather than cell movement determine overall body form. The emergence of plants on land coincided with a new capacity to rotate stem cell divisions through multiple planes, and this enabled three-dimensional (3D) forms to arise from ancestral forms constrained to 2D growth. The genes involved in this evolutionary innovation are largely unknown. The evolution of 3D growth is recapitulated during the development of modern mosses when leafy shoots arise from a filamentous (2D) precursor tissue. Here, we show that a conserved, CLAVATA peptide and receptor-like kinase pathway originated with land plants and orients stem cell division planes during the transition from 2D to 3D growth in a moss, *Physcomitrella*. We find that this newly identified role for CLAVATA in regulating cell division plane orientation is shared between *Physcomitrella* and *Arabidopsis*. We report that roles for CLAVATA in regulating cell proliferation and cell fate are also shared and that CLAVATA-like peptides act via conserved receptor components in *Physcomitrella*. Our results suggest that *CLAVATA* was a genetic novelty enabling the morphological innovation of 3D growth in land plants.

INTRODUCTION

The conquest of land was enabled by a series of innovations that allowed plant forms to radiate and occupy new volumes of space in the sub-aerial environment [1]. Among these, the innovation of shooting systems with organs positioned radially around an upright stem stands out as a primer for massively increased plant productivity and diversity [1]. Such three-dimensional (3D) growth forms first arose as a consequence of a novel stem cell function gained by land plants, namely the capacity to rotate stem cell divisions through multiple plane orientations [1–3]. The algal sister lineages of land plants are unable to rotate stem cell divisions through multiple planes and are therefore generally constrained to smaller filamentous or mat-like (two-dimensional [2D]) growth forms (Figure 1A) [1, 3]. The evolutionary transition from 2D to 3D growth is recapitulated during the development of modern mosses when a branching, filamentous (protonemal) precursor tissue (2D) gives rise to 3D gamete-producing leafy shoots (gametophores) [6]. Previous studies have shown that gametophores and filament branches initiate similarly as hemispherical outgrowths from parent filaments and that their divergent 2D or 3D fates are specified stochastically by APETALA2-type (APB) transcription factor activity [7]. During a single-celled stage of outgrowth development, persistent APB activity and cell swelling mark a switch to gametophore fate (3D), whereas loss of APB activity marks filament fate (2D) [6, 7]. A strongly oblique cell division is the first reliable morphological marker of gametophore development [6, 7]. This is followed by a second oblique apical cell division, which is approximately perpendicular to the first, after which division planes rotate during two successive rounds of division to establish a tetrahedral apical stem cell [6]. The tetrahedral apical cell divides in spiraling

 © 2018 The Authors. Published by Elsevier Ltd.
This is an open access article under the CC BY license (http://creativecommons.org/licenses/by/4.0/).

A

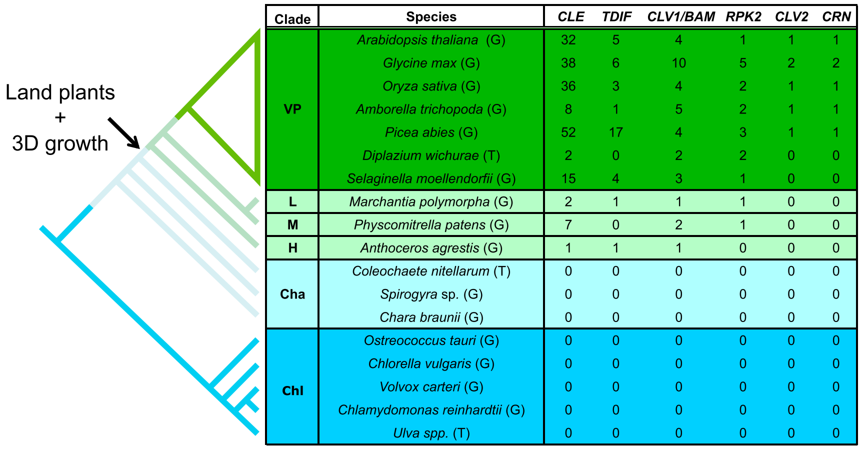

B

Clade	Species	*CLE*	*TDIF*	*CLV1/BAM*	*RPK2*	*CLV2*	*CRN*
VP	*Arabidopsis thaliana* (G)	32	5	4	1	1	1
	Glycine max (G)	38	6	10	5	2	2
	Oryza sativa (G)	36	3	4	2	1	1
	Amborella trichopoda (G)	8	1	5	2	1	1
	Picea abies (G)	52	17	4	3	1	1
	Diplazium wichurae (T)	2	0	2	2	0	0
	Selaginella moellendorfii (G)	15	4	3	1	0	0
L	*Marchantia polymorpha* (G)	2	1	1	1	0	0
M	*Physcomitrella patens* (G)	7	0	2	1	0	0
H	*Anthoceros agrestis* (G)	1	1	1	0	0	0
Cha	*Coleochaete nitellarum* (T)	0	0	0	0	0	0
	Spirogyra sp. (G)	0	0	0	0	0	0
	Chara braunii (G)	0	0	0	0	0	0
Chl	*Ostreococcus tauri* (G)	0	0	0	0	0	0
	Chlorella vulgaris (G)	0	0	0	0	0	0
	Volvox carteri (G)	0	0	0	0	0	0
	Chlamydomonas reinhardtii (G)	0	0	0	0	0	0
	Ulva spp. (T)	0	0	0	0	0	0

Figure 1. The CLV Pathway Originated in the Last Common Ancestor of Land Plants, Concomitantly with 3D Growth

(A) Phylogenetic relationships among land plants and their freshwater algal sister lineages redrawn from [4] and [5], respectively. Although chlorophytes and charophytes undergo stem cell divisions in a single orientation (2D growth), land plants undergo stem cell divisions in multiple orientations to generate elaborate three-dimensional forms (3D growth).

(B) The number of CLV pathway homologs was determined by BLAST against genome or draft genome (G) and transcriptome (T) databases as described in STAR Methods. Cha, charophytes; Chl, chlorophytes; H, hornworts; L, liverworts; M, mosses; VP, vascular plants.

See also Figures S1, S2, and S3 and Table S1.

planes to replace itself and produce daughter cells that generate the 3D gametophore axis and leaves [6]. The mechanisms regulating such novel and rotating stem cell division plane orientations during evolutionary and developmental transitions to 3D growth are unknown.

In *Arabidopsis*, the *CLAVATA* (*CLV*) and *WUSCHEL* (*WUS*) pathways act in a feedback loop to regulate many aspects of stem cell function, including cell fate [8, 9], proliferation [9–11], and growth [12]. *CLV3* encodes a small, secreted peptide that is expressed in the upper cell layers of the central zone and can move throughout the meristem [13–15]. *CLV1* is expressed in the underlying cell layers of the central zone and encodes a receptor-like kinase that acts as a receptor for CLV3 [11, 16] in conjunction with CLV2, CORYNE (CRN), RECEPTOR-LIKE PROTEIN KINASE 2 (RPK2), and BARELY ANY MERISTEM (BAM) [17, 18]. *WUS* activity promotes meristem cell proliferation [19], and CLV signaling restricts the size of the *WUS* expression domain [13]. WUS acts non-cell autonomously, moving from the organizing center to the uppermost meristem cell layers, where it promotes *CLV3* expression [20], thereby closing the feedback loop that maintains meristem size.

RESULTS

The CLAVATA Pathway Originated in the Last Common Ancestor of Land Plants

To determine how the CLV pathway evolved and identify potential roles for CLV in *Physcomitrella* stem cell function, we first queried publicly accessible genome and transcriptome databases from a wide range of green algae and land plants for *CLV3*-like (*CLE*), *CLV1/BAM*, *RPK2*, *CLV2*, and *CRN* homologs (Figure 1B; Table S1). We found no CLV pathway homologs in the chlorophyte or charophyte algae sampled but found at least one *CLE* homolog and one *CLV1/BAM* homolog in each early-diverging bryophyte lineage and all other land plants, suggesting that the core CLV signaling module comprises at least one CLE peptide and a CLV/BAM receptor-like kinase. *RPK2* homologs were present in all land plants sampled except the hornwort, *Anthoceros agrestis*. In *Physcomitrella*, we identified seven genes with a conserved *CLE* domain encoding a 12-amino-acid peptide motif similar to CLV3, but sequences outside the conserved CLE domain were divergent (Figure 1; Table S1). The genome encodes four CLV3-like peptides: *PpCLE*s *1*, *2*, and *3* encode the peptide motif RMVPTGPNPLHN; *PpCLE4* encodes the motif RMVPSGPNPLHN; *PpCLE*s *5* and *6* encode the motif RLVPTGPNPLHN; and *PpCLE7* encodes the motif RVVPTGPNPLHN. Neighbor-joining phylogenetic reconstructions showed that, although hornworts and liverworts have *CLE*s resembling the tracheary element differentiation inhibitory factor (TDIF)-like *CLE*s that regulate vascular development in *Arabidopsis*, *Physcomitrella* does not, consistent with an evolutionary loss in mosses (Figure S1; Data S1). Receptor-like kinase phylogenies were reconstructed by maximum likelihood analysis using amino acids from the conserved kinase domain (Figures S2 and S3; Data S2 and S3). Clades encompassing *CLV1/BAM*-like sequences from each land plant lineage or containing *RPK2*-like sequences from each lineage except hornworts were resolved. Both *CLV1/BAM* and *RPK2* phylogenies were broadly congruent with current hypotheses of land plant evolution [4, 21], thereby indicating orthology. Two *Physcomitrella* genes were incorporated in the *CLV1/BAM* clade, and these were named *Physcomitrella CLAVATA1a* and *1b* (*PpCLV1a* and *PpCLV1b*). One *RPK2* homolog was found and named *PpRPK2*, but no *CLV2* or *CRN* homologs were found. These sequence data indicate that the core components of the CLV pathway first arose in the last common ancestor of land plants, alongside the evolutionary innovation of 3D growth [22].

Physcomitrella CLAVATA Pathway Components Are Expressed during the 3D Growth Phase

To investigate *Physcomitrella* CLV activity, we first analyzed gene expression patterns in relation to the transition between 2D filamentous and 3D gametophore growth (Figures 2, S4, and S5). By RT-PCR, we detected *PpCLE1*, *2*, and *7* peptide-encoding gene expression in gametophores (Figure S4). We were unable to detect expression of *PpCLE*s *3*, *4*, and *5*, but we found *PpCLE6* expression in protonemal filaments. Receptor-encoding genes *PpRPK2*, *PpCLV1a*, and *PpCLV1b* were co-expressed in gametophores, although *PpRPK2* expression was evident earlier than *PpCLV1a* and *PpCLV1b*

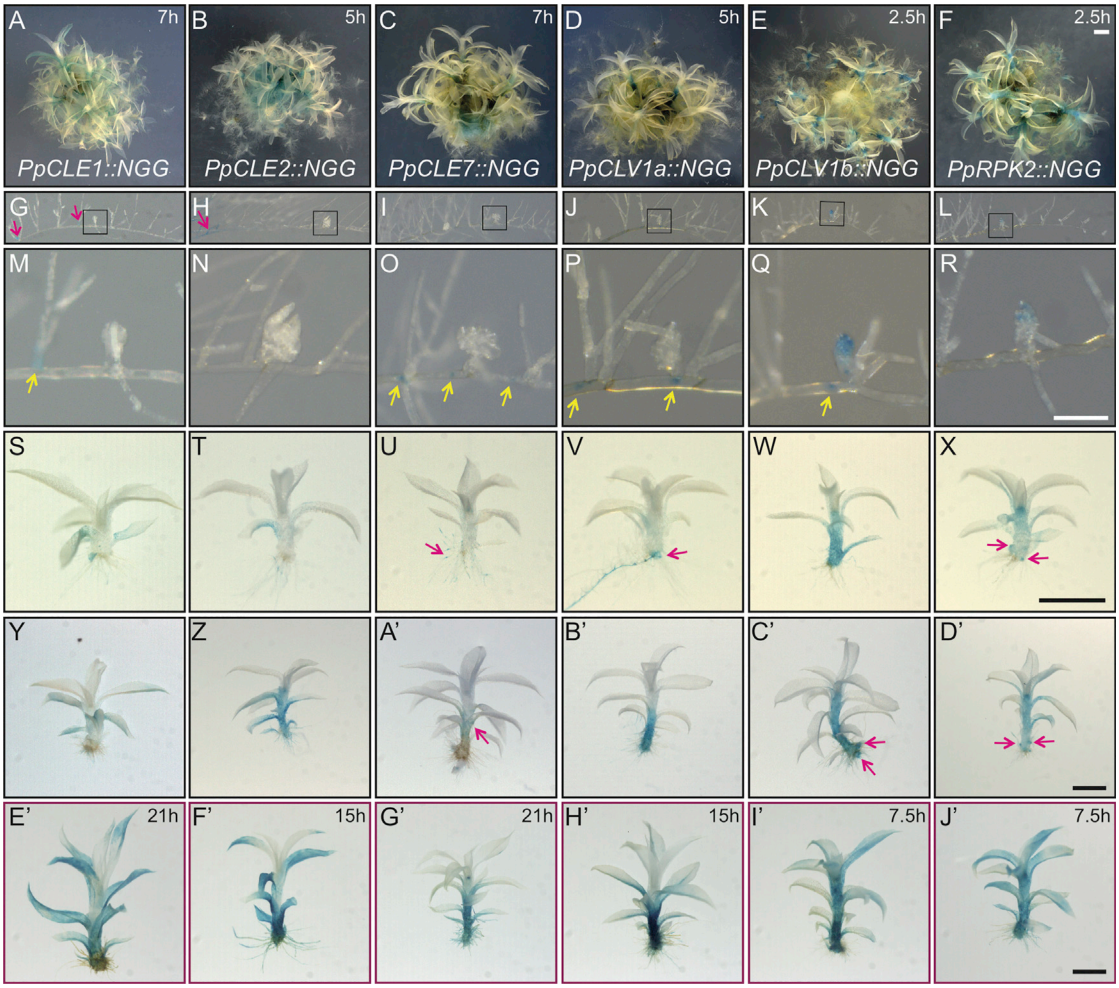

Figure 2. CLV Pathway Components Are Expressed in *Physcomitrella* Protonemata and Gametophores

(A– J') GUS staining of *PpCLE1::NGG* (A, G, M, S, Y, and E'), *PpCLE2::NGG* (B, H, N, T, Z, and F'), *PpCLE7::NGG* (C, I, O, U, A', and G'), *PpCLV1a::NGG* (D, J, P, V, B', and H'), *PpCLV1b::NGG* (E, K, Q, W, C', and I'), and *PpRPK2::NGG* (F, L, R, X, D', and J') lines revealed complex expression dynamics.

Although *PpCLE::NGG* and *PpCLV1a::NGG* signal accumulated in protonemal tissues close to buds (G–J and M–P; arrows indicate signal in protonemata), *PpCLV1b::NGG* and *PpRPK2::NGG* signal accumulated mainly in the apical region of buds (Q and R). At two later stages of gametophore development (S–X and Y–J'), all promoters were active in gametophores, although the patterns and intensity of activity varied between reporters and by developmental stage. *PpCLE1::NGG* lines stained most strongly in leaves (S, Y, and E'), *PpCLE2::NGG* lines stained most strongly in leaves and gametophore bases (T, Z, and F'), and *PpCLE7::NGG* lines accumulated stain in rhizoid tips (arrow in U), leaf bases (arrow in A'), and hairs around the apex and the gametophore axis (G'). *PpCLV1a::NGG* lines did not stain intensely at early stages of gametophore development (P and V) but accumulated signal in gametophore axes and leaves at later stages (B' and H'). In contrast, *PpCLV1b::NGG* and *PpRPK2::NGG* lines accumulated signal in gametophore axes and leaves from early stages of development (W and X), and strong signal was detected in branches initiating at gametophore bases (arrows in X, C', and D').

All tissues in (A)–(D') were stained in a solution containing 0.5 mM FeCN for times specified in (A)–(F), and gametophores in (E')–(J') were stained three times longer in a solution containing 2 mM FeCN.

The scale bars in (A)–(F) represent 1 mm, the scale bars in (M)–(R) represent 100 μm, and insets in (G)–(L) indicate position of buds in (M)–(R). The scale bars in (S)–(J') represent 1 mm. See also Methods S1 and Table S4.

in day 10 filamentous tissues (Figure S4). These results were broadly consistent with reports from transcriptome data (Figure S5) [23, 24]. We also constructed *promoter::NLSGFPGUS* (*promoter::NGG*) fusion lines for *PpCLE1*, *PpCLE2*, *PpCLE7*, *PpCLV1a*, *PpCLV1b*, and *PpRPK2* as RT-PCR showed that these 6 genes were upregulated at around the time of gametophore initiation (see Strategy for generation of *promoter::NLSGUSGFP* reporter lines in Methods S1; Figure 2). In 3-week-old spot cultures (Figures 2A–2F), *PpCLE1::NGG*, *PpCLE2::NGG*, *PpCLE7::NGG*, and *PpCLV1a::NGG* lines accumulated local signal in various protonemal cell types around the buds (Figures 2G–2J and 2M–2P). *PpCLV1b::NGG* and *PpRPK2::NGG* lines accumulated signal in buds, and the signal was strongest toward the apex (Figures 2K, 2L, 2Q, and 2R). Whereas all lines accumulated signal in gametophore axes and leaves (Figures 2S–2J'), there was variation in the pattern, timing, and intensity of signal accumulation between lines. Notably, *PpCLE1::NGG*, *PpCLE2::NGG*, *PpCLE7::NGG*, and *PpCLV1a::NGG* signal accumulation in gametophores was delayed with respect to *PpCLV1b::NGG* and *PpRPK2::NGG* lines

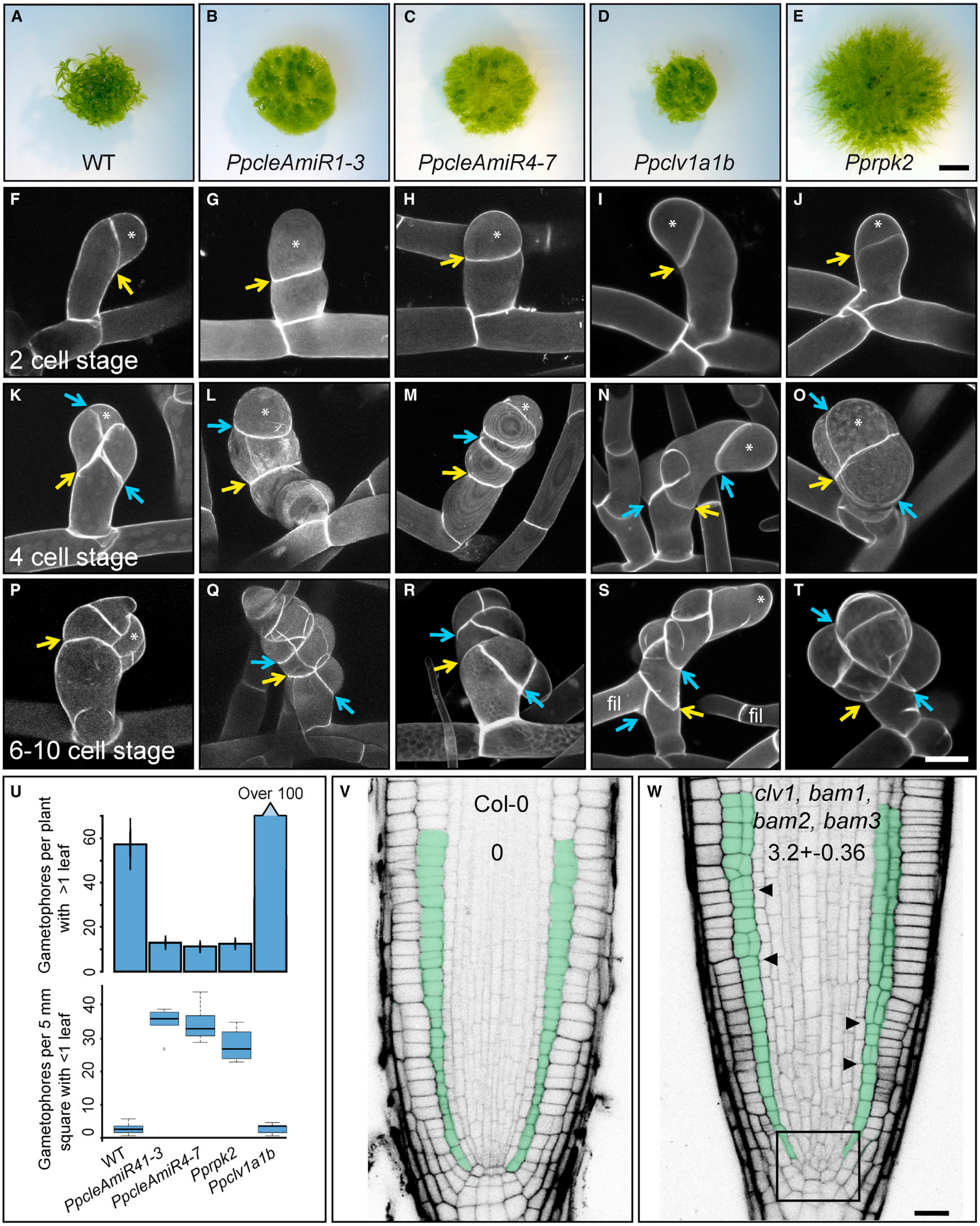

(legend on next page)

(Figures 2M–2X). These beta-glucuronidase (GUS) accumulation patterns suggested highly dynamic foci of expression for *PpCLEs 1*, *2*, and *7* and *PpCLV1a*, *PpCLV1b*, and *PpRPK2* in *Physcomitrella*, prompting us to investigate roles for CLV pathway components in gametophore initiation and development, i.e., during the transition to 3D growth.

Physcomitrella Mutants Lacking CLAVATA Function Have a Defective 2D to 3D Growth Transition

To identify the functions of CLV pathway components, we used artificial microRNAs (AmiRNAs) to silence expression of *PpCLEs 1*, *2*, and *3* and *PpCLEs 4*, 5, 6, and *7* (see Strategy for generating *PpcleAmiR* lines in Methods S1). We used a CRISPR-Cas9 approach to disrupt the function of *PpCLV1* paralogs (see CRISPR/Cas9 strategy for generating *Ppclv1* mutants in Methods S1), and gene targeting was used to abrogate *PpRPK2* function (see Strategy for generating *Pprpk2* KO lines in Methods S1). *PpcleAmiR1-3*, *PpcleAmiR4-7*, *Ppclv1a1b*, and *Pprpk2* lines were able to form dense protonemal tissues and thus had a relatively normal 2D growth phase (Figures 3A–3E). However, all four mutant classes had defective development during the 3D growth phase, with a reduction in the overall number of mature gametophores and defects in gametophore development (Figures 3A–3E and 3U). Further examination revealed many more gametophore buds with 1 or fewer leaves in *PpcleAmiR1-3*, *PpcleAmiR4-7*, and *Pprpk2* mutants than in wild-type (WT) plants (Figure 3U), and *Ppclv1a1b* mutants had many small gametophores arrested at a later stage of development (Figure 3U). These data suggested early defects in gametophore development with potential feedback onto the gametophore initiation process. To determine how WT and mutant phenotypes diverged during development, we imaged gametophore buds at 2-cell, 4-cell, and a later stage of bud development [6] (Figures 3F–3T). Although WT gametophores initiated normally and showed characteristic oblique cell division plane orientations, the plane of the first division was strongly disrupted in *PpcleAmiR1-3* and *PpcleAmiR4-7* mutants, and it was set at a shallow angle relative to the main growth axis (compare Figure 3F to Figures 3G and 3H). A second round of cell division from the apical cell also had misset division planes that were frequently parallel rather than perpendicular to the first division plane, and a subset of gametophores therefore formed finger-like projections in place of gametophores (compare Figure 3K to Figures 3L and 3M). At developmental stages where the tetrahedral shape of the apical cell is normally established [6], mutants also had defects indicating problems with growth and cell fate specification, appearing to reiterate divisions normally characteristic of the first gametophore initial (compare Figure 3P to Figures 3Q and 3R). *Ppclv1a1b* mutant phenotypes diverged from WT after the 2-cell stage, subsequently showing a similar pattern of division to *PpcleAmiR1-3* and *PpcleAmiR4-7* mutants (Figures 3K–3N and 3P–3S), and some cells reverted to filament identity (Figure 3S). *Pprpk2* mutant defects were less severe than *Ppcle* and *Ppclv1a1b* defects at the earliest developmental stages, and at later stages, swollen cell shapes suggested growth defects as well as division plane defects (Figure 3T). The mutant phenotypes above suggest key roles for the *Physcomitrella* CLV pathway in modulating cell division planes, cell fate, growth, and proliferation during the 2D-3D developmental transition. The formation of long projections of swollen cells in *Ppcle* mutants (e.g., Figures 3L and 3M) suggests that gametophore identity is attained normally, as cell swelling is a characteristic of gametophore rather than filament initials. The manifestation of plane orientation defects in the first division suggests that WT and mutant gametophore development diverge at the single-celled stage, after cell fate is specified.

Roles for CLAVATA in Regulating Cell Division Plane Orientation Are Conserved between *Physcomitrella* and *Arabidopsis*

As roles for CLV in cell division plane orientation were previously unreported, we sought to identify conservation of function with *Arabidopsis*. To this end, we examined *Arabidopsis clv1/bam1/bam2/bam3* quadruple mutant meristems, in which the function of the entire *CLV/BAM* gene clade is lost [25]. Whereas division plane orientations are normally stereotypic in root meristems, we detected strongly disordered planes in the stem cell niche and ground tissue layers of *clv1/bam1/bam2/bam3* mutant roots (Figures 3V, 3W, and S6). Thus, a newly identified role for CLV in cell division plane orientation is conserved between *Physcomitrella* and *Arabidopsis*.

Figure 3. The CLV Pathway Regulates Cell Division Plane Orientations during 3D Growth in *Physcomitrella* and *Arabidopsis*

(A–E) Although WT plants (A) developed many normal gametophores, *PpcleAmiR1-3* (B), *PpcleAmiR4-7* (C), *Ppclv1a1b* (D), and *Pprpk2* (E) mutants had no obvious gametophores. The scale bar represents 0.35 cm.

(F–T) *PpcleAmiR1-3*, *PpcleAmiR4-7*, *Ppclv1a1b*, and *Pprpk2* mutants have cell division plane defects at the onset of 3D morphogenesis.

(F–J) The first division of each bud is indicated by a yellow arrow and is set at a strongly oblique angle in WT (F), *Ppclv1a1b* (I), and *Pprpk2* (J) plants, but is weakly oblique in *PpcleAmiR1-3* (G) and *PpcleAmiR4-7* (H) mutants.

(K–O) Whereas (K) the second division (blue arrow) from the apical cell (asterisk) is normally oblique and roughly perpendicular to the first, in *PpcleAmiR1-3* (L), *PpcleAmiR4-7* (M) and *Ppclv1a1b* (N) mutants, it is roughly parallel to the first. *Pprpk2* (O) mutants look normal at this stage.

(P–T) The stereotypical divisions that normally generate the tetrahedral shape of the gametophore apical cell at the 6- to 10-celled stage of development (P) are misset in *PpcleAmiR1-3* (Q), *PpcleAmiR4-7* (R), and *Ppclv1a1b* (S), and *Pprpk2* (T) mutants. The scale bar represents 30 μm.

(U) Bar chart and boxplot showing that gametophore initiation was disrupted in *PpcleAmiR1-3*, *PpcleAmiR4-7*, *PpCLV1a1b*, and *Pprpk2* mutants. The number of gametophores with >1 leaf was counted in 5 WT and mutant plants from a single line representing each mutant class. Gametophore buds with <1 leaf were counted from a 5-mm^2 area in 3 WT and mutant plants from a single line representing each mutant class. ANOVA, Tukey's Honest Significant Difference (HSD) test; $p < 0.005$.

(V and W) Confocal micrographs of WT (Col-0) (V) and *clv1/bam1/bam2/bam3* mutant (W) root tips showing disordered cell division plane orientations in the meristem and ground tissue layers. The box in (W) indicates the meristem, and arrowheads indicate the developmental onset of abnormal periclinal division plane orientations in the cortex layer (shaded green). The scale bar represents 20 μm.

See also Figure S6, Methods S1, and Table S4.

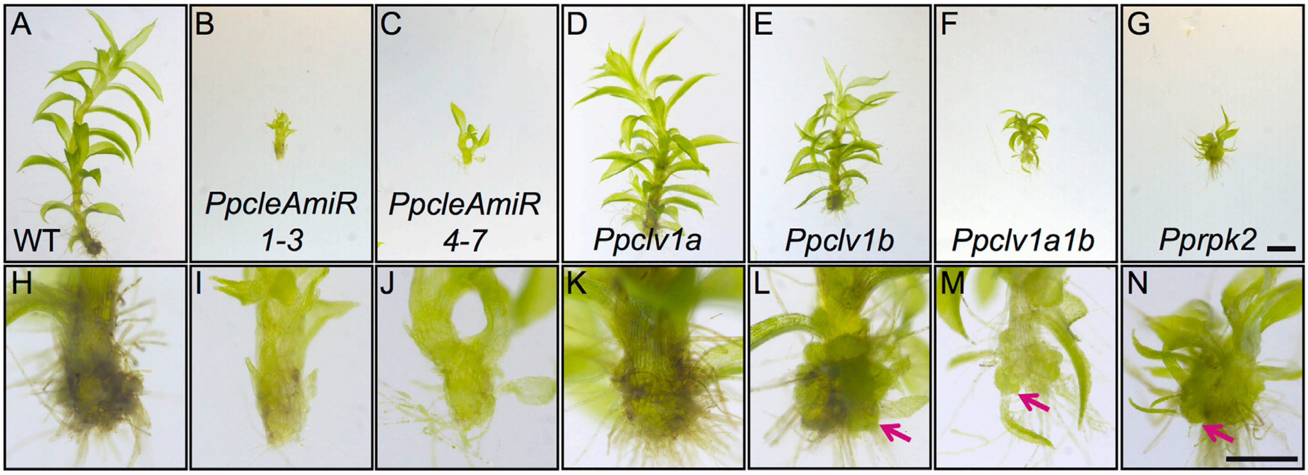

Figure 4. Gametophore Phenotypes in *PpAmiRcle*, *Ppclv1*, and *Pprpk2* Mutants

(A–G) Light micrographs showing height differences between WT (A), *PpcleAmiR1-3* (B), *PpcleAmiR4-7* (C), *Ppclv1a* (D), *Ppclv1b* (E), *Ppclv1a1b* (F), and *Pprpk2* (G) gametophores dissected from 1-month-old plants. The scale bar represents 1 mm.

(H–N) Light micrographs of gametophore bases with arrows showing overproliferation in *Ppclv1b* (L), *Ppclv1a1b* (M), and *Pprpk2* (N) mutants. WT (H), *PpcleAmiR1-3* (I), *PpcleAmiR4-7* (J) and *Ppclv1a* (K) gametophores show no such overproliferation. The scale bar represents 0.5 mm.

Physcomitrella Mutants with Disrupted CLV Function Have Defective Gametophore Development

In *Arabidopsis* and other flowering plants, the CLV pathway is known for its role in maintaining the size of the meristematic stem cell pool [26], and increases in the number of stem cells lead to highly enlarged meristems in both *clv1* and *clv3* (*cle*) mutants. However, *Physcomitrella* does not fit the *Arabidopsis* paradigm of meristem function because the shoot apex comprises a single apical stem cell. The apical cell cleaves merophyte daughter cells in a spiral pattern, and merophytes subsequently divide to generate leaf initials and stem tissues [6]. To investigate whether roles for CLV in regulating stem cell function are conserved between *Physcomitrella* and *Arabidopsis*, we imaged one of the largest gametophores from 1-month-old WT and mutant plants using light and confocal microscopy and found that mutant gametophores were reduced in height and had developmental defects (Figure 4). Although *PpcleAmiR1-3*, *PpcleAmiR4-7*, and *Pprpk2* mutants were most severely reduced in height (Figures 4B, 4C, and 4G), *Ppclv1a* and *Ppclv1b* mutants had milder defects (Figures 4D and 4E). *PpcleAmiR1-3*, *PpcleAmiR4-7*, *Ppclv1a1b*, and *Pprpk2* mutants had defective leaf development, and *Ppclv1b*, *Ppclv1a1b*, and *Pprpk2* mutants also had strong cell fate and/or proliferation defects, developing a callus-like mass at the gametophore base (Figures 4L–4N). Closer inspection revealed that these masses arose by the activity of many ectopic apical cells at the gametophore base (Figure 5). These loss-of-function data suggest that CLV has roles in regulating stem cell function that are conserved between *Physcomitrella* and *Arabidopsis*.

CLE Peptides Can Suppress Cell Proliferation in *Physcomitrella* Gametophores

To further assay conservation in CLV function, we undertook a gain-of-function approach by applying synthetic CLE peptides to growing plants (Figures 6 and S7). After 4 weeks of growth, we found that treatment with a 1-μM concentration of CLE had no appreciable effect on plant spread or the number of gametophores initiating, indicating that protonemal development is normal (Figure S7). However, although solute controls, a randomized peptide and *Arabidopsis* CLE41 (a TDIF CLE) have no appreciable effect on gametophore development, *Arabidopsis* CLV3 and all of the *Physcomitrella* CLEs cause gametophore dwarfing and a strong reduction in leaf size correlating with a reduction in leaf cell number (Figure 6). Although this phenotype superficially resembles the stunted gametophore phenotypes of *PpcleAmiR1-3* and *PpcleAmiR4-7* mutants (Figures 4B and 4C), we found no evidence of developmental arrest or meristematic overproliferation following CLE application and no difference in the number of gametophores initiating was detected following CLE treatment (data not shown). These data show that CLEs act through a conserved signaling module to regulate cell proliferation specifically during the 3D growth phase in *Physcomitrella*.

CLE Peptides Can Act through Receptor Components that Are Conserved between *Physcomitrella* and *Arabidopsis*

Previous studies in *Arabidopsis* have shown that application of CLV3-like, but not TDIF-like, CLEs to roots can arrest meristem function [27]. To assay conservation in peptide function, we germinated *Arabidopsis* seeds on Murashige and Skoog (MS) medium plates containing solute or peptides at a 1 μM concentration. Although solute controls, a randomized peptide, and CLE41 caused no arrest of root development, CLV3 and all of the *Physcomitrella* CLEs caused a significant reduction in root length in *Arabidopsis* resulting from collapse of the root meristem (Figures 7A–7C, 7E, and 7F). *Physcomitrella* CLEs therefore regulate growth and proliferation in a similar manner to CLV3 in *Arabidopsis*. To confirm that PpCLEs can act through a conserved receptor machinery, we used peptide treatment assays on *Arabidopsis* and

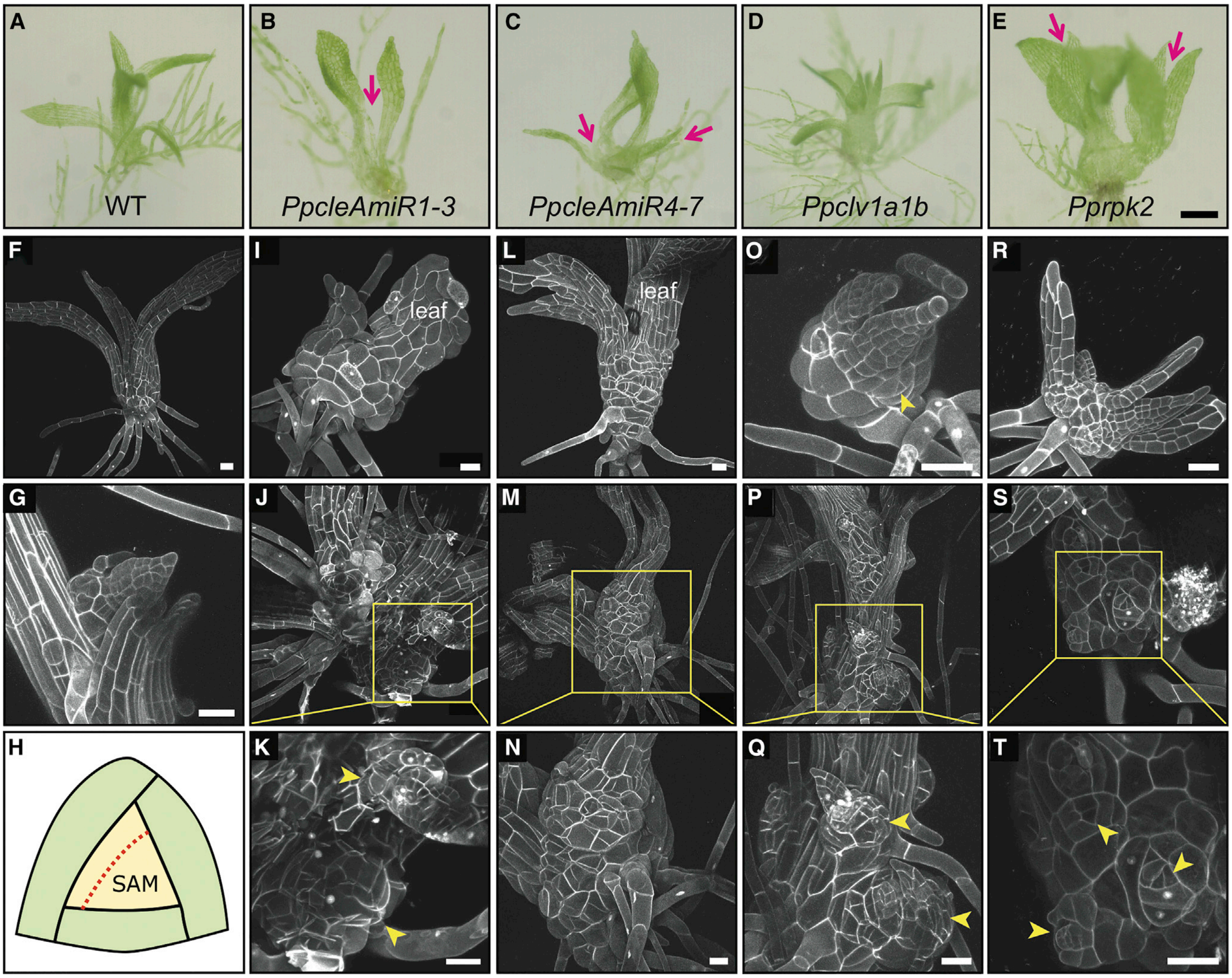

Figure 5. Overproliferation Phenotypes in *PpAmiRcle*, *Ppclv1*, and *Pprpk2* Mutants

(A–E) Light micrographs of mutant gametophore morphology showing that gametophores (B) arrest, (C and E) develop multiple axes (pink arrows), and (C–E) develop swollen bases relative to (A) WT plants. The scale bar represents 200 μm.

(F and G) Confocal micrographs showing (F) overall gametophore morphology and (G) a branch initiating in a leaf axil in WT plants.

(H) Schematic showing *Physcomitrella* gametophore apex organization with an apical cell (pale yellow) and rotating division plane orientations.

(I–T) Confocal micrographs showing (I–K) *PpcleAmiR1-3* mutant gametophore morphologies, with (I) overproliferation at the gametophore base and (J and K) disorganized growth with ectopic meristems.

(L–N) *PpcleAmiR4-7* mutant gametophore morphologies with (L) split leaf phenotypes and (M and N) meristem overproliferation and termination.

(O–Q) *Ppclv1a1b* mutant gametophore morphology (O), with multiple growth axes and multiple meristems at the gametophore base (P and Q).

(R–T) *Pprpk2* mutant gametophore morphology with multiple growth axes (R) and multiple meristems at the gametophore base (S and T).

Yellow arrowheads indicate regions of overproliferation or ectopic meristems. Yellow boxes show regions magnified from (J), (M), (P), and (S) to (K), (N), (Q), and (T). The scale bars represent 50 μm.

Physcomitrella rpk2 mutants (Figure 7). Whereas treatment of WT *Arabidopsis* plants with CLV3-like peptides strongly inhibited root growth, *rpk2* mutants showed less growth inhibition when treated with *Arabidopsis* and *Physcomitrella* peptides (Figures 7A–7C, 7E, and 7F). These data are in line with previously published results showing that RPK2 acts among other receptors to contribute to CLV signaling in *Arabidopsis* [17] and show that *Physcomitrella* CLEs can also act via RPK2 in *Arabidopsis*. To determine whether *Physcomitrella* CLEs act via PpRPK2, we performed similar experiments in WT, *Ppcle*, and *Pprpk2* mutant backgrounds. *Ppcle* mutant gametophores are roughly the same size as *Pprpk2* mutant gametophores, and we reasoned that, if PpCLEs act via PpRPK2, we should detect a response in *Ppcle* mutants, but not *Pprpk2* mutants. As in previous experiments, we found strong inhibition of gametophore development in WT plants (Figure 7D). Potentially due to lack of positional information, treatment of *Ppcle* mutants with CLE peptides did not rescue developmental defects but nevertheless induced a gametophore dwarfing response, consistent with an intact receptor machinery (Figures 7D and 7G–7I). In contrast, *Pprpk2* mutants showed no morphological

response to CLE application, suggesting that PpCLEs act via PpRPK2 in regulating 3D growth (Figures 7D and 7G–7J).

DISCUSSION

How Might CLV Pattern Cell Division Plane Orientation?

We propose that the CLV pathway regulates the 2D to 3D developmental transition in *Physcomitrella* by orienting gametophore cell division planes and regulating growth and fate. How ligands and receptors act together to do this is not yet clear. One possibility is that CLE ligands diffuse to create a concentration gradient that division planes are patterned against. A similar mechanism involving CLEs patterns cambial meristems in *Arabidopsis* [28], where CLE41 is synthesized in the phloem and diffuses to bind PXY receptors in neighboring procambial cells, thereby imparting spatial information for periclinal division [28]. Constitutive or ectopic expression of *CLE41* disrupts this positional information, resulting in disordered cambial division planes [28]. In *Physcomitrella*, similar patterning could be achieved by sub-cellular localization of receptors to create a graded CLV response in bud initials, or at later stages of development, patterning could be provided by receptor expression in different portions of buds.

It is also possible that CLV signaling does not directly modulate cell division planes but that CLV influences cell division planes via hormone signaling, cell geometry, and/or cell mechanics. Auxin signaling and the activity of microtubule-interacting proteins, such as CLIP-associated proteins (CLASPs), are known to specify cell division planes in *Arabidopsis* embryos [29], and auxin signaling modulates the activity of previously identified factors necessary for correct division plane orientation in *Physcomitrella* buds, including *DEK1* and *NOG1* [30, 31]. There appears to be a complex interplay between auxin and cytokinin in *Physcomitrella* [32–34], and several phenotypes suggest that this interplay is disrupted in *Ppcle*, *Ppclv*, and *Pprpk2* mutants. For instance, cell fate and proliferation at the gametophore base are perturbed (Figures 4 and 5) and leaf cell proliferation is perturbed in plants treated with CLEs (Figure 6), and these aspects of development are auxin and cytokinin regulated [33, 34]. Linking CLV signaling to the hormone pathways regulating growth and fate will be important in unravelling mechanisms of cell division plane specification during 3D growth.

CLAVATA-Regulated Stem Cell Function Is an Ancestral Feature of Land Plants

The data we present are important in two evolutionary contexts. First, they show that the CLV pathway originated with land plants and that CLV-regulated stem cell proliferation and function is

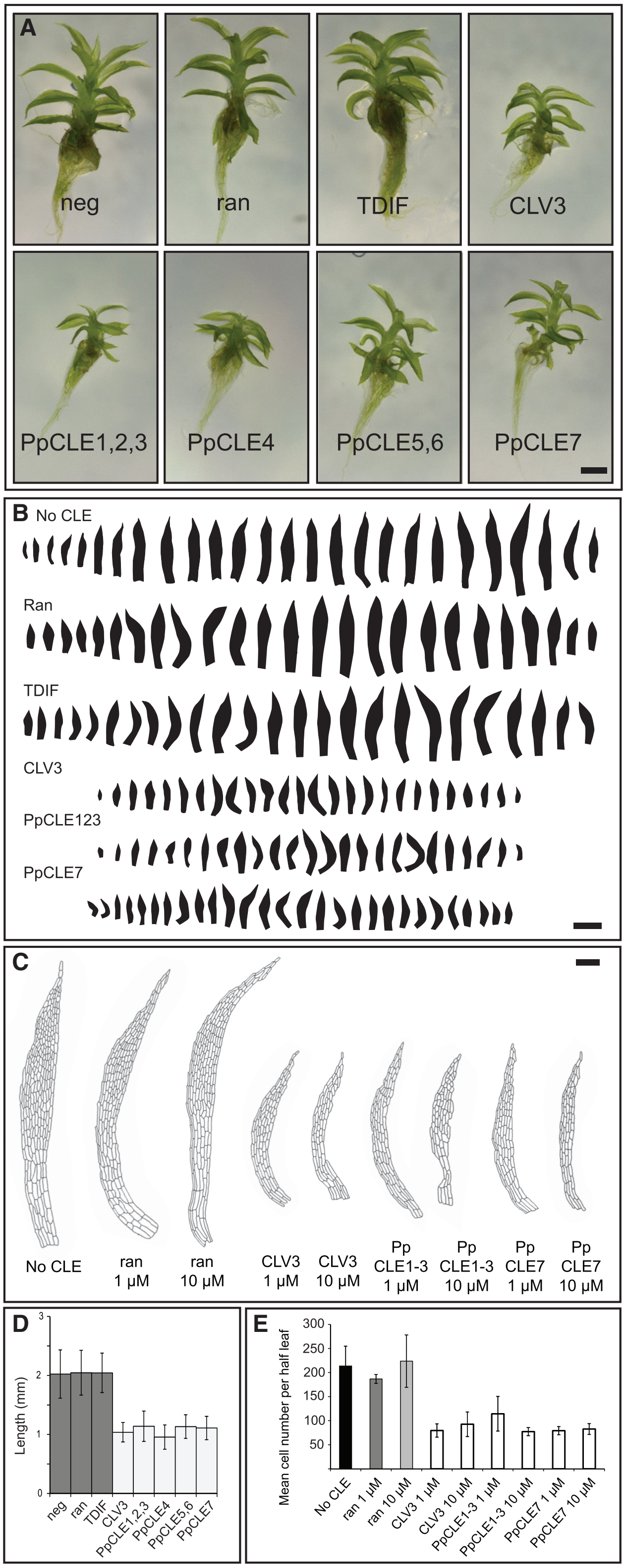

Figure 6. *Physcomitrella* CLEs Suppress Cell Proliferation

(A) Treatment of *Physcomitrella* plants with 1 μM CLV3-like CLEs from *Arabidopsis* and *Physcomitrella*, but not TDIF-like CLEs, causes gametophore and leaf stunting. The scale bar represents 100 μm.

(B) Leaf series from gametophores treated with CLEs expressed during gametophore development. The scale bar represents 1 mm.

(C) Cell outlines of half-leaves in CLE-treated gametophores (leaf 9 was used). The scale bar represents 100 μm.

(D) Height measured from ≥25 gametophores treated with CLEs (n ≥ 25; ANOVA; Tukey's HSD; $p < 0.005$).

(E) Leaf 9 cell numbers in CLE-treated half-leaves (n = 3; ANOVA; Tukey's HSD; $p < 0.05$).

See also Figure S7.

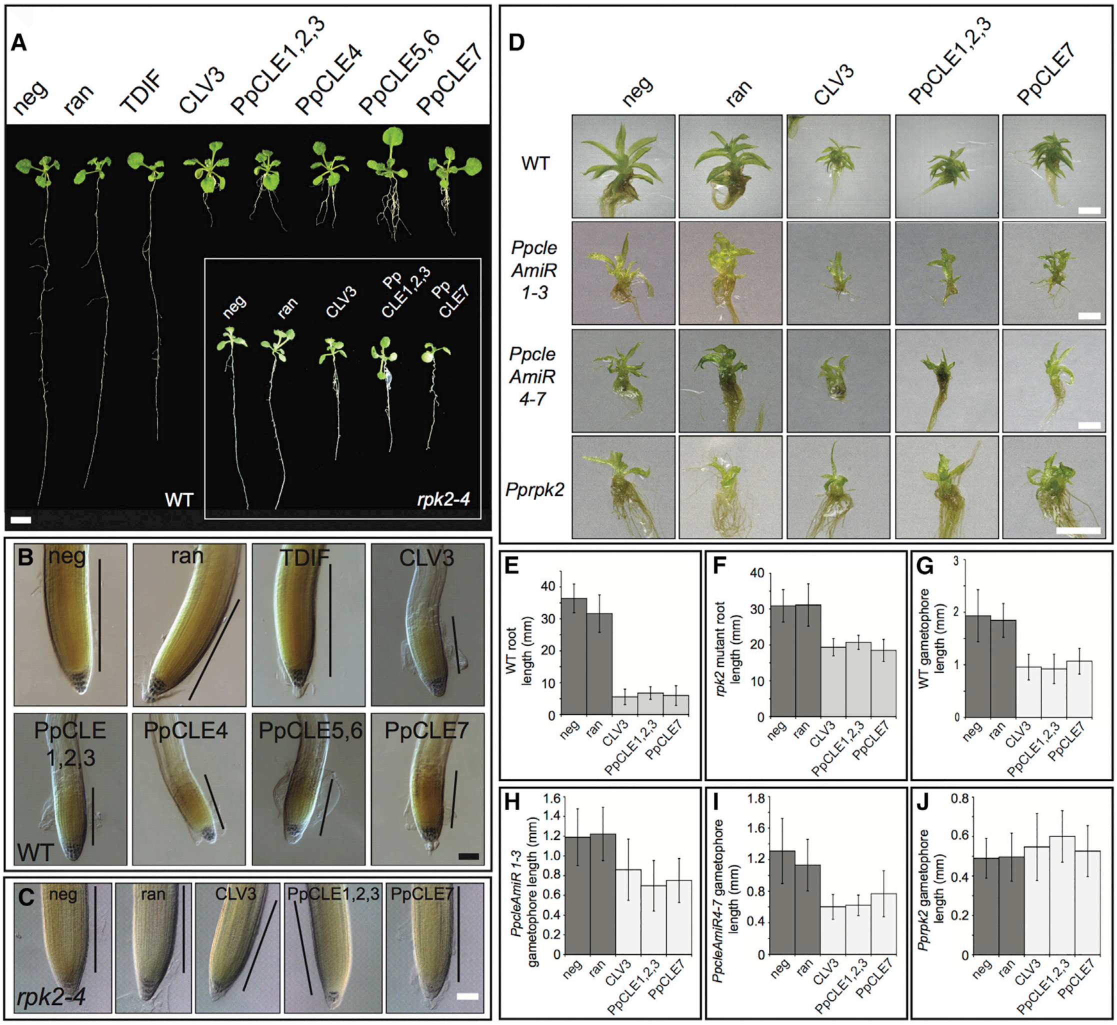

Figure 7. *Physcomitrella* CLE Peptides Act via a Conserved Receptor Machinery

(A and B) Treatment of *Arabidopsis* seedlings with 1 μM CLV3-like CLEs from *Arabidopsis* and *Physcomitrella*, but not TDIF-like CLEs, suppresses root meristem proliferation in WT *Arabidopsis* (n = 30; ANOVA; Tukey's HSD; $p < 0.0005$). The scale bar in (A) represents 1 cm and scale bar in (B) represents 100 μm; black bars in (B) represent approximate position and extent of root meristem.

(C and inset in A) *Arabidopsis rpk2-4* mutants are resistant to treatment with 1 μM CLE peptides; $n \geq 15$; ANOVA; Tukey's HSD; $p < 0.0005$. The scale bar represents 100 μm in (C).

(D) Gametophores in plants treated with 10 μM CLEs are stunted in WT, *PpcleAmiR1-3*, and *PpcleAmiR4-7* mutants, but not *Pprpk2* mutants ($n \geq 20$; ANOVA; Tukey's HSD; $p < 0.0005$). The scale bar represents 100 μm.

(E and F) Quantitative data on root length in WT (E) and *rpk2* mutant plants (F), supporting inferences from images shown in (A).

(G–J) Quantitative data on gametophore length in WT (G), *PpcleAmiR1-3* (H), *PpcleAmiR4-7* (I), and *Pprpk2* (J), mutant plants supporting inferences from images shown in (D).

likely to be an ancestral feature of land plants. The acquired capacity of land plants to orient stem cell divisions in multiple planes enabled diversification by permitting plants to develop upright axes with organs arranged in multiple orientations, a crucial step in shoot evolution [1]. Stem cell division plane defects in *Ppcle* mutants specifically affect the transition to 3D growth and the 3D growth phase, and morphological responses to peptide application are also specific to the 3D growth phase. Thus, in an ancient land plant group, CLV regulates a developmental transition that mirrors an evolutionary transition. The data suggest that CLV was a genetic novelty for a key morphological innovation of land plants.

CLAVATA-Regulated Meristem Functions Originated prior to WOX- and KNOX-Regulated Meristem Functions

Second, the data are important in the context of evolving gene regulatory networks for land plant meristem function. Whereas the first land plant meristems comprised a single gametophytic stem cell, the multicellular sporophyte meristems of vascular plants combine stem cell and more generally proliferative capacities [1]. Class I *KNOX* genes regulate meristematic proliferation in vascular plants [35, 36], but these roles are not shared between bryophytes and vascular plants. Moss *KNOX* (*MKN*) genes are primarily expressed in sporophyte tissues [24, 37], and although loss-of-function *mkn2* mutants have elongation

defects in sporophytes, they have normal gametophytes [37]. *WOX* genes are key regulators of stem cell proliferation in *Arabidopsis* [19]. However, this function was acquired by the recently derived *WUS* gene clade [38, 39], and the downstream pathways regulated by CLV in *Physcomitrella* are likely to be distinct from those in *Arabidopsis* as *Ppwox13L* mutant gametophores develop normally [40]. Thus, class I *KNOX*- and *WOX*-regulated meristem functions were both acquired after the bryophyte-vascular plant divergence. CLV was important in the origin of land plant meristem functions in the gametophyte stage of the life cycle, and we speculate that CLV was recruited to regulate stem cell function in the sporophyte stage of the life cycle prior to the origin of *KNOX*- and *WOX*- regulated meristem functions.

STAR★METHODS

Detailed methods are provided in the online version of this paper and include the following:

- KEY RESOURCES TABLE
- CONTACT FOR REAGENT AND RESOURCE SHARING
- EXPERIMENTAL MODELS AND SUBJECT DETAILS
  - *Arabidopsis* plant growth
  - *Physcomitrella* plant growth
- METHOD DETAILS
  - Sequence retrieval
  - Phylogenetic reconstruction
  - Molecular biology
  - Transgenic line generation and phenotype analyses
- QUANTIFICATION AND STATISTICAL ANALYSIS
- DATA AND SOFTWARE AVAILABILITY

SUPPLEMENTAL INFORMATION

Supplemental Information includes seven figures, four tables, three data files, and supplemental text and is available online at https://doi.org/10.1016/j.cub.2018.05.068.

ACKNOWLEDGMENTS

C.J.H. thanks the Gatsby Charitable Foundation (GAT2962), BBSRC (BB/L02248/1), and Royal Society (UF130563) for funding. J.C. thanks Santander and the Cambridge Overseas Trust for funding his Master's research. Z.N.V. thanks the Gatsby Charitable Foundation for funding. Z.L.N. and A.D.C. thank the NIH (R35GM119614-01) for funding. P.S. and M.W. thank the Swiss National Science Foundation (160004 and 131726 to P.S.), URPP Evolution in Action (to P.S. and M.W.), and Georges und Antoine Claraz-Schenkung (to P.S. and M.W.) for funding. We thank Takashi Ishida for *rpk2* mutant seed. We thank Christophe Dunand, Liam Dolan, and Takayuchi Kohchi for access to *Spirogyra* and *Marchantia* genome data. We thank Tomoaki Nishiyama and Stefan Rensing for searching *Chara* genome data on our behalf. We thank Jon Hughes and Maureen Liu for preliminary expression analyses.

AUTHOR CONTRIBUTIONS

Database Searches, C.D.W., M.W., P.S., and C.J.H.; Phylogenetic Analyses, C.D.W. and C.J.H.; RT-PCR, C.D.W. and C.J.H.; *promoter::NGG* Line Generation and Characterization, Z.N.V., C.D.W., X.Y.W., T.A., Y.K., A.C.C., S.S., and C.J.H.; *AmiRcle1-3* and *AmiRcle4-7* Line Generation and Characterization, C.D.W., J.C., Z.N.V., and C.J.H.; *Ppclv1a1b* Mutant Generation and Characterization, J.C., Z.N.V., A.H.K.R., M.J.S., and C.J.H.; *Pprpk2* Mutant Generation and Characterization, J.C., C.D.W., Z.N.V., X.Y.W., T.A., A.H.K.R., M.J.S., and C.J.H.; Peptide Application Experiments, C.D.W. and C.J.H.; *Arabidopsis clv1/bam1/bam2/bam3* Mutant Analysis, A.D.C. and Z.L.N.; Writing the Manuscript, C.J.H.; Editing the Manuscript, C.J.H., C.D.W., J.C., Z.N.V., T.A., P.S., M.J.S., and A.H.K.R.

DECLARATION OF INTERESTS

The authors declare no competing interests.

Received: January 30, 2018
Revised: May 10, 2018
Accepted: May 23, 2018
Published: July 19, 2018

REFERENCES

1. Harrison, C.J. (2017). Development and genetics in the evolution of land plant body plans. Philos. Trans. R. Soc. Lond. B Biol. Sci. *372*, e20150490.
2. Zimmermann, W. (1952). Main results of the "Telome theory". Palaeobotanist *1*, 456–470.
3. Graham, L.E., Cook, M.E., and Busse, J.S. (2000). The origin of plants: body plan changes contributing to a major evolutionary radiation. Proc. Natl. Acad. Sci. USA *97*, 4535–4540.
4. Wickett, N.J., Mirarab, S., Nguyen, N., Warnow, T., Carpenter, E., Matasci, N., Ayyampalayam, S., Barker, M.S., Burleigh, J.G., Gitzendanner, M.A., et al. (2014). Phylotranscriptomic analysis of the origin and early diversification of land plants. Proc. Natl. Acad. Sci. USA *111*, E4859–E4868.
5. Leliaert, F., Smith, D.R., Moreau, H., Herron, M.D., Verbruggen, H., Delwiche, C.F., and De Clerck, O. (2012). Phylogeny and molecular evolution of the green algae. Crit. Rev. Plant Sci. *31*, 1–46.
6. Harrison, C.J., Roeder, A.H.K., Meyerowitz, E.M., and Langdale, J.A. (2009). Local cues and asymmetric cell divisions underpin body plan transitions in the moss *Physcomitrella patens*. Curr. Biol. *19*, 461–471.
7. Aoyama, T., Hiwatashi, Y., Shigyo, M., Kofuji, R., Kubo, M., Ito, M., and Hasebe, M. (2012). AP2-type transcription factors determine stem cell identity in the moss *Physcomitrella patens*. Development *139*, 3120–3129.
8. Brand, U., Grünewald, M., Hobe, M., and Simon, R. (2002). Regulation of CLV3 expression by two homeobox genes in *Arabidopsis*. Plant Physiol. *129*, 565–575.
9. Reddy, G.V., and Meyerowitz, E.M. (2005). Stem-cell homeostasis and growth dynamics can be uncoupled in the *Arabidopsis* shoot apex. Science *310*, 663–667.
10. Ito, Y., Nakanomyo, I., Motose, H., Iwamoto, K., Sawa, S., Dohmae, N., and Fukuda, H. (2006). Dodeca-CLE peptides as suppressors of plant stem cell differentiation. Science *313*, 842–845.
11. Clark, S.E., Williams, R.W., and Meyerowitz, E.M. (1997). The *CLAVATA1* gene encodes a putative receptor kinase that controls shoot and floral meristem size in *Arabidopsis*. Cell *89*, 575–585.
12. Milani, P., Mirabet, V., Cellier, C., Rozier, F., Hamant, O., Das, P., and Boudaoud, A. (2014). Matching patterns of gene expression to mechanical stiffness at cell resolution through quantitative tandem epifluorescence and nanoindentation. Plant Physiol. *165*, 1399–1408.
13. Schoof, H., Lenhard, M., Haecker, A., Mayer, K.F., Jürgens, G., and Laux, T. (2000). The stem cell population of *Arabidopsis* shoot meristems in maintained by a regulatory loop between the *CLAVATA* and *WUSCHEL* genes. Cell *100*, 635–644.
14. Fletcher, J.C., Brand, U., Running, M.P., Simon, R., and Meyerowitz, E.M. (1999). Signaling of cell fate decisions by CLAVATA3 in *Arabidopsis* shoot meristems. Science *283*, 1911–1914.
15. Nimchuk, Z.L., Tarr, P.T., Ohno, C., Qu, X., and Meyerowitz, E.M. (2011). Plant stem cell signaling involves ligand-dependent trafficking of the CLAVATA1 receptor kinase. Curr. Biol. *21*, 345–352.
16. Ogawa, M., Shinohara, H., Sakagami, Y., and Matsubayashi, Y. (2008). *Arabidopsis* CLV3 peptide directly binds CLV1 ectodomain. Science *319*, 294.

17. Kinoshita, A., Betsuyaku, S., Osakabe, Y., Mizuno, S., Nagawa, S., Stahl, Y., Simon, R., Yamaguchi-Shinozaki, K., Fukuda, H., and Sawa, S. (2010). RPK2 is an essential receptor-like kinase that transmits the CLV3 signal in *Arabidopsis*. Development *137*, 3911–3920.

18. Nimchuk, Z.L. (2017). CLAVATA1 controls distinct signaling outputs that buffer shoot stem cell proliferation through a two-step transcriptional compensation loop. PLoS Genet. *13*, e1006681.

19. Laux, T., Mayer, K.F., Berger, J., and Jürgens, G. (1996). The *WUSCHEL* gene is required for shoot and floral meristem integrity in *Arabidopsis*. Development *122*, 87–96.

20. Yadav, R.K., Perales, M., Gruel, J., Girke, T., Jönsson, H., and Reddy, G.V. (2011). WUSCHEL protein movement mediates stem cell homeostasis in the *Arabidopsis* shoot apex. Genes Dev. *25*, 2025–2030.

21. Cox, C.J., Li, B., Foster, P.G., Embley, T.M., and Civán, P. (2014). Conflicting phylogenies for early land plants are caused by composition biases among synonymous substitutions. Syst. Biol. *63*, 272–279.

22. Bowman, J.L., Kohchi, T., Yamato, K.T., Jenkins, J., Shu, S., Ishizaki, K., Yamaoka, S., Nishihama, R., Nakamura, Y., Berger, F., et al. (2017). Insights into land plant evolution garnered from the *Marchantia polymorpha* genome. Cell *171*, 287–304.e15.

23. Ortiz-Ramírez, C., Hernandez-Coronado, M., Thamm, A., Catarino, B., Wang, M., Dolan, L., Feijó, J.A., and Becker, J.D. (2016). A transcriptome atlas of *Physcomitrella patens* provides insights into the evolution and development of land plants. Mol. Plant *9*, 205–220.

24. Frank, M.H., and Scanlon, M.J. (2015). Cell-specific transcriptomic analyses of three-dimensional shoot development in the moss *Physcomitrella patens*. Plant J. *83*, 743–751.

25. Nimchuk, Z.L., Zhou, Y., Tarr, P.T., Peterson, B.A., and Meyerowitz, E.M. (2015). Plant stem cell maintenance by transcriptional cross-regulation of related receptor kinases. Development *142*, 1043–1049.

26. Somssich, M., Je, B.I., Simon, R., and Jackson, D. (2016). CLAVATA-WUSCHEL signaling in the shoot meristem. Development *143*, 3238–3248.

27. Fiers, M., Golemiec, E., Xu, J., van der Geest, L., Heidstra, R., Stiekema, W., and Liu, C.M. (2005). The 14-amino acid CLV3, CLE19, and CLE40 peptides trigger consumption of the root meristem in *Arabidopsis* through a CLAVATA2-dependent pathway. Plant Cell *17*, 2542–2553.

28. Etchells, J.P., and Turner, S.R. (2010). The PXY-CLE41 receptor ligand pair defines a multifunctional pathway that controls the rate and orientation of vascular cell division. Development *137*, 767–774.

29. Chakrabortty, B., Willemsen, V., de Zeeuw, T., Liao, C.-Y., Weijers, D., Mulder, B., and Scheres, B. (2018). A microtubule-based mechanism predicts cell division orientation in plant embryogenesis. bioRxiv. https://doi.org/10.1101/270793.

30. Perroud, P.F., Demko, V., Johansen, W., Wilson, R.C., Olsen, O.A., and Quatrano, R.S. (2014). Defective Kernel 1 (DEK1) is required for three-dimensional growth in *Physcomitrella patens*. New Phytol. *203*, 794–804.

31. Moody, L.A., Kelly, S., Rabbinowitsch, E., and Langdale, J.A. (2018). Genetic regulation of the 2D to 3D growth transition in the moss *Physcomitrella patens*. Curr. Biol. *28*, 473–478.e5.

32. Ashton, N.W., Grimsley, N.H., and Cove, D.J. (1979). Analysis of gametophytic development in the moss, *Physcomitrella patens*, using auxin and cytokinin resistant mutants. Planta *144*, 427–435.

33. Barker, E.I., and Ashton, N.W. (2013). Heteroblasty in the moss, *Aphanoregma patens* (*Physcomitrella patens*), results from progressive modulation of a single fundamental leaf developmental programme. J. Bryol. *35*, 185–196.

34. Coudert, Y., Palubicki, W., Ljung, K., Novak, O., Leyser, O., and Harrison, C.J. (2015). Three ancient hormonal cues co-ordinate shoot branching in a moss. eLife *4*, e06808.

35. Long, J.A., Moan, E.I., Medford, J.I., and Barton, M.K. (1996). A member of the KNOTTED class of homeodomain proteins encoded by the *STM* gene of *Arabidopsis*. Nature *379*, 66–69.

36. Harrison, C.J., Corley, S.B., Moylan, E.C., Alexander, D.L., Scotland, R.W., and Langdale, J.A. (2005). Independent recruitment of a conserved developmental mechanism during leaf evolution. Nature *434*, 509–514.

37. Sakakibara, K., Nishiyama, T., Deguchi, H., and Hasebe, M. (2008). Class 1 *KNOX* genes are not involved in shoot development in the moss *Physcomitrella patens* but do function in sporophyte development. Evol. Dev. *10*, 555–566.

38. Nardmann, J., and Werr, W. (2012). The invention of *WUS*-like stem cell-promoting functions in plants predates leptosporangiate ferns. Plant Mol. Biol. *78*, 123–134.

39. Dolzblasz, A., Nardmann, J., Clerici, E., Causier, B., van der Graaff, E., Chen, J., Davies, B., Werr, W., and Laux, T. (2016). Stem cell regulation by *Arabidopsis WOX* genes. Mol. Plant *9*, 1028–1039.

40. Sakakibara, K., Reisewitz, P., Aoyama, T., Friedrich, T., Ando, S., Sato, Y., Tamada, Y., Nishiyama, T., Hiwatashi, Y., Kurata, T., et al. (2014). *WOX13*-like genes are required for reprogramming of leaf and protoplast cells into stem cells in the moss *Physcomitrella patens*. Development *141*, 1660–1670.

41. Ishikawa, M., Murata, T., Sato, Y., Nishiyama, T., Hiwatashi, Y., Imai, A., Kimura, M., Sugimoto, N., Akita, A., Oguri, Y., et al. (2011). *Physcomitrella* cyclin-dependent kinase A links cell cycle reactivation to other cellular changes during reprogramming of leaf cells. Plant Cell *23*, 2924–2938.

42. Ossowski, S., Schwab, R., and Weigel, D. (2008). Gene silencing in plants using artificial microRNAs and other small RNAs. Plant J. *53*, 674–690.

43. Hellens, R.P., Edwards, E.A., Leyland, N.R., Bean, S., and Mullineaux, P.M. (2000). pGreen: a versatile and flexible binary Ti vector for *Agrobacterium*-mediated plant transformation. Plant Molecular Biology *42*, 819–832.

44. Eshed, Y., Baum, S.F., Perea, J.V., and Bowman, J.L. (2001). Establishment of polarity in lateral organs of plants. Curr. Biol. *11*, 1251–1260.

45. Hellens, R.P., Edwards, E.A., Leyland, N.R., Bean, S., and Mullineaux, P.M. (2000). pGreen: a versatile and flexible binary Ti vector for *Agrobacterium*-mediated plant transformation. Plant Mol. Biol. *42*, 819–832.

46. Lopez-Obando, M., Hoffmann, B., Géry, C., Guyon-Debast, A., Téoulé, E., Rameau, C., Bonhomme, S., and Nogué, F. (2016). Simple and efficient targeting of multiple genes through CRISPR-Cas9 in *Physcomitrella patens*. G3 (Bethesda) *6*, 3647–3653.

47. Schaefer, D.G., Delacote, F., Charlot, F., Vrielynck, N., Guyon-Debast, A., Le Guin, S., Neuhaus, J.M., Doutriaux, M.P., and Nogué, F. (2010). RAD51 loss of function abolishes gene targeting and de-represses illegitimate integration in the moss *Physcomitrella patens*. DNA Repair (Amst.) *9*, 526–533.

48. Moody, L.A., Kelly, S., Coudert, Y., Nimchuk, Z.L., Harrison, C.J., and Langdale, J.A. (2018). Somatic hybridization provides segregating populations for the identification of causative mutations in sterile mutants of the moss *Physcomitrella patens*. New Phytol. *218*, 1270–1277.

49. Altschul, S.F., Gish, W., Miller, W., Myers, E.W., and Lipman, D.J. (1990). Basic local alignment search tool. J. Mol. Biol. *215*, 403–410.

50. Petersen, T.N., Brunak, S., von Heijne, G., and Nielsen, H. (2011). SignalP 4.0: discriminating signal peptides from transmembrane regions. Nat. Methods *8*, 785–786.

51. Kumar, S., Stecher, G., and Tamura, K. (2016). MEGA7: Molecular Evolutionary Genetics Analysis Version 7.0 for Bigger Datasets. Mol. Biol. Evol. *33*, 1870–1874.

52. Haeussler, M., Schönig, K., Eckert, H., Eschstruth, A., Mianné, J., Renaud, J.-B., Schneider-Maunoury, S., Shkumatava, A., Teboul, L., Kent, J., et al. (2016). Evaluation of off-target and on-target scoring algorithms and integration into the guide RNA selection tool CRISPOR. Genome Biol. *17*, 148.

53. Murashige, T., and Skoog, F. (1962). A revised medium for rapid growth and bio assays with tobacco tissue cultures. Physiol. Plant. *15*, 473–497.

54. Ashton, N.W., and Cove, D.J. (1977). The isolation and preliminary characterisation of auxotrophic and analogue resistant mutants of the moss, *Physcomitrella patens*. Mol. Gen. Genet. *154*, 87–95.

55. Kinoshita, A., Nakamura, Y., Sasaki, E., Kyozuka, J., Fukuda, H., and Sawa, S. (2007). Gain-of-function phenotypes of chemically synthetic CLAVATA3/ESR-related (CLE) peptides in *Arabidopsis thaliana* and *Oryza sativa*. Plant Cell Physiol. *48*, 1821–1825.

56. Miwa, H., Tamaki, T., Fukuda, H., and Sawa, S. (2009). Evolution of CLE signaling: origins of the CLV1 and SOL2/CRN receptor diversity. Plant Signaling and Behaviour *4*, 477–481.

57. Mortier, V., Den Herder, G., Whitford, R., Van de Velde, W., Rombauts, S., D'Haeseleer, K., Holsters, M., and Goormachtig, S. (2010). CLE peptides control *Medicago truncatula* nodulation locally and systemically. Plant Physiol. *153*, 222–237.

58. Schmutz, J., Cannon, S.B., Schlueter, J., Ma, J., Mitros, T., Nelson, W., Hyten, D.L., Song, Q., Thelen, J.J., Cheng, J., et al. (2010). Genome sequence of the palaeopolyploid soybean. Nature *463*, 178–183.

59. Strabala, T.J., Phillips, L., West, M., and Stanbra, L. (2014). Bioinformatic and phylogenetic analysis of the CLAVATA3/EMBRYO-SURROUNDING REGION (CLE) and the CLE-LIKE signal peptide genes in the Pinophyta. BMC Plant Biol. *14*, 47.

60. Rensing, S.A., Lang, D., Zimmer, A.D., Terry, A., Salamov, A., Shapiro, H., Nishiyama, T., Perroud, P.F., Lindquist, E.A., Kamisugi, Y., et al. (2008). The *Physcomitrella* genome reveals evolutionary insights into the conquest of land by plants. Science *319*, 64–69.

61. Rojo, E., Sharma, V.K., Kovaleva, V., Raikhel, N.V., and Fletcher, J.C. (2002). CLV3 is localized to the extracellular space, where it activates the *Arabidopsis* CLAVATA stem cell signaling pathway. Plant Cell *14*, 969–977.

62. Nielsen, H., Engelbrecht, J., Brunak, S., and von Heijne, G. (1997). Identification of prokaryotic and eukaryotic signal peptides and prediction of their cleavage sites. Protein Eng. *10*, 1–6.

63. Goad, D.M., Zhu, C., and Kellogg, E.A. (2017). Comprehensive identification and clustering of *CLV3/ESR*-related (CLE) genes in plants finds groups with potentially shared function. New Phytol. *216*, 605–616.

64. Lang, D., Ullrich, K.K., Murat, F., Fuchs, J., Jenkins, J., Haas, F.B., Piednoel, M., Gundlach, H., Van Bel, M., Meyberg, R., et al. (2018). The Physcomitrella patens chromosome-scale assembly reveals moss genome structure and evolution. Plant J. *93*, 515–533.

65. Oelkers, K., Goffard, N., Weiller, G.F., Gresshoff, P.M., Mathesius, U., and Frickey, T. (2008). Bioinformatic analysis of the CLE signaling peptide family. BMC Plant Biol. *8*, 1.

66. Ran, F.A., Hsu, P.D., Wright, J., Agarwala, V., Scott, D.A., and Zhang, F. (2013). Genome engineering using the CRISPR-Cas9 system. Nat. Protoc. *8*, 2281–2308.

67. Schaefer, D., Zryd, J.P., Knight, C.D., and Cove, D.J. (1991). Stable transformation of the moss *Physcomitrella patens*. Mol. Gen. Genet. *226*, 418–424.

68. Abràmoff, M.D., Magalhães, P.J., and Ram, S.J. (2004). Image processing with ImageJ. Biophoton. Int. *11*, 36–42.

69. Willemsen, V., Wolkenfelt, H., de Vrieze, G., Weisbeek, P., and Scheres, B. (1998). The *HOBBIT* gene is required for formation of the root meristem in the *Arabidopsis* embryo. Development *125*, 521–531.

STAR★METHODS

KEY RESOURCES TABLE

REAGENT or RESOURCE	SOURCE	IDENTIFIER
Bacterial and Virus Strains		
E. coli strain DH5α	Widely distributed	N/A
E. coli strain DB3.1	Widely distributed	N/A
E. coli strain DH10B	Widely distributed	N/A
Chemicals, Peptides, and Recombinant Proteins		
Taq polymerase	Widely available	N/A
Phusion High-Fidelity DNA polymerase	ThermoFisher	Cat#F530S
Novagen KOD Hot Start polymerase	Sigma-Aldrich	Cat#71086
Superscript II reverse transcriptase	ThermoFisher	Cat#18064022
Restriction enzymes for cloning	New England Biolabs	N/A
DNase	Fermentas	Cat#EN0525
MS medium	Melford	Cat#M0221
Plant agar	Duchefa	Cat#P1001
Driselase (basidiomycetes sp.)	Sigma-Aldrich	Cat#8037
Polyethylene glycol (PEG) 6000	Sigma-Aldrich	Cat#81255
G418 disulphate	Melford	Cat#G0175
Hygromycin B	Melford	Cat#H7502
Blasticidin S	Melford	Cat#B1220
α-32P dCTP	GE Healthcare	Cat#PB10205
X-GlcA	Melford	Cat#MB1021
Propidium iodide	Sigma-Aldrich	Cat#P4864
Synthetic CLE peptides (95% purity)	Genecust	N/A
Lugol's stain	Fisher Scientific	Cat#12801823
Critical Commercial Assays		
RNeasy RNA extraction kit	QIAGEN	Cat#74104
Plasmid Plus Midi kit	QIAGEN	Cat#12943
Amersham Rediprime II DNA labeling kit	GE Healthcare	Cat#RPN1633
Dig-High Prime DNA labeling and detection starter kit II	Sigma-Aldrich	Cat#11585614910
Dig Easy Hyb	Sigma-Aldrich	Cat#11585762001
Experimental Models: Organisms/Strains		
Physcomitrella patens Gransden	Widely available	N/A
PpCLE1::NGG line	This study	N/A
PpCLE2::NGG line	This study	N/A
PpCLE7::NGG line	This study	N/A
PpCLV1a::NGG line	This study	N/A
PpCLV1b::NGG line	This study	N/A
PpRPK2::NGG line	This study	N/A
PpcleamiR1-3 mutant	This study	N/A
PpcleamiR4-7 mutant	This study	N/A
Ppclv1a mutant	This study	N/A
Ppclv1b mutant	This study	N/A
Ppclv1ab double mutant	This study	N/A
Pprpk2 mutant	This study	N/A

(*Continued on next page*)

Continued

REAGENT or RESOURCE	SOURCE	IDENTIFIER
Arabidopsis thaliana Col-0	Widely available	N/A
Arabidopsis thaliana rpk2-4 mutant	[17]	N/A
Arabidopsis thaliana clv1,bam1,bam2,bam3 mutant	[25]	N/A
Oligonucleotides		
A list of oligonucleotides is given in Table S4	N/A	N/A
Recombinant DNA		
PIG1NGGII construct	[41]	N/A
PpCLE1::NGG construct	This study	N/A
PpCLE2::NGG construct (NptII)	This study	GenBank: MH310732, MH310732
PpCLE7::NGG construct	This study	N/A
PpCLV1a::NGG construct	This study	N/A
PpCLV1b::NGG construct	This study	N/A
PpRPK2::NGG construct (AphIV)	This study	GenBank: MH310733
pRS300	[42]	N/A
pGREEN (Hyg)	[43]	N/A
pGREEN (Kan)	[43]	N/A
pBJ36	[44]	N/A
pBRACT211	[45]	N/A
pJH125	This study	N/A
pJH131	This study	N/A
PpcleAmiR1-3 construct	This study	GenBank: MH310734
PpcleamiR4-7 construct	This study	GenBank: MH310735
U3::Ppclv1a sgRNA5 construct	This study	GenBank: MH310736
U3::Ppclv1a sgRNA7 construct	This study	GenBank: MH310737
U6::Ppclv1b sgRNA construct	This study	GenBank: MH310738
pACT::Cas9 construct	[46]	N/A
pNRF	[47]	N/A
pBHRF108	[48]	N/A
pDONR2.1	Invitrogen	N/A
pGEMT-EASY	Promega	Cat#A1360
Software and Algorithms		
tBLASTn	[49]	N/A
SignalP	[50]	v4.0
MEGA	[51]	v7.0.26
Figtree	http://tree.bio.ed.ac.uk/software/figtree/	v1.4.3
AmiR design software	http://wmd3.weigelworld.org/cgi-bin/webapp.cgi	N/A
CRISPR design software	[52]	http://crispor.tefor.net/
ImageJ	http://imagej.net/Welcome	V1.4.8
Adobe Photoshop	Adobe	N/A
Adobe Illustrator	Adobe	N/A

CONTACT FOR REAGENT AND RESOURCE SHARING

Further information and requests for resources and reagents should be directed to and will be fulfilled by the Lead Contact, Jill Harrison (jill.harrison@bristol.ac.uk). Please note that the transfer of transgenic materials will be subject to MTA and any relevant import permits.

EXPERIMENTAL MODELS AND SUBJECT DETAILS

Arabidopsis plant growth

Columbia (Col-0), *rpk2-4* (*cli*) or *clv1/bam1/bam2/bam3* mutants [17, 25] were used for *Arabidopsis* experiments. Homozygous *rpk2-4* mutants were confirmed using a BamHI dCAPs screen with a PCR fragment amplified using primers AtRPK2-BamHIF and AtRPK2-BamHIR (see primer list). Seeds were surface sterilized in 5% (v/v) sodium hypochlorite for 10 min and washed three times with sterile de-ionised water. They were then stratified at 4°C in darkness for 48 hr and sown on 0.5 X MS plates containing 0.8% agar [53]. Plants were grown vertically for 7 days at 25°C in a 16 hr light/ 8 hr dark cycle prior to observation (*rpk2* experiments) or at 22°C under continuous light (*clv1/bam1/bam2/bam3* experiments).

Physcomitrella plant growth

The Gransden strain of *Physcomitrella patens* [54] was used for all experiments. Plants were grown in sterile culture on BCDAT plates at 23°C in continuous light at 30-50 μmols^{-1} in Sanyo MLR-351 growth cabinets. BCDAT medium comprises 250mg/L $MgSO_4.7H_2O$, 250mg/L KH_2PO_4 (pH6.5), 1010mg/L KNO_3, 12.5mg/L, $FeSO_4.7H_2O$, 0.001% Trace Element Solution (0.614mg/L H_3BO_3, 0.055mg/L $AlK(SO_4)2.12H_2O$, 0.055mg/L $CuSO_4.5H_2O$, 0.028mg/L KBr, 0.028mg/L LiCl, 0.389mg/L $MnCl_2.4H_2O$, 0.055mg/L $CoCl_2.6H_2O$, 0.055mg/L $ZnSO_4.7H_2O$, 0.028mg/L KI and 0.028mg/L $SnCl_2.2H_2O$), 0.92 g/L $C_4H_{12}N_2O_6$ and 8g/L agar with $CaCl_2$ added to a 1mM concentration after autoclaving. Protonemal cultures for transformation were grown on BCDAT plates overlaid with autoclaved cellophane disks and molecular and phenotypic analyses were undertaken using 1 mm spot cultures unless otherwise stated.

METHOD DETAILS

Sequence retrieval

CLE *genes*

Previously described *Arabidopsis thaliana* and *Oryza sativa CLE* sequences were respectively retrieved from TAIR and RAP-DB [55]. *Selaginella moelendorffii* [56], *Glycine max* [57, 58] and *Picea abies* [59] *CLE*s were retrieved from NCBI. To extend taxon sampling within land plants and identify previously unknown *CLE*s, the CLE domains of *Arabidopsis thaliana* CLV3 and CLE41 were used as tBLASTn queries with an e-value cutoff of e^{-100} to screen transcriptome or draft genome assemblies of a basal angiosperm (*Amborella trichopoda*), a fern (*Diplazium wichurae*), a hornwort (*Anthoceros agrestis*), a moss (*Physcomitrella patens* v1.6 [60]) and a liverwort (*Marchantia polymorpha*). Positive hits were used in reciprocal BLASTs until no new sequences were retrieved. All sequences retrieved were checked for the presence of a signal peptide [61] using SignalP [50, 62]. Newly identified CLE sequences were named with a two-letter prefix denoting the genus and species and numbered (Table S1). A recent cluster analysis [63] succeeded our analyses with slight variation in CLE numbers between species for reasons explained in [63]. An updated version of the *Physcomitrella* genome (v 3.1 [64]) also succeeded our analyses, and this includes two further *PpCLE*s that encode the same CLE motif as PpCLEs 1, 2 and 3. While these were not included in this study, V3 gene IDs including *PpCLE8* and *PpCLE9* are listed in Table S3. Transcriptomes and draft or complete genomes of charophyte (*Coleochaete nitellarum, Spirogyra* sp., *Chara braunii*) and chlorophyte algae (*Ulva linza, Chlamydomonas reinhardtii*, *Volvox carteri*, *Ostreoscoccus tauri* and *Chlorella vulgaris*) were also searched but no *CLE*s were found. A previously annotated *Chlamydomonas reinhardtii CLE* [65] was re-analyzed and discarded due to lack of similarity to other *CLE*s and a premature in-frame stop codon. A full list of taxa and databases searched is given in Table S2.

CLV1/RPK2 *genes*

Arabidopsis CLV1 and RPK2 sequences were used to query the databases listed above using tBLASTn searches with an e-value cutoff of e^{-1000}. As the LRR-Receptor kinase family is large, only sequences that retrieved *CLV1* or *RPK2* as a top hit in reciprocal BLASTs to *Arabidopsis* were used in further analyses. Newly identified *CLV1*-like and *RPK2*-like genes were named with a two-letter prefix denoting the genus and species and given an alphabetical epithet (Table S1). A list of taxa searched is given in Table S2.

Phylogenetic reconstruction

To infer CLE relationships, the conserved 12 amino acid CLE motif from 193 *CLE*s was used in neighbor joining reconstructions compiled with the JTT model in MEGA7.0.26 [51] (Figure S1; Data S1). This approach was taken because there is little conservation in CLE structure outside the CLE motif and so few characters can only yield limited phylogenetic signal (see [63]). To infer CLV/BAM relationships, 525 conserved amino acid residues from 36 genes were used in maximum likelihood reconstructions with the JTT model in MEGA7.0.26 [51] (Figure S2; Data S2). To infer RPK2 relationships, 782 conserved amino acid residues from 18 genes were used in maximum likelihood reconstructions with the JTT model in MEGA7.0.26 [51] (Figure S3; Data S3). For all analyses 100 bootstrap replicates were performed and support values over 50% (CLE tree) or 70% (CLV1/BAM and RPK2 trees) are represented above the branches.

Molecular biology

RT-PCR

Total RNA was isolated from 4 day-old protonemal cultures and 10, 21 or 28 day old spot cultures using the QIAGEN RNeasy method. RNA was DNase treated prior to reverse transcription with SuperScript II following manufacturer's guidelines. Semiquantitative

RT-PCR was undertaken using *UBIQUITIN* (Pp1s56_52V6.1) as a loading control. Where possible, primers were designed to span introns to detect genomic contamination, and sequences are listed in Table S4.

Genomic DNA extraction

Genomic DNA was extracted from protonemal cultures using a CTAB (Hexadecyltrimethylammonium bromide) protocol. Snap-frozen tissue was ground in liquid nitrogen and transferred to tubes containing prewarmed extraction buffer (2% CTAB, 1.4 M NaCl, 100 mM Tris pH8.0, 20 mM EDTA pH8.0, 2% PVP and 1 mg/mL RNaseA), with no more than 100 mg of tissue per mL of buffer. Samples were incubated for 10 min at 65°C and an equal volume of 24:1 chloroform:isoamyl alcohol was added and mixed with each sample to form an emulsion. The tubes were centrifuged at high speed (> 10,000 rpm) for 10 min, and the aqueous phase was transferred to a fresh tube prior to DNA precipitation with an equal volume of isopropanol and repeated centrifugation. DNA was washed with 70% ethanol and dissolved in water, 10 mM Tris pH 8.0 or 10 mM Tris pH 8.0 with 1 mM Na_2EDTA.

Generation of promoter::NGG constructs

Promoter sequences from *PpCLE1* (2.1 kbp), *PpCLE2* (2.1 kbp), *PpCLE7* (2 kbp), *PpCLV1a* (2 kbp), *PpCLV1b* (2.8 kbp) and *PpRPK2* (1.4 kbp) were PCR amplified using a proof-reading Taq polymerase and primers listed in Table S4 and cloned directly or via pGEMT Easy into the SmaI site of PIG1NGGII [41] or derivatives with alternative selection cassettes and sequenced prior to linearization and transformation as illustrated in Methods S1.

Generation of AmiR constructs

To generate *PpcleAmiR1-3* and *PpcleAmiR4-7* constructs, resistance cassettes from pGREEN [45] were first inserted into a blunt-ended HindII site of pBJ36 [44]. A soybean *UBIQUITIN* promoter from pBRACT211 [45] was inserted into the SmaI site to drive AmiRNA expression and the resultant plasmids were named pJH125 (KanR) and pJH131 (HygR). AmiRNAs were designed according to [42], generated by degenerate PCR using a proof-reading Taq polymerase and the pRS300 plasmid as a template, cloned into pGEMT-EASY and transferred as XmaI/BamHI fragments into pJH125 or pJH131. Silencing constructs were checked by sequencing and digested with SacI for transformation as illustrated in Methods S1.

Generation of CRISPR constructs

Small cassettes containing two BsaI restriction sites and sgRNAs [66] driven by the *Physcomitrella* U3 or U6 promoter and flanked by *attB* sites were synthesized and cloned into pDONR201. sgRNA sequences were selected and screened for off target hits in the *Physcomitrella* V3 genome using http://crispor.tefor.net/. To clone guide RNAs into expression cassettes, two primers consisting of guide sequences with overhangs for U3 and U6 promoters were annealed and ligated into U3 or U6 expression vectors pre-digested with BsaI. Constructs were checked by sequencing and co-transformed with *pACT::Cas9* [46] to engineer mutants as illustrated in Methods S1.

Generation of RPK2 KO construct

5′ and 3′ flanking regions were PCR amplified with a proof-reading Taq polymerase and cloned sequentially into pGEMT-EASY using primers listed in Table S4. The resultant plasmid was digested with PmeI and AscI, and the AphIV cassette from pBHRF-108 [48] was ligated between *PpRPK2* flanking regions. This plasmid was checked by sequencing and linearized for transformation as illustrated in Methods S1.

Transgenic line generation and phenotype analyses

Moss transformation and line authentication

For gene targeting and AmiR approaches, 10-20 μg of plasmid DNA was isolated using the QIAGEN Plasmid Plus Midi system and linearized as illustrated in Methods S1. For CRISPR approaches, 5-7 μg of Cas9 and pNRF, and 2-3 μg of each gRNA-expressing construct were purified and pooled for transformation [46] at a concentration of at least 1 μg per μL. All solutions for the transformation procedure were prepared prior to commencing transformation [67]. First, a polyethylene glycol (PEG) solution was prepared by adding 10 mL of mannitol/$CaNO_3$ solution (8% mannitol, 0.1 M $Ca(NO_3)$, 10 mM Tris pH7.2) to 2 g of molten PEG 6000, and the tube containing the solution was left in a water bath at 45°C. To isolate protoplasts, homogoenous protonemal cultures were grown for 5 days to a week post passage. A 1% driselase solution was prepared in 25 mL 8% mannitol, and the supernatant was removed and filter sterilized into to a clean 50 mL falcon tube following centrifugation. Tissue from 4-6 plates was transferred into the driselase solution and the tissue suspension was left for 30-40 min with intermittent mixing to allow cell wall digestion. The mixture was then transferred into a fresh tube through a 50 μm filter to remove cell and cell wall debris. Protoplasts were sedimented by centrifugation for 3 min at 120 g, resuspended and washed three times in 10 mL of 8.0% mannitol prior to counting with a hematocytometer. Protoplasts were then sedimented and resuspended to a density of 1.2×10^6 per mL in MMM solution (0.5 M mannitol, 0.15 M $MgCl_2$ and 0.1% MES pH5.6). 300 μL aliquots of protoplasts were dispensed into falcon tubes prior to addition of DNA and 300 μL PEG solution, and cells were then heat shocked for 5 min at 45°C. Transformation mixtures were progressively diluted with 1 mL of 8% mannitol solution and washed. Protoplasts were then sedimented by centrifugation as above and washed four more times. After the final wash and spin, protoplasts were resuspended in 5 mL liquid BCD medium (constituents as specified above but without ammonium tartrate or agar) with 8% mannitol, 10 mM $CaCl_2$ and 0.5% glucose, wrapped in aluminum foil and left at 23°C overnight. The next day, the protoplast suspension was plated onto BCDAT plates overlain with cellophane and containing 8% mannitol and 5 g/L glucose, using c.1 mL per plate. Plants were grown under standard conditions until regenerants comprised 10-20 cells. Cellophane discs were then transferred onto BCDAT plates containing antibiotics for selection (25 μg/mL Hyg, 50 μg/mL G418, 100 μg/mL BSD). Plants were grown for 2 weeks on selection plates prior to transfer onto BCDAT plates lacking antibiotic for 2 weeks and then back on to selection

plates for a further 2 weeks. All lines were screened by PCR, RT-PCR, Southern analysis or sequencing as illustrated in Methods S1. PCR conditions were standard and primer sequences are listed in Table S4.

Southern hybridization

For *PpcleAmiR* Southerns, 10-15 μg genomic DNA was digested with EcoRV and fractionated in 0.8% agarose by gel electrophoresis. DNA in each gel was depurinated with 0.2 M HCl for 20 min and denatured with 0.4 M NaOH for 20 min prior to neutralization for 20 min in a solution containing 3 M NaCl and 1 M Tris pH 7.5. Gels were inverted onto a Whatman paper wick inserted into a bath of 20 X SSC solution, and DNA was transferred onto a nitrocellulose membrane by overnight Southern blotting. DNA was UV cross-linked to the membrane and the membrane was rinsed in water prior to immersion in pre-hybridization solution (3 X SSC, 1% SDS, 0.1% sodium pyrophosphate, 5 X Denhardt's and 200 μg per mL sheared salmon sperm DNA). The probe template was excised with EcoRV and BamHI from the *PpcleAmiR1-3* construct and the probe was synthesized using an Amersham Rediprime II DNA labeling kit as per manufacturer's instructions. Hybridization was undertaken in a 3 X SSC buffer at 58°C and this was followed by two 20 min washes at 58°C in 3 X SSC and 2 X SSC buffers respectively. Membranes were wrapped in Saran Wrap and used to expose X-ray film, and film was then developed using a film processor. For *promoter::NGG* and *Pprpk2* Southerns, 2.5-3 μg genomic DNA was digested as illustrated in Methods S1. Probe templates comprising *PIG1* flanking sequence, *PpRPK2* coding sequence or a hygromycin resistance cassette were PCR amplified and labeled using the Roche DIG High Prime system. Hybridization was undertaken overnight at 42°C using the Roche DIG Easy Hyb system. Washing and detection were performed using the manufacturer's protocol from the Roche DIG High Prime DNA labeling and Detection Starter kit II.

Physcomitrella *plant imaging*

To assess whole plant and gametophore phenotypes, 4 to 5 week-old spot cultures were imaged using a Keyence VHX-1000E digital microscope with a 20-50 X or 50-200 X objective. To analyze bud phenotypes, confocal imaging was undertaken on tissue stained with 0.5 mg/ml propidium iodide using a Leica TCS SP5 microscope with excitation from the 488 or 514 laser line and emission collected at 600-650 nm or using a Zeiss 710 LSM with excitation from a 514 laser line and emission collected at 566-650 nm. To analyze leaf phenotypes, leaves were removed form gametophores, arranged in heteroblastic series, cleared in 1% chloral hydrate overnight, washed in deionised water and treated with 2M NaOH for 2 hr. They were then washed with water and stained with 0.05% toluidine blue for 2 min before destaining for 10 min in water. The stained leaves were then mounted on a slide under a coverslip and imaged to visualize cell outlines. Adobe Illustrator was used to trace leaf outlines to produce silhouettes for illustration purposes (Figure 6). Quantitative analyses of leaf size were performed using ImageJ, and cell numbers were evaluated using the 'analyze particles' option [68]. Leaf size comparisons were undertaken using leaves from the same point in the heteroblastic leaf series [33] as stipulated in figure legends.

Arabidopsis *plant imaging*

Root length was scored from scanned images of plants grown on ½ X MS plates using ImageJ [68]. To visualize *rpk2* meristems, roots were stained with Lugol's stain, cleared, and imaged using a 20 X objective on a Leica DMRXA microscope with DIC [69]. *clv1/bam1/bam2/bam3* roots were stained with 15 mM propidium iodide and imaged using a C-Apochromat 40 X/1.20 W Korr objective on a Zeiss LSM710 microscope. Excitation and emission windows for propidium iodide were 560 nm and 566-719 nm respectively. Confocal images were analyzed and processed using ImageJ and Adobe Photoshop.

GUS staining and imaging

Physcomitrella plants grown on BCDAT were cut out of plates with agar and incubated at 37°C in a 100 mM phosphate buffer with 10 mM Tris pH8.0, 1 mM EDTA pH8.0, 0.05% Triton X-100, 1 mg/mL X-GlcA (5-Bromo-4-chloro-3-indolyl-β-D-glucuronic acid) and potassium ferri/ferrocyanide using concentrations and times indicated in Figure 2 and legend. Plants were bleached in 70% ethanol and dissected and mounted in 0.3% low melting point agarose prior to imaging with a Keyence VHX-1000 digital microscope with a 0-50 X or a 50-200 X objective.

CLE peptide application

Synthetic CLE peptides (Genecust, >95% purity) were dissolved in phosphate buffer (50 μm, pH6.8) to stock concentrations of 1 mM and 10 mM. Plants were grown on BCDAT plates containing peptides diluted to concentrations specified in the main text.

QUANTIFICATION AND STATISTICAL ANALYSIS

Quantification and statistical analyses were undertaken as stipulated in main text and SI figures and figure legends.

DATA AND SOFTWARE AVAILABILITY

Genome and transcriptome data were searched as described in Method Details and details of data repositories are listed in Table S2.

Current Biology, Volume *28*

Supplemental Information

CLAVATA Was a Genetic Novelty for the Morphological Innovation of 3D Growth in Land Plants

Chris D. Whitewoods, Joseph Cammarata, Zoe Nemec Venza, Stephanie Sang, Ashley D. Crook, Tsuyoshi Aoyama, Xiao Y. Wang, Manuel Waller, Yasuko Kamisugi, Andrew C. Cuming, Péter Szövényi, Zachary L. Nimchuk, Adrienne H.K. Roeder, Michael J. Scanlon, and C. Jill Harrison

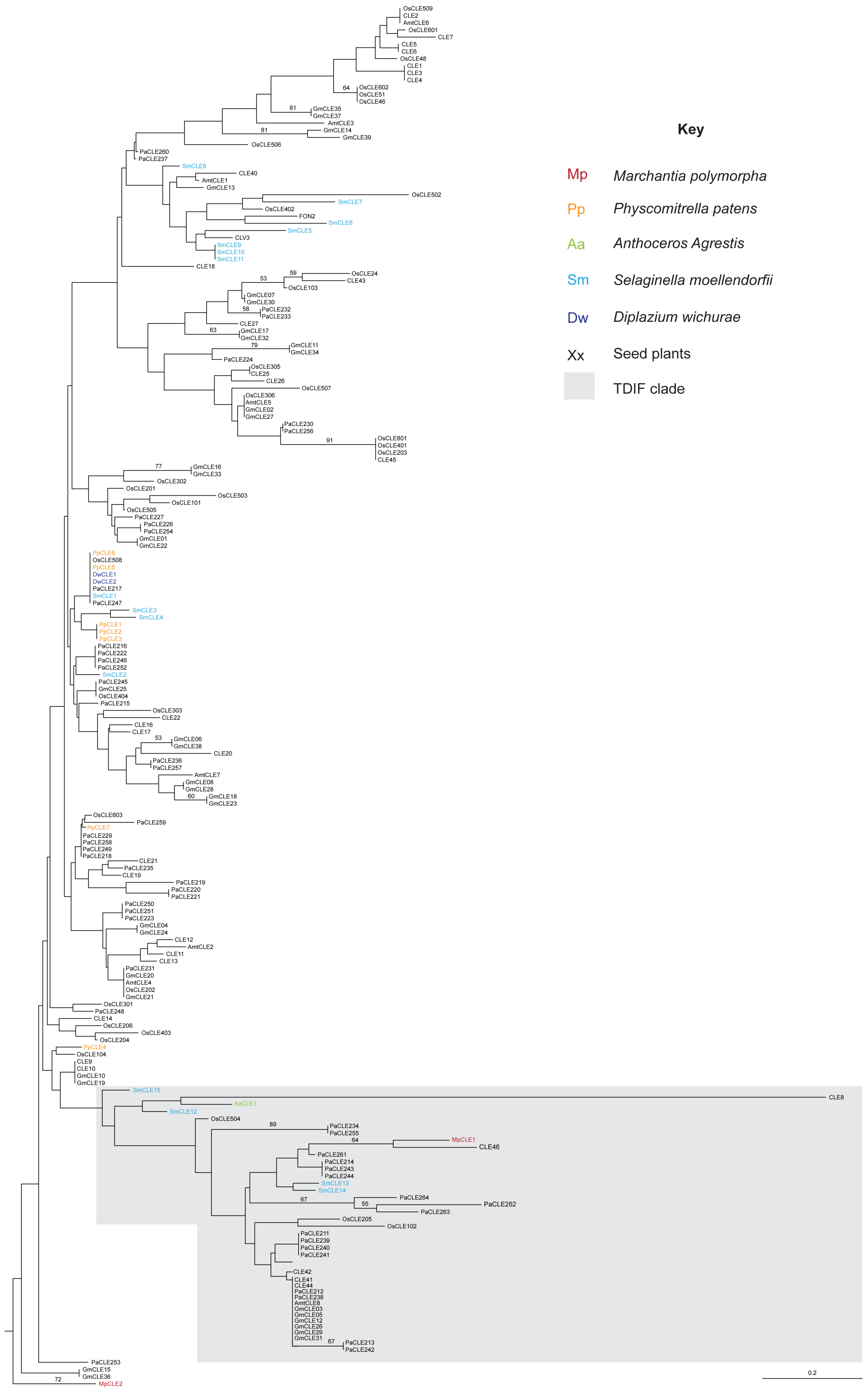

Figure S1: NJ tree showing relationships between land plant CLEs (related to Figure 1). 193 CLE motifs were aligned and a neighbour-joining analysis was undertaken as described in SI Methods. A *Marchantia polymorpha* CLE was used to root the tree, *Physcomitrella patens* was selected to represent mosses, *Anthoceros agrestis* was selected to represent hornworts, *Selaginella moellendorfii* was selected to represent lycophytes, *Diplazium wichurae* was selected to represent monilophytes, and seed plant sequences were retreived from *Picea abies*, *Amborella trichopoda*, *Glycine max*, *Oryza sativa* and *Arabidopsis thaliana*. 100 bootstrap replicates were performed, but bootstrap support was very low as there are few characters, and support values of > 50 are shown.

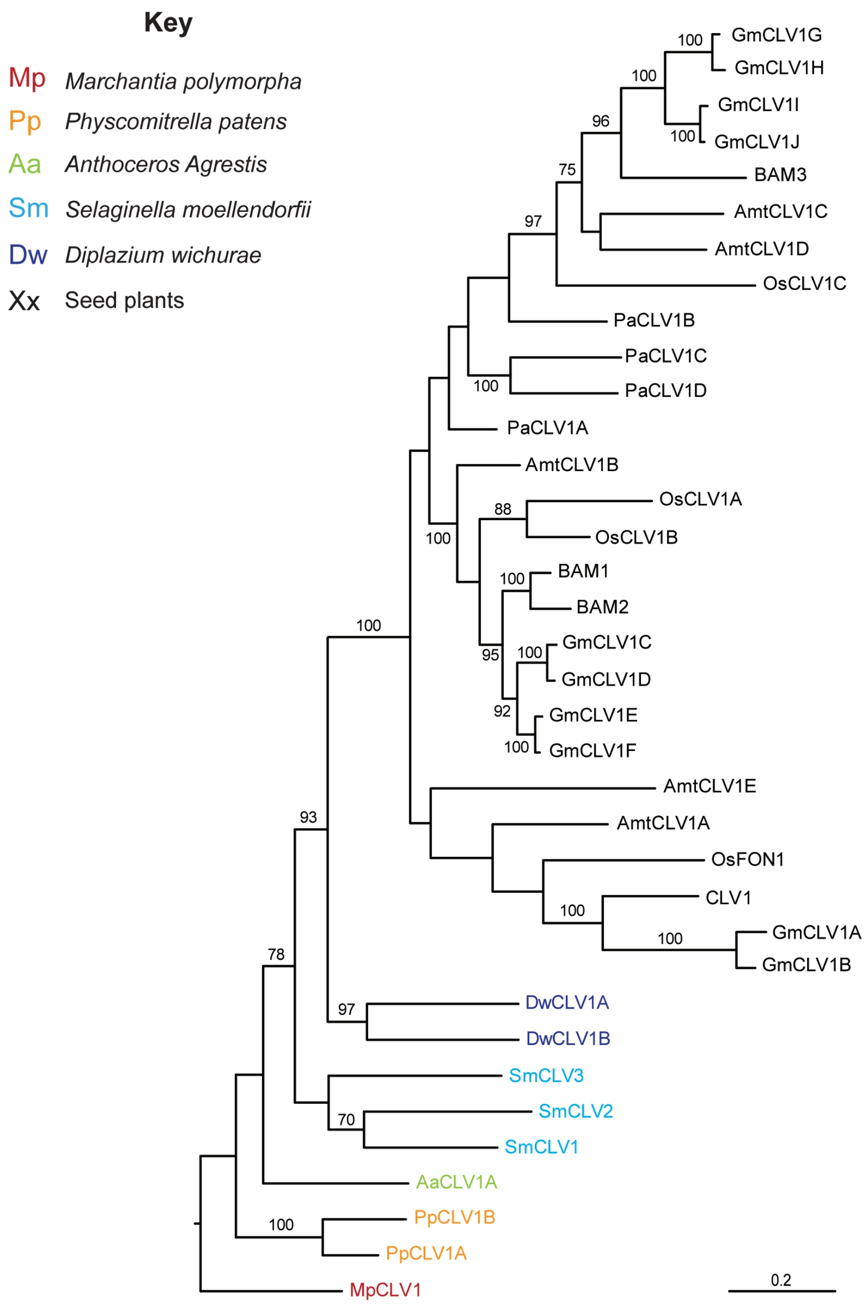

Figure S2: ML tree showing relationships between land plant BAM/CLV1-like proteins (related to Figure 1). 36 BAM/CLV1-like receptor-like kinase sequences were aligned, and data were analysed using the maximum likelihood method with the JTT matrix-based model as described in SI Methods. All positions containing gaps and missing data were removed prior to analysis, leaving a total of 525 in the final dataset. The tree with the highest log likelihood is shown. *Marchantia polymorpha* was sampled to represent liverworts, *Physcomitrella patens* was sampled to represent mosses, *Anthoceros agrestis* was selected to represent hornworts, *Selaginella moellendorfii* was selected to represent lycophytes, *Diplazium wichurae* was selected to represent monilophytes, and seed plant sequences were retreived from *Picea abies*, *Amborella trichopoda*, *Glycine max*, *Oryza sativa* and *Arabidopsis thaliana*. The tree was rooted on MpCLV1 in line with current estimates of land plant phylogeny, and bootstrap values of > 70 from 100 replicates are shown next to branches. Branch lengths represent the number of substitutions per site.

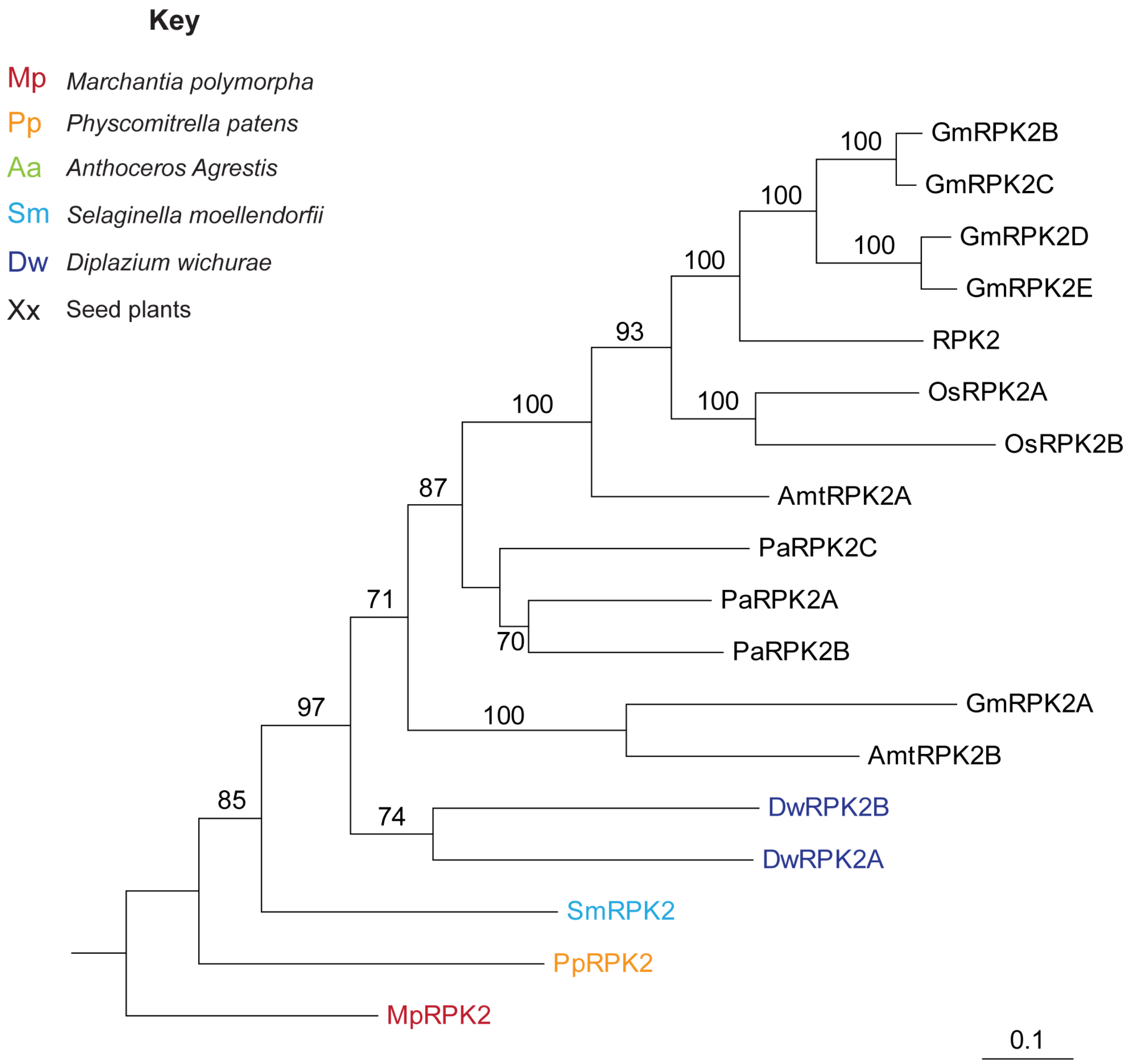

Figure S3: ML tree showing relationships between land plant RPK2-like proteins (related to Figure 1). 18 RPK2-like receptor-like kinase sequences were aligned and the phylogenetic tree was reconstructed using the maximum likelihood method with the JTT matrix-based model as described in SI Methods. All positions containing gaps and missing data were removed prior to analysis, leaving a total of 782 in the final dataset. The tree with the highest log likelihood is shown. *Marchantia polymorpha* was sampled to represent liverworts, *Physcomitrella patens* was sampled to represent mosses, *Anthoceros agrestis* was selected to represent hornworts, *Selaginella moellendorfii* was selected to represent lycophytes, *Diplazium wichurae* was selected to represent monilophytes, and seed plant sequences were retreived from *Picea abies*, *Amborella trichopoda*, *Glycine max*, *Oryza sativa* and *Arabidopsis thaliana*. The tree was rooted on MpRPK2 in line with current estimates of land plant phylogeny, and bootstrap values of > 70 from 100 replicates are shown next to branches. Branch lengths represent the number of substitutions per site.

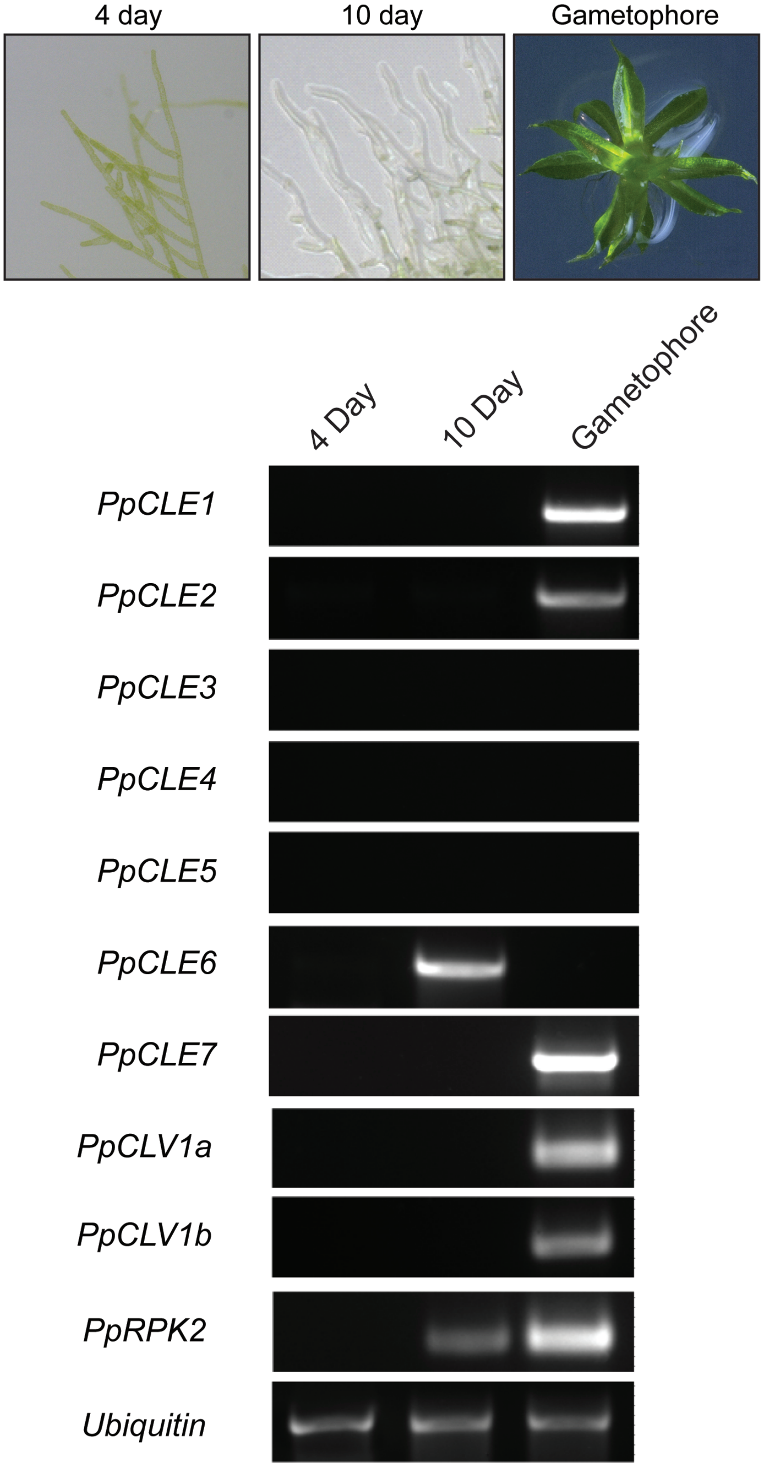

Figure S4: Expression of CLV signalling components in *Physcomitrella* gametophytic tissues as evaluated by RT-PCR (related to Figure 2). *PpCLE*s *1*, *2* and *7* were expressed in gametophores. *PpCLV1a*, *PpCLV1b* and *PpRPK2* were also expressed in gametophores and *PpRPK2* expression was also detected in 10-day old protonemal tissues, which is when gametophores first start to initiate under our growth conditions.

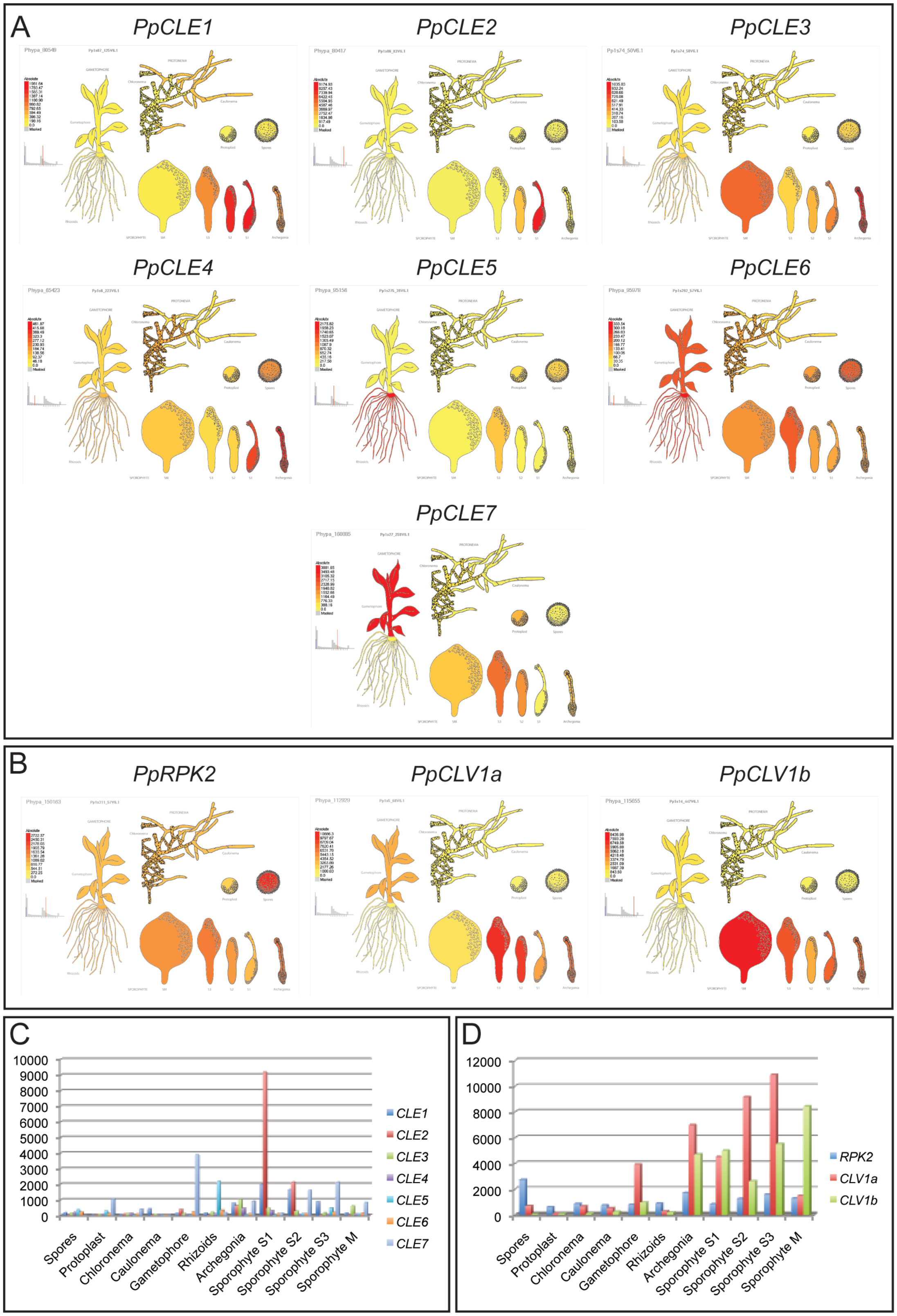

Figure S5: Expression of CLV signalling components in *Physcomitrella* tissues evaluated by eFP Browser data [S1] (related to Figure 2). (A) Expression patterns of *PpCLE*s *1-7*. (B) Expression patterns of receptor components. (C) Quantitative comparison of *PpCLE1-7* expression levels. (D) Quantitative comparison of *PpCLV1a*, *PpCLV1b* and *PpRPK2* expression levels.

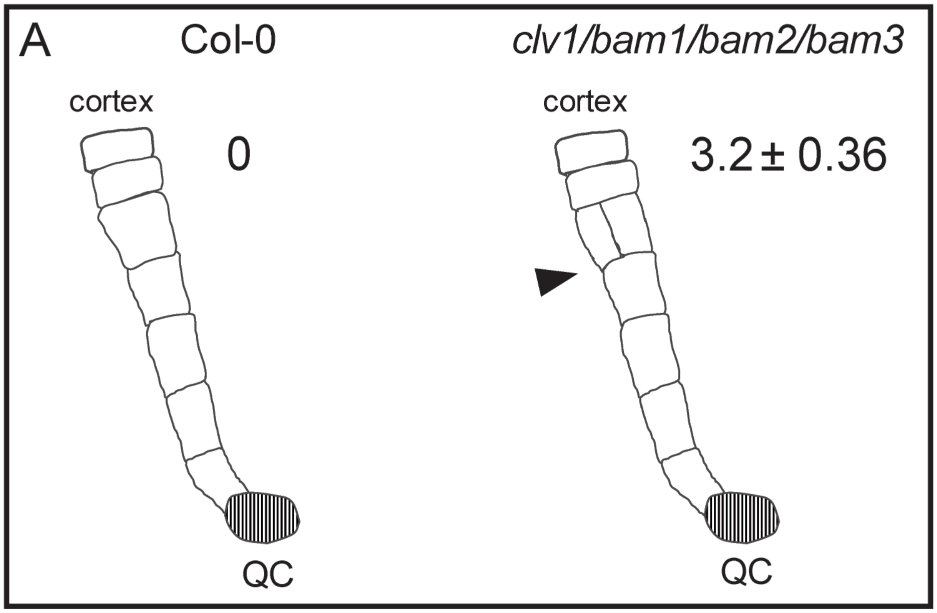

B

Root Length (cm) at time of imaging (stage-matched)		
**Col-0**		***clv1/bam1/bam2/bam3***
1.375	*Mean*	1.499
0.344	*SD*	0.33
0.661	*Min*	1.006
1.999	*Max*	2.165
19	*N*	13
0.078919012	*SE*	0.091525532

C

**Col-0**			***clv1/bam1/bam2/bam3***		
Label	*Meristematic zone Length (pixels)*	*Number of PCDs*	*Label*	*Meristematic zone Length (pixels)*	*Number of PCDs*
C3	650.308	0	Q1	636.572	3
C4	601.415	0	Q3	595.238	4
C5	584.411	0	Q6	586.276	1
C6	586.464	0	Q7	600.536	4
C7	609.908	0	Q8	635.437	3
C8	639.465	0	Q9	630.149	2
C10	558.516	0	Q10	586.464	4
C11	631.341	0	Q11	600.087	5
			Q12	619.681	3
			Q13	648.278	3
**Mean**	**607.729**	**0**	**Mean**	**613.872**	**3.2**
SD	31.26	**0**	SD	22.829	**1.135**

Figure S6: Quantification of cell division plane orientation defects in ground tissue layers in *Arabidopsis clv1/bam1/bam2/bam3* roots (related to Figure 3). (A) Diagram showing the nature of cell division plane orientation defects in wild-type versus *clv1/bam1/bam2/bam3* plants. (B) Equivalence of developmental stages used in comparisons between wild-type and mutant plants. (C) Quantitative data showing differences in the number of periclinal cell divisions (PCDs) in wild-type versus mutant plants.

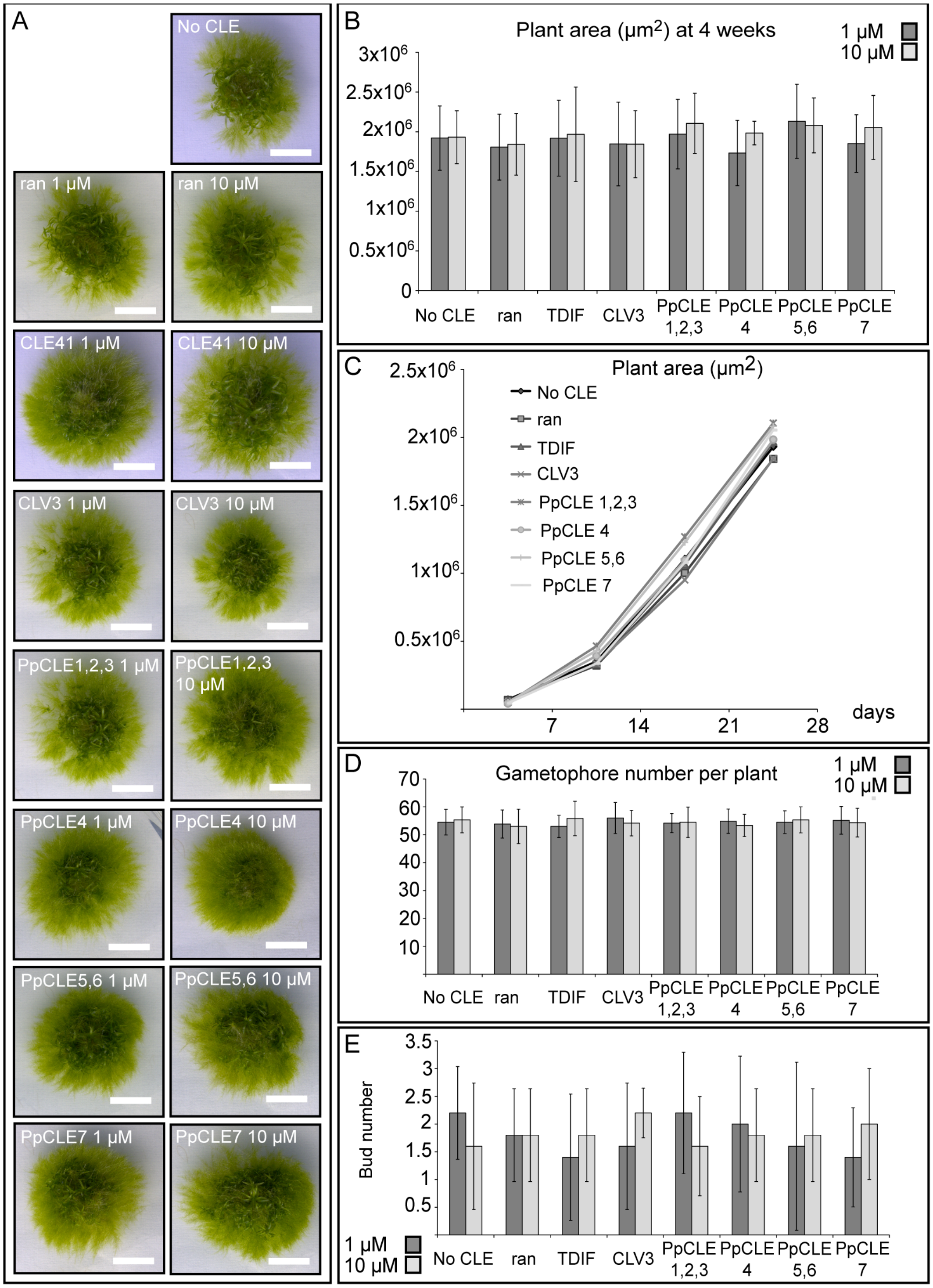

Figure S7: Treatment with CLE peptides had no appreciable effect on protonemal morphology (related to Figure 6). (A) Light micrographs of plants treated with a synthetic random peptide, CLE41, CLV3 or *Physcomitrella* CLEs showing morphology. Scale Bar = 1 cm. (B and C) The area of plants treated with 1 μM or 10 μM CLEs was no different from controls ($n \geq 20$). (D and E) CLE treatment did not effect gametophore initiation. For D, gametophores from 5 plants were counted for each treatment, and for E, a 5 mm square from the edge of 5 plants was counted for each treatment.

Gene name	Gene ID	Source
CLE		
PpCLE1	Pp1s87_125V6.1	*Physcomitrella patens*
PpCLE2	Pp1s86_83V6.1	*Physcomitrella patens*
PpCLE3	Pp1s74_50V6.1	*Physcomitrella patens*
PpCLE4	Pp1s6_223V6.1	*Physcomitrella patens*
PpCLE5	Pp1s275_39V6.1	*Physcomitrella patens*
PpCLE6	Pp1s292_57V6.1	*Physcomitrella patens*
PpCLE7	Pp1s27_259V6.1	*Physcomitrella patens*
AaCLE1	MG571535	*Anthoceros agrestis*
DwCLE1	scaffold-UFJN-2003654-Diplazium_wichurae	*Diplazium wichurae*
DwCLE2	scaffold-UFJN-2087917-Diplazium_wichurae	*Diplazium wichurae*
AmtCLE1	>lcllevm_27.model.AmTr_v1.0_scaffold00022.262	*Amborella trichopoda*
AmtCLE2	>lcllevm_27.model.AmTr_v1.0_scaffold00030.123	*Amborella trichopoda*
AmtCLE3	>lcllevm_27.model.AmTr_v1.0_scaffold00021.96	*Amborella trichopoda*
AmtCLE4	>lcllevm_27.model.AmTr_v1.0_scaffold00007.246	*Amborella trichopoda*
AmtCLE5	>lcllevm_27.model.AmTr_v1.0_scaffold00002.301	*Amborella trichopoda*
AmtCLE6	>lcllevm_27.model.AmTr_v1.0_scaffold00021.106	*Amborella trichopoda*
AmtCLE7	>lcllevm_27.model.AmTr_v1.0_scaffold00010.83	*Amborella trichopoda*
AmtCLE8	>lcllevm_27.model.AmTr_v1.0_scaffold00067.109	*Amborella trichopoda*
CLV		
DwCLV1A	scaffold-UFJN-2012643	*Diplazium wichurae*
DwCLV1B	scaffold-UFJN-2014575	*Diplazium wichurae*
GmCLV1C	NP_001235065.1	*Glycine max*
GmCLV1D	NP_001237688.1	*Glycine max*
GmCLV1E	NP_001235080.1	*Glycine max*
GmCLV1F	NP_001237715.1	*Glycine max*
GmCLV1G	XP_003530709.1	*Glycine max*
GmCLV1H	XP_006602289.1	*Glycine max*
GmCLV1I	XP_003518489.2	*Glycine max*
GmCLV1J	XP_003545159.1	*Glycine max*
FON1	Os06g50340.1	*Oryza sativa*
OsCLV1A	Os03g0228800	*Oryza sativa*
OsCLV1B	Os07g0134200	*Oryza sativa*
OsCLV1C	Os05g0595950	*Oryza sativa*
PpCLV1A	Pp1s5_68V6.1	*Physcomitrella patens*
PpCLV1B	Pp1s14_447V6.1	*Physcomitrella patens*
PaCLV1A	MA_64117p0010	*Picea abies*
PaCLV1B	MA_943683p0010	*Picea abies*
PaCLV1C	MA_52165p0010	*Picea abies*
PaCLV1D	MA_120550p0010	*Picea abies*
SmCLV1	XP_002965214.1	*Selaginella moellendorffii*
SmCLV2	XP_002971751.1	*Selaginella moellendorffii*
SmCLV3	XP_002970036.1	*Selaginella moellendorffii*
AaCLV1A	MG571536	*Anthoceros agrestis*
AmtCLV1A	evm_27.model.AmTr_v1.0_scaffold00055.1	*Amborella trichopoda*
AmtCLV1B	evm_27.model.AmTr_v1.0_scaffold00033.36	*Amborella trichopoda*
AmtCLV1C	evm_27.model.AmTr_v1.0_scaffold00071.179	*Amborella trichopoda*
AmtCLV1D	evm_27.model.AmTr_v1.0_scaffold00068.165	*Amborella trichopoda*
AmtCLV1E	evm_27.model.AmTr_v1.0_scaffold00056.126	*Amborella trichopoda*
RPK2		
DwRPK2A	scaffold-UFJN_2014694	*Diplazium wichurae*
DwRPK2B	scaffold-UFJN_2002858	*Diplazium wichurae*
GmRPK2A	XP_003548492.2	*Glycine max*
GmRPK2B	XP_003530440.2	*Glycine max*
GmRPK2C	XP_003551760.1	*Glycine max*
GmRPK2D	XP_003543956.1	*Glycine max*
GmRPK2E	XP_003554916.1	*Glycine max*
OsRPK2A	Os07g0602700	*Oryza sativa*
OsRPK2B	Os03g0756200	*Oryza sativa*
PaRPK2A	MA_13025p0010	*Picea abies*
PaRPK2B	MA_10427820p0020	*Picea abies*
PaRPK2C	MA_129592p0010	*Picea abies*
SmRPK2	XP_002982473	*Selaginella moellendorffii*
AmtRPK2A	evm_27.model.AmTr_v1.0_scaffold00154.29	*Amborella trichopoda*
AmtRPK2B	evm_27.model.AmTr_v1.0_scaffold00016.228	*Amborella trichopoda*

Table S1: List of newly identified *CLE, CLV* and *RPK2* genes, gene IDs and species of origin (related to Figure 1).

Class	Species	Publication	Database searched
Seed plant	*Arabidopsis thaliana*	[S2] Strabala *et al.* (2006)	NCBI
Seed plant	*Oryza sativa*	[S3] Yu *et al.* (2002)	NCBI
Seed plant	*Glycine max*	[S4] Mortier *et al.* (2011)	NCBI
Seed plant	*Amborella trichopoda*	[S5] Amborella Genome Project (2013)	Phytozome
Seed plant	*Picea abies*	[S6] Strabala *et al.* (2014)	NCBI
Monilophyte	*Diplazium wichurae*	1kp project	1kp project
Lycophyte	*Selaginella moellendorffii*	[S7] Miwa *et al.* (2009)	NCBI
Hornwort	*Anthoceros agrestis*	Draft genome assembly (Szövényi)	Draft genome assembly (Szövényi)
Moss	*Physcomitrella patens*	[S7] Miwa *et al.* (2009)	COSMOSS
Liverwort	*Marchantia polymorpha*	[S8] Bowman *et al.* (2017)	Phytozome
Charophyte alga	*Coleochaete nitellarum*	1kp project	1kp project
Charophyte alga	*Spirogyra* sp.	[S9] Delaux *et al.* (2015)	Dunand lab
Charophyte alga	*Chara braunii*	Draft genome assembly (Rensing)	Draft genome assembly (Rensing)
Chlorophyte alga	*Ulva spp.*	1kp project	1kp project
Chlorophyte alga	*Chlamydomonas reinhardtii*	[S10] Merchant *et al.* (2007)	Phytozome
Chlorophyte alga	*Volvox carteri*	[S11] Prochnik *et al.* (2010)	Phytozome
Chlorophyte alga	*Ostreococcus tauri*	[S12] Palenik *et al.* (2007)	Phytozome
Chlorophyte alga	*Chlorella vulgaris*	[S13] Blanc *et al.* (2010)	Phytozome

Table S2: List of taxa, publications and databases searched for sequence data (related to Figure 1).

Gene name	V1.6 genome	V3 genome	Peptide encoded
PpCLE1	Pp1s87_125V6.1	Pp3c7_11040V1.1	PpCLE 1/2/3
PpCLE2	Pp1s86_83V6.1	Pp3c1_13720V1.1	PpCLE 1/2/3
PpCLE3	Pp1s74_50V6.1	Pp3c3_10020V1.1	PpCLE 1/2/3
PpCLE4	Pp1s6_223V6.1	Pp3c26_11430V1.1	PpCLE 4
PpCLE5	Pp1s275_39V6.1	Pp3c22_4590V1.1	PpCLE 5/6
PpCLE6	Pp1s292_57V6.1	Pp3c19_6950V1.1	PpCLE 5/6
PpCLE7	Pp1s27_259V6.1	Pp3c21_5600V1.1	PpCLE 7
PpCLE8	not found	Pp3c11_15310V1.1	PpCLE 1/2/3
PpCLE9	not found	Pp3c4_31330V1.1	PpCLE 1/2/3
PpCLV1a	Pp1s5_68V6.1	Pp3c13_13360V1.1	PpCLV1a
PpCLV1b	Pp1s14_447V6.1	Pp3c6_21940V1.1	PpCLV1b
PpRPK2	Pp1s311_57V6.1	Pp3c7_5570V1.1	PpRPK2

Table S3: List of *Physcomitrella* CLAVATA pathway V3 genome gene IDs (related to Figure 1).

Primer name	Primer sequence
A. Primers for RT-PCR	
cDNA synthesis primer [S14]	
Q_T	CCAGTGAGCAGAGTGACGAGGACTCGAGCT
PpCLE1	
PpCLE1F	GTAGCATTGAGGTTCACGACA
PpCLE1R	CACGGGAATATGACTTGAGA
PpCLE2	
PpCLE2F	CAGATGCGGTTGAGAAAGAGA
PpCLE2R	GACTTGAGACCGATTGCTGTT
PpCLE3	
PpCLE3F	GTAATCCTCGCCATTTTCCA
PpCLE3R	GGGTTTCGTGGATTCGTGAT
PpCLE4	
PpCLE4F	CGAAGGCAGACGACAGGTGA
PpCLE4R	GACCTGCGACCTGTTGCTATT
PpCLE5	
PpCLE5F	ACGTTGGTGCTGGATTGTGAT
PpCLE5R	TCTGCCTCCACATCCCAAAT
PpCLE6	
PpCLE6F	GTAGGAATGGTCGTCGTCGT
PpCLE6R	GAACCAAGCGCTTCGACAT
PpCLE7	
PpCLE7F	TGCTTGTCCATGGTGATTGT
PpCLE7R	CCCGACTGTGATCCAACTTT
PpCLV1a	
PpCLV1aF	CAACATCGCAATCCAGGCT
PpCLV1aR	CCACTCTCAGGACCAATACAA
PpCLV1b	
PpCLV1bF	GGCAATCTCCCCACCCT
PpCLV1bR	CTCCTCGTCCAAGCAGTCTA
PpRPK2	
PpRPK2F	GTGGACCCGTTTCGTGTGTT
PpRPK2R	GGCTGGTGGACCCTGATAA
PpUBI	
PpUbi-intF	GCCATGCAGATCTTCGTGAA
PpUbi-intR	CTACGCAGCCAAGAACCGA
B. Promoter::NGG construction	
PpCLE1	
CLE15'PF	gtttaaacGGCACCATCTCCATCACTATCT
CLE15'PR	gcgatcgccacgtgGGTAAGGCTCCATGCACCGT
PpCLE2	
CLE25'PF	CGCTGCTGATTCACCACCTCAA
CLE25'PR	GGCATAATGTGGGGAGAAGGA
PpCLE7	
CLE75'PF	CTTGTGACATTCTAATAAGTGCTTATCC
CLE75'PR	CCCTTCCGAAAAACTGATACCA
PpCLV1a	
CLV1aPF	tatggatccTCTGTCAAATTTATTACCACTT
CLV1aPR	tatggatccGAGGAAAGCATGAGCACTGA
PpCLV1b	
CLV1bPF	TTTTGGATCAGCCATCCCTATAAGGCTCAG
CLV1bPR	GGTTATTCATGTTTTCTAGACACTGTTGCT
PpRPK2	
RPK25'PF	cttaagATTATTTTTTGTTACCTTGTATTTT
RPK25'PR	gtttaaacTCTCCCCTAACTCCTCCTCA

C. Promoter::NGG screening primers

PlGF2	AGGACACCCTTTCCAAACACATT
PlGR1	AAAAACCAATCTGGGAATAGCTTG
G6TERM4F	TAGGGTTCTATAGGGTTTCGCTCA
CLE1SCREENR	ACAGATTGCAGTTCCGTATGCTC
CLE2SREENR	TAAGCATGCAGCTCTAGGAAACG
CLE7SCREENR	CCATTGGCTATTAAAATGGCTTGA
RPK2SCREENR	TCTCATTTGCAAGTATAATCCAAGC
CLV1ASCREENR	CGAGTGCAACGAGATTCAAA
CLV1BSCREENR	GCAATCGGACAGACCTTTGAGTA

D. PpcleAmiR *construction*

PpcleAmiR1-3	
123-I	gaTTGGGAACCATGCGGTCGGAGtctctcttttgtattcc
123-II	gaCTCCGACCGCATGGTTCCCAAtcaaagagaatcaatga
123-III	gaCTACGACCGCATGCTTCCCATtcacaggtcgtgatatg
123-IV	gaATGGGAAGCATGCGGTCGTAGtctacatatatattcct
amiR-A	CTGCAAGGCGATTAAGTTGGGTAAC
amiR-B	GCGGATAACAATTTCACACAGGAAACAG
PpcleAmiR4-7	
7-I	gaTTGAAGCGGATTAGGACCTGGtctctcttttgtattcc
7-II	gaCCAGGTCCTAATCCGCTTCAAtcaaagagaatcaatga
7-III	gaCCCGGTCCTAATCGGCTTCATtcacaggtcgtgatatg
7-IV	gaATGAAGCCGATTAGGACCGGGtctacatatatattcct
amiR-A	CTGCAAGGCGATTAAGTTGGGTAAC
amiR-B	GCGGATAACAATTTCACACAGGAAACAG

E. PpcleAmiR *screening*

Kanamycin resistance cassette	
Kan-F	GGCATGATTGAACAAGATGAT
Kan-R	TATCGGGAAACTACTCACACAT
Hygromycin resistance cassette	
Hyg-F	AGGGCGAAGAATCTCGTGCT
Hyg-R	GCTTAGCGAACTGTGGACGA
PpcleAmiR amplification	
AmiRscrF	CGGTCGGAGTCTCTCTTTTG
AmiRscrR	CGCTCGGTGTGTCGTAGATA
PpcleAmiR expression cassette	
UbiOCSF	GCCGAACCAGCTTTCTTGTA
UbiOCSR	GTTGAATGGTGCCCGTAACT
PpUBI CDS	
Pp-Ubi-intF	GCCATGCAGATCTTCGTGAA
Pp-Ubi-intR	CTACGCAGCCAAGAACCGA

F. Ppclv1a1b construction and sequencing

PpCLV1a sgRNA-1	GGCAGACAGTGCCCGAGGCTCTCT
PpCLV1a sgRNA-1*	AAACAGAGAGCCTCGGGCACTGTC
PpCLV1a sgRNA-2	GGCACCACGGGCATGTCCTGATAC
PpCLV1a sgRNA-2*	AAACGTATCAGGACATGCCCGTGG
PpCLV1b sgRNA	GGCAGAAGTGCGAGACCCTCTTC
PpCLV1b sgRNA*	AAACGAAGAGGGTCTCGCACTTC

G. Ppclv1a1b *screening*

PpCLV1a sgRNA targets fwd (exon 4)	AACGGCTCAATTCCTCCAGA
PpCLV1a sgRNA targets rev (exon 5)	TTAGACACTCCACCCTTGCG
PpCLV1b sgRNA target fwd	TGGAGAGACGCAACTTCCAT
PpCLV1b sgRNA target rev	TTAAGACGCCCCAAATCAGC

H. Pprpk2 *construction*	
5' flanking region	
PpRPK2-5'F	ATCGATGGCTCTGGAGGTGAGTGACA
PpRPK2-5'R	GTTTAAACAGTTCGAGACAACACAAGAATGC
3' flanking region	
PpRPK2-3'F	GTTTAAACGGCGCGCCGATGGTCGGCATAGTAAACG
PpRPK2-3'R	ATCGATCAGGACGACAAGGCGGA
I. Screening Pprpk2 *lines*	
Hygromycin resistance casette	
Hyg-F	AGGGCGAAGAATCTCGTGCT
Hyg-R	GCTTAGCGAACTGTGGACGA
3' integration site analysis	
Hygromycin-F	CGCACAATCCCACTATCCTT
PpRPK2downstream-R	CAAGAGTCAGCCAATGATGCA
PpRPK2 CDS	
PpRPK2F	GTGGACCCGTTTCGTGTGTT
PpRPK2R	GGCTGGTGGACCCTGATAA
PpUBI CDS	
PpUbi-intF	GCCATGCAGATCTTCGTGAA
PpUbi-intR	CTACGCAGCCAAGAACCGA
J. *Southern blot probes*	
PpRPK2 probe	
PpRPK2probe-F	GTGGACCCGTTTCGTGTGTT
PpRPK2probe-R	GGCTGGTGGACCCTGATAA
Hygromycin probe	
Hygprobe-F	CGCACAATCCCACTATCCTT
Hygprobe-R	GATGTTGGCGACCTCGTATT
K. Identification of Arabidopsis rpk-2 *homozygotes*	
dCAPS primers	
AtRPK2-BamHI-F	CACATCTTGAGAGATTTCTGCTTTGTAGGTGGATC
AtRPK2-BamHI-R	GAGAAGTCACTATGTTCATGGATAT

Table S4: List of primers used in this study (related to Figures 2-4 and STAR methods).

Supplemental references

S1. Ortiz-Ramírez, C., Hernandez-Coronado, M., Thamm, A., Catarino, B., Wang, M., Dolan, L., Feijó, J.A., and Becker, J.D. (2016). A transcriptome atlas of *Physcomitrella patens* provides insights into the evolution and development of land plants. Molecular Plant *9*, 205-220.

S2. Strabala, T.J., O'Donnell, P.J., Smit, A.-M., Ampomah-Dwamena, C., Martin, E.J., Netzler, N., Nieuwenhuizen, N.J., Quinn, B.D., Foote, H.C., and Hudson, K.R. (2006). Gain-of-function phenotypes of many CLAVATA3/ESR genes, including four new family members, correlate with tandem variations in the conserved CLAVATA3/ESR domain. Plant Physiology *140*, 1331-1344.

S3. Yu, J., Hu, S., Wang, J., Wong, G.K.-S., Li, S., Liu, B., Deng, Y., Dai, L., Zhou, Y., and Zhang, X. (2002). A draft sequence of the rice genome (*Oryza sativa* L. ssp. *indica*). Science *296*, 79-92.

S4. Mortier, V., Fenta, B.A., Martens, C., Rombauts, S., Holsters, M., Kunert, K., and Goormachtig, S. (2011). Search for nodulation-related CLE genes in the genome of *Glycine max*. Journal of Experimental Botany *62*, 2571-2583.

S5. Chamala, S., Chanderbali, A.S., Der, J.P., Lan, T., Walts, B., Albert, V.A., Leebens-Mack, J., Rounsley, S., Schuster, S.C., and Wing, R.A. (2013). Assembly and validation of the genome of the nonmodel basal angiosperm *Amborella*. Science *342*, 1516-1517.

S6. Strabala, T.J., Phillips, L., West, M., and Stanbra, L. (2014). Bioinformatic and phylogenetic analysis of the CLAVATA3/EMBRYO-SURROUNDING REGION (CLE) and the CLE-LIKE signal peptide genes in the Pinophyta. BMC plant biology *14*, 47.

S7. Miwa, H., Tamaki, T., Fukuda, H., and Sawa, S. (2009). Evolution of CLE signaling: origins of the CLV1 and SOL2/CRN receptor diversity. Plant signaling & behavior *4*, 477-481.

S8. Bowman, J.L., Kohchi, T., Yamato, K.T., Jenkins, J., Shu, S., Ishizaki, K., Yamaoka, S., Nishihama, R., Nakamura, Y., and Berger, F. et al. (2017). Insights into land plant evolution garnered from the *Marchantia polymorpha* genome. Cell *171*, 287-304. e215.

S9. Delaux, P-M., Radhakrishnan, G.V., Jayaraman, D., Cheema, J., Malbreil, M., Volkening, J.D., Sekimoto, H., Nishiyama, T., Melkonian, M., and Pokorny, L. (2015). Algal ancestor of land plants was preadapted for symbiosis. *PNAS 112*, 13390-13395.

S10. Merchant, S.S., Prochnik, S.E., Vallon, O., Harris, E.H., Karpowicz, S.J., Witman, G.B., Terry, A., Salamov, A., Fritz-Laylin, L.K., and Maréchal-Drouard, L. (2007). The *Chlamydomonas* genome reveals the evolution of key animal and plant functions. Science *318*, 245-250.

S11. Prochnik, S.E., Umen, J., Nedelcu, A.M., Hallmann, A., Miller, S.M., Nishii, I., Ferris, P., Kuo, A., Mitros, T., and Fritz-Laylin, L.K. (2010). Genomic analysis of organismal complexity in the multicellular green alga *Volvox carteri*. Science *329*, 223-226.

S12. Palenik, B., Grimwood, J., Aerts, A., Rouzé, P., Salamov, A., Putnam, N., Dupont, C., Jorgensen, R., Derelle, E., and Rombauts, S. (2007). The tiny eukaryote *Ostreococcus* provides genomic insights into the paradox of plankton speciation. PNAS *104*, 7705-7710.

S13. Blanc, G., Duncan, G., Agarkova, I., Borodovsky, M., Gurnon, J., Kuo, A., Lindquist, E., Lucas, S., Pangilinan, J., and Polle, J. (2010). The *Chlorella variabilis* NC64A genome reveals adaptation to photosymbiosis, coevolution with viruses, and cryptic sex. The Plant Cell *22*, 2943-2955.

S14. Frohman, M.H. (1995). Rapid amplification of cDNA ends. In PCR Primer, C.W. Diffenbach and G.S. Dveksler, eds. (Cold Spring Harbor, NY.: Cold Spring Harbor Laboratory Press), pp. 381–409.

S15. Ishikawa, M., Murata, T., Sato, Y., Nishiyama, T., Hiwatashi, Y., Imai, A., Kimura, M., Sugimoto, N., Akita, A., Oguri, Y., et al. (2011). *Physcomitrella* Cyclin-Dependent Kinase A links cell cycle reactivation to other cellular changes during reprogramming of leaf cells. The Plant Cell *23*, 2924-2938.

S16. Moody, L., Kelly, S., Coudert, Y., Nimchuk, Z., Harrison, C.J. and Langdale, J. (2018). Somatic hybridization provides segregating populations for the identification of causative mutations in sterile mutants of the moss *Physcomitrella patens*. New Phytologist *218*, 1270-1277.
